# Two-Photon Enzymatic Probes Visualizing Sub-cellular/Deep-brain Caspase Activities in Neurodegenerative Models

**DOI:** 10.1038/srep26385

**Published:** 2016-05-23

**Authors:** Linghui Qian, Cheng-Wu Zhang, Yanli Mao, Lin Li, Nengyue Gao, Kah-Leong Lim, Qing-Hua Xu, Shao Q. Yao

**Affiliations:** 1 Department of Chemistry, National University of Singapore, 117543, Singapore; 2 Key Laboratory of Flexible Electronics & Institute of Advanced Materials, Jiangsu National Synergistic Innovation Center for Advanced Materials (SICAM), Nanjing Tech University, Nanjing, 211816, P. R. China; 3 National Neuroscience Institute, 308433, Singapore

## Abstract

Caspases work as a double-edged sword in maintaining cell homeostasis. Highly regulated caspase activities are essential during animal development, but dysregulation might lead to different diseases, e.g. extreme caspase activation is known to promote neurodegeneration. At present, visualization of caspase activation has mostly remained at the cellular level, in part due to a lack of cell-permeable imaging probes capable of direct, real-time investigations of endogenous caspase activities in deep tissues. Herein, we report a suite of two-photon, small molecule/peptide probes which enable sensitive and dynamic imaging of individual caspase activities in neurodegenerative models under physiological conditions. With no apparent toxicity and the ability of imaging endogenous caspases both in different subcellular organelles of mammalian cells and in brain tissues, these probes serve as complementary tools to conventional histological analysis. They should facilitate future explorations of caspases at molecular, cellular and organism levels and inspire development of novel two-photon probes against other enzymes.

Caspases are aspartate-specific cysteine proteases that have attracted significant attention because of their vital roles in apoptosis and inflammation^1^,^2^. Dysregulation of these enzymes may cause a variety of diseases including neurodegenerative diseases^1^,^2^,^3^,^4^. For example, ischemic stroke was the first neurologic disease in which the activation of caspase-1 was documented^3^,^5^. In addition to such acute neurologic disease, there is also increasing evidence that suggests caspase-mediated apoptotic pathways might play dominant roles in chronic neurodegenerative diseases including Alzheimer’s and Parkinson’s disease^3^,^4^. When, where and how these enzymes are activated are however not clearly understood, especially at the tissue and organism levels. This is in part due to a lack of tools capable of directly imaging them in real time from deep tissues and small animals. Meanwhile, proteolytic activities are usually tightly regulated by physical compartmentalization of the protease in different subcellular organelles, as well as the presence of specific endogenous inhibitor(s)^6^,^7^,^8^. Traditional methods such as immunoassays and colorimetric/fluorimetric assays survey total expression levels and activities of caspases *in vitro*, but do not provide accurate information about their dynamics^9^. Consequently, controversies often exist in the precise interpretation of spatial-temporal activation of endogenous caspases as well as the exact subcellular distribution of active caspases^10^,^11^,^12^.

Fluorescence microscopy stands out with its ability to monitor biological targets/processes in native cellular environments with temporal and spatial resolution^13^. For example, FLICA (Fluorochrome-Labeled Inhibitor of Caspase) is commercially available to assess caspase activities in cells^14^. Since a FLICA probe itself is fluorescent, “no-wash” imaging is not possible, making it ill-suited for real-time applications. To circumvent this problem, quenched activity-based probes (*q*ABPs) have been developed^15^,^16^. The fluorescence of such probes is quenched internally and will be “Turned-ON” only upon the interaction with active caspases. The covalent modification of *q*ABPs on their targets, however, renders such methods limited in their sensitivity and downstream pathway exploration^17^. Substrate-based imaging probes, based on fluorescent proteins (FPs) or small molecules possessing aggregation-dependent fluorescence properties, provide a direct and real-time measurement of caspase activation with the ability of signal amplification^18^,^19^,^20^,^21^,^22^. Genetic manipulation with FPs and the two-step activation required to form fluorescent aggregates in the latter make these approaches ineffective for rapid imaging of endogenous caspase activation. By virtue of their chemical tractability (e.g., different molecular structures/designs can be installed) and cell permeability, small molecule-based probes capable of being rapidly “Turned-On” in a single step in caspase activity-dependent manner, are highly desirable, especially if they can also be readily converted into organelle-specific probes for subcellular investigations of caspase activation^15^. Another problem in caspase probe development is the limited availability of highly selective/sensitive probes towards individual caspases. Such problem is even more severe for caspase-1, which is well-known to have a low expression level and a short half-life in most cells and tissues, and therefore is often overlooked in the presence of other caspases (e.g., caspase-3/-7)^23^. As such, recent research efforts have primarily focused on using optimized peptide/peptoid sequences for improved substrate specificity, but only a few of them were successfully applied in fluorescence imaging of endogenous caspase activities^16^,^24^,^25^,^26^. In a process referred to as “reverse design”, Pralnacasan (a non-peptide small molecule caspase-1 inhibitor; Fig. 1) was converted into a bioluminescent probe which exhibited ~1000-fold increase in sensitivity compared to the commercially available fluorogenic peptide substrate^27^. A coupled protein-probe engineering approach with mutant caspases was recently proposed to further improve the sensitive and specific detection of caspase-1^28^. Notably, none of the methods reported thus far, except in rare cases where sophisticated microscopic setups were used^21^, has been used in deep-tissue imaging. We therefore sought to develop cell-permeable, small molecule/peptide imaging probes capable of imaging individual caspases in real time directly from deep tissues by using two-photon fluorescence microscopy (TPFM). To our knowledge, small-molecule imaging probes dedicated for TPFM of caspases are currently unavailable.

TPFM, a technique now available in most fluorescence imaging facilities, offers the distinct advantage of deeper light penetration when compared to traditional one-photon fluorescence microscopy. Furthermore, with localized excitation volume, TPFM excels at three-dimensional (3D) imaging of biological specimens with significantly lower photo-bleaching and reduced photo-damage, and therefore is ideally suited for imaging deep tissues and small animals^29^. Despite recent progress, one of the most under-developed areas in TPFM is the availability of cell-permeable probes capable of imaging different enzymatic activities^15^,^30^,^31^,^32^,^33^,^34^,^35^,^36^. Two-photon imaging of caspases has thus far been demonstrated only with FPs and nanoparticles^19^,^37^. Herein, we report a suite of two-photon cell-permeable probes suitable for sensitive and real-time imaging of caspase activities *in situ* under “no-wash” conditions (Fig. 1). With peptide-based probes targeting individual caspase (i.e., caspase-1, -3/-7, -6, -8 or -9), their switchable two-photon properties were confirmed and most of them were shown to be readily taken up by mammalian cells and had low cell toxicity. Mindful of the current limit that many imaging studies of endogenous caspase activities rely heavily on organelle-specific fluorescent trackers to indirectly track the targeted caspase and its subcellular distribution^16^, we further addressed such issues with the successful development of organelle-directed probes by using caspase-3/-7 as representative examples (Fig. 1b); with **C3RM** and **C3RE**, these two probes were directly delivered to their respective subcellular organelles of interest (mitochondria and endoplasmic reticulum, respectively), where their two-photon fluorescence would be “Turned-On” only upon the presence of endogenous active caspase-3/-7. In the current study, we have used them to successfully and unambiguously monitor the spatio-temporal activation of caspase-3/-7 in response to suitable stimulation. Next, by combining the previously reported “reverse-design” concept with a highly potent small molecule caspase-1 inhibitor (Pralnacasan)^27^, together with the modular design of our two-photon probes, we have successfully developed two caspase-1-detecting small molecule probes, **C1RS** and **C1FS**, which showed improved capability in imaging endogenous caspase-1 activities. Finally, to show our newly developed two-photon caspase-detecting probes could provide complementary tools to commercially available coumarin-based peptide probes (which are commonly used in one-photon imaging experiments, but have very poor two-photon properties), representative probes were tested in live mammalian cells and fresh mouse brains of neurodegenerative models (Fig. 1c). Our results indicate these caspase-specifc probes could potientially be utilized to directly reveal the pathogenesis of diseased tissues by using two-photon fluorescence microscopy.

## Results and Discussions

### Design and synthesis of two-photon caspase probes

**Figure 1:**
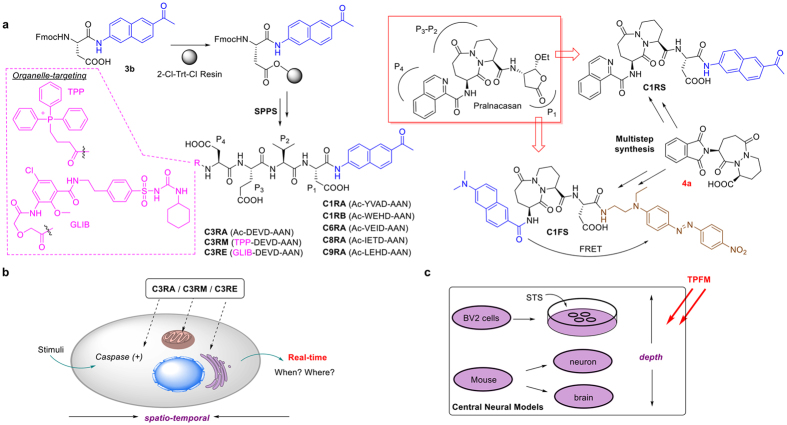
Overview of the design and synthesis of two-photon enzymatic probes for caspases. (**a**) Various peptide-based caspase probes and their brief synthetic scheme (left), and structures of the two small molecule caspase-1 probes, **C1RS** & **C1FS**, “reverse designed” from a known caspase-1 inhibitor, Pralnacasan (boxed). TPP and GLIB denote the corresponding organelle-targeting moieties in **C3RM** (targeting mitochondria) and **C3RE** (ER-specific), respectively. Selected probes were applied to detect caspase activation, including (**b**) real-time imaging of caspase-3-like activities in stimulated live cells using organelle-targeting probes (**C3RM**/**C3RE**) with a focus on the subcellular dynamics and (**c**) TPFM of endogenous caspase activation in central neural systems including live BV2 cells, mouse primary cortical neurons and fresh brain tissues with improved depth.

As shown in Fig. 1, 2-acetyl-6-aminonaphthalene (**AAN**) and 6-(dimethylamino)-2-naphthoic acid (**DAN**) were used as the two-photon reporters in our probe design^29^,^32^,^33^. By taking cue from substrate-based probes previously developed for one-photon imaging of caspases^20^,^21^, we attached a tetrapeptide substrate warhead (WH) to **AAN** *via* an amide linkage, to generate a total of six different peptide-based probes, each of which was intended to target a specific caspase. We reckoned that, by deliberately choosing these well-known substrate sequences (some are known to possess insufficient selectivity), their well-behaved caspase reactivity, cell permeability, as well as the availability of a large body of literature, could serve as an ideal platform for direct comparison with our newly designed two-photon probes. We named these probes **C1RA** (Ac-YVAD-AAN) & **C1RB** (Ac-WEHD-AAN), **C3RA** (Ac-DEVD-AAN), **C6RA** (Ac-VEID-AAN), **C8RA** (Ac-IETD-AAN) and **C9RA** (Ac-LEHD-AAN), which would be used to image caspase-1, -3/-7, -6, -8 and -9, respectively. Previously, **AAN** had been used to design other fluorogenic and/or ratiometric small molecule probes^29^. Our report herein however represents the first time these reporters have been used in the development of protease-responsive probes. By linking a caspase WH to **AAN** *via* an amide linkage, the electron-withdrawing amide bond would effectively quench the fluorescence of **AAN** *via* internal charge transfer (ICT; Supplementary Fig. 1a)^29^, rendering the probes non-fluorescent. After proteolytic cleavage of the WH by active caspases to liberate the free **AAN**, these probes would be “Turned-ON” due to the instantaneous fluorescence enhancement of the two photon-active free **AAN** which makes them suitable for real-time imaging in deep tissues. In addition to making two-photon-enabled caspase probes, we were also keen to address some other outstanding issues in endogenous caspase imaging. Many endogenous proteases are highly compartmentalized and activated only when they are needed in order to fulfil their specific cellular functions^6^,^7^. Such precise spatial and temporal controls occur with most caspases as well, as one might expect, given their critical roles in maintaining cell homeostasis^1^,^2^. For example, it was recently shown that caspase-8 cleaves its substrates from the plasma membrane upon CD95 induction^38^. The spatial-temporal dynamics of other caspases, including caspase-3/-7, is surprisingly not well-understood and remains controversial. For instance, conflicting results related to cellular distributions of active caspase-3/-7, particularly inside mitochondria and endoplasm reticulum (ER) as part of the cell’s programmed death signalling, continue to receive much debate^10^,^11^,^12^,^16^,^18^,^39^,^40^. We were particularly interested to know how such caspases might behave especially during the final stages of apoptosis in differentiating neuronal cells. Probes targeting these two subcellular organelles were therefore designed by using well-known organelle-targeting small molecule moieties. Attachment of a cell-permeable, mitochondria-directing triphenylphosphonium (TPP) moiety to DEVD-AAN, giving **C3RM** (TPP-DEVD-AAN; Fig. 1a, left), was designed to specifically deliver the caspase-3/-7-detecting probe only to mitochondria^41^,^42^. Previously, Chang *et al*. had successfully delivered similarly TPP-conjugated small molecules sensors for live-cell imaging of mitochondria-localized H_2_O_2_^43^. Other mitochondria-directed probes with various types of small molecules/peptides modified with TPP are also well-documented in the literature^44^, reaffirming the success of our design and the expected mitochondria-directed property of **C3RM**. Another probe, **C3RE** (GLIB-DEVD-AAN) was similarly designed but with an attached glibenclamide (GLIB) moiety, which is well-known to bind to the sulphonylurea receptors of ATP-sensitive K^+^ channels located in the endoplasmic reticulum (ER)^45^. GLIB has been widely used as a ER fluorescence Tracker (upon conjugation to a fluorophore)^46^,^47^, and therefore with the attachment of a relatively short peptide (i.e. DEVD-AAN), **C3RE** was expected to be delivered to ER exclusively and could be used to image caspase-3/-7 activities therein. The corresponding non-organelle-directed probe, **C3RA**, which doesn’t possess any specific organelle-targeting moiety, would be used in our experiments together with **C3RM**/**C3RE** to report endogenous caspase-3/-7 activities from the entire intracellular environment. With the help of real-time imaging, we hoped to obtain a clearer and more dynamic view of when and where endogenous caspase-3/-7 might be activated upon internal or external stimuli (Fig. 1b). With currently available immunoassays in which either pre-fractionated subcellular organelles followed by WB analysis, or immunofluorescence of fixed cells, is needed, inevitable cross-contamination of different organelles in the former and the static measurement of caspase activation in the latter, render both approaches highly limited^18^.

To expand the WH inventory for better selectivity and to design potentially more sensitive caspase-1-detecting probes, additional efforts were made to develop **C1RS**/**C1FS**; by taking cue from the “reverse design” strategy^27^, we replaced the tetrapeptide WH in **C1RA**/**C1RB** with Pralnacasan, providing **C1RS**. We also designed another Pralnacasan-derived probe, **C1FS** (Fig. 1a; right); instead of ICT, Förster Resonance Energy Transfer (FRET) was introduced in this probe to achieve a strong fluorescence Turn-ON effect upon caspase-1 cleavage. We reasoned that the P_4_-occupying isoquinolin-1-carboxylic acid in Pralnacasan could be replaced with the structurally similar **DAN**, whose fluorescence in **C1FS** could in turn be quenched intramolecularly by a dark quencher (i.e. disperse red-1) strategically inserted after the P_1_-Aspartic acid moiety of Pralnacasan.

Detailed synthesis of these probes is shown in Supplementary Schemes 1 & 2, and summarized in Fig 1a. Briefly, the eight peptide-based **AAN** probes were conveniently prepared by solid-phase peptide synthesis (SPPS) from the **AAN**-coupled Fmoc-Asp-OH, **3b**, with 2-chlorotrityl chloride (2-Cl-Trt-Cl) resin, by following standard Fmoc chemistry. For **C3RM** and **C3RE**, instead of capping the resin-bound tetrapeptide with acetic anhydride, (3-carboxypropyl)triphenylphosphonium bromide and an NHS ester of glibenclamide were used, respectively. For the synthesis of **C1RS** and **C1FS**, the key intermediate **4a** was used.

### Photophysical and biochemical properties of probes

**Figure 2:**
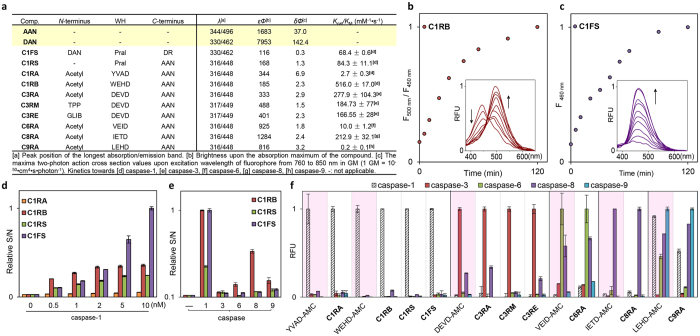
Photophysical and enzymatic properties of caspase probes. (**a**) Photophysical and kinetic properties of probes and free dyes. All measurements were taken in 50 mM HEPES, 50 mM NaCl, 0.1% CHAPS, 10 mM EDTA, 5% glycerol, 10 mM DTT, at *p*H = 7.2. (**b**) Time-dependent ratiometric fluorescence changes of **C1RB** (6 μM) incubated with caspase-1 (10 nM) over 2 h at room temperature. Data were obtained by plotting the normalized ratio of fluorescence intensity at 500 nm (F_500 nm_) over that at 450 nm (F_450 nm_). (Inset) time-dependent fluorescence emission spectra with *λ*_ex_ = 350 nm. (**c**) Time-dependent “Turn-ON” effect of **C1FS** (6 μM) incubated with caspase-1 (10 nM) measured and normalized at 460 nm (F_460 nm_) over 2 h at room temperature, with the corresponding fluorescence emission spectra (inset). *λ*_ex_ = 350 nm. (**d**) Normalized S/N ratio of each of the four caspase-1 probes (6 μM) incubated with 25 μg of BV2 lysates spiked with different concentrations of caspase-1 (37 °C, *t* = 2 h). S and N represent the fluorescence emission intensity of each reaction solution in the presence (S) and absence (N) of caspase-1. (**e**) Normalized S/N ratio of each of the 3 caspase-1 probes (**C1RB**/**C1RS**/**C1FS**; 6 μM) incubated with different caspases (*t* = 2 h, room temperature). (−) no caspase; (caspase-1) 1.5 nM; (caspase-3) 0.8 nM; (caspase-6) 0.2 μM; (caspase-8) 10 nM; (caspase-9) 0.5 μM. (**f**) Probe selectivity profiles with the corresponding commercial tetrapeptide-AMC as references. For each probe (6 μM), uniform conditions with different caspases were applied. Normalized, relative fluorescence (RFU) values were obtained after 2-h incubation at room temperature. For (**d**–**f**), *λ*_ex_ = 360 ± 40 nm; *λ*_em_ = 528 ± 20 nm, except for tetrapeptide-AMC and **C1FS** where *λ*_em_ = 460 ± 40 nm.

To establish that these newly developed two-photon enzymatic probes were indeed suitable for *in situ* imaging, we first investigated their photophysical and biochemical properties *in vitro* (Fig. 2). Under physiological conditions, the parental reporter **AAN** showed an emission maximum at 496 nm, whereas the dye-coupled probes (i.e. all probes except **C1FS**) exhibited a shift to ~448 nm (Fig. 2a and Supplementary Fig. 1c). The blue shift in *λ*_em_ likely came from changes in ICT across the conjugated naphthalene π-system caused by conversion of the electron-rich amine to an amide^29^. The neighboring peptide WHs having different amino acid side-chains may have conferred further quenching effects and produced discrepancy in fluorescence intensity amongst the probes. The fluorescence enhancement properties of these probes upon peptide removal were evident from the difference in quantum yields between **AAN** (*Φ* = 0.17) and the probes (*Φ* = 0.01–0.08). The blue-shifted property of these probes provided an additional advantage that they might be used in ratiometric imaging experiments (Fig. 2b), where simultaneous measurement of two signals resulting from reacted and unreacted forms of the probes in the same sample could be done, thus minimizing a major limitation commonly associated with intensity-based probes. Interestingly, the FRET-based probe **C1FS** exhibited similar excitation/emission peaks (*λ*_ex_ = 330 nm, *λ*_em_ = 462 nm) as its quencher-cleaved product **DAN** (in the form of a glycine methyl ester; Fig. 2a and Supplementary Fig. 1d). The significantly brighter one- and two-photon fluorescence emission of **DAN** (ε_330_·*Φ* = 7953, *δ·Φ* = 142.4 GM), when compared to **C1FS** (ε_330_·*Φ* = 116, *δ·Φ* = 0.3 GM), clearly demonstrated the Turn-ON effect of this probe upon proteolytic release of the quencher (Fig. 2c). We also noted the more favourable fluorescence properties of **DAN** over **AAN** (ε_344_·*Φ* = 1683, *δ·Φ* = 37.0 GM), which might be due to a greater delocalization of the lone-pair electrons in the dimethylamino group of **DAN** that stabilizes the dye’s excited state, indicating **C1FS** might offer more sensitive caspase-1 detection than **C1RS** and **C1RA**/**C1RB**.

Kinetic and substrate specificity studies with a panel of recombinant caspases under *in vitro* conditions were next carried out (Fig. 2a,f, Supplementary Table 1 and Supplementary Figs 1i–l and 2); all probes showed the expected caspase-responsive activities, with virtually identical selectivity and kinetic profiles as those obtained from commercial coumarin-based AMC substrates. Both small molecule probes, **C1RS** and **C1FS**, were proteolytically stable and did not display any noticeable degradation in BV2 cell lysates over 24 h (Supplementary Fig. 3). Both were also excellent caspase-1 substrates, with *K*_cat_/*K*_M_ values (84.3 ± 11.1 and 68.4 ± 0.6 mM^−1^s^−1^) falling between the two peptide-based substrates **C1RA** (Ac-YVAD-AAN; *K*_*cat*_/*K*_*M*_ = 2.7 ± 0.3 mM^−1^s^−1^) and **C1RB** (Ac-WEHD-AAN; *K*_*cat*_/*K*_*M*_ = 516.0 ± 17.0 mM^−1^ s^−1^). The WEHD sequence was previously shown to be a much better caspase-1 substrate than YVAD^48^. Of note, **C1RB** appeared to possess the lowest detection limit in detection caspase-1 activity, whilst **C1FS** gave the strongest S/N ratio at higher caspase-1 concentrations (>2 nM, Fig. 2d), likely due to its brighter fluorophore (i.e. **DAN**). In the substrate specificity studies where other caspases were present (Fig. 2e), both **C1RS** & **C1FS** showed improved selectivity over **C1RB** after a 2-h caspase treatment at room temperature, supporting the notion that small molecule probes might possess improved enzyme selectivity over peptide-based probes^27^. For subsequent two-photon, live-cell bioimaging, we chose **C1RB**/**C1FS**, **C3RA/C3RM/C3RE** and **C8RA** for caspase-1, -3/-7 and -8, respectively, as these probes possess the most ideal *in vitro* reactivity and selectivity profiles. We confirmed they were minimally cytotoxic (Supplementary Fig. 4) and cell-permeable (Supplementary Fig. 5a,c). In an artificial caspase-activation cell model, in which active recombinant caspases were individually delivered into NIH/3T3 cells, all probes were capable of imaging their intended caspase activities under “no-wash” conditions (Supplementary Fig. 5b); for caspase-1 detection, **C1FS** again showed higher contrast than **C1RB**, further confirming the improved sensitivity in **C1FS** as observed in our earlier *in vitro* studies.

### Bioimaging of active caspases in live cells

**Figure 3:**
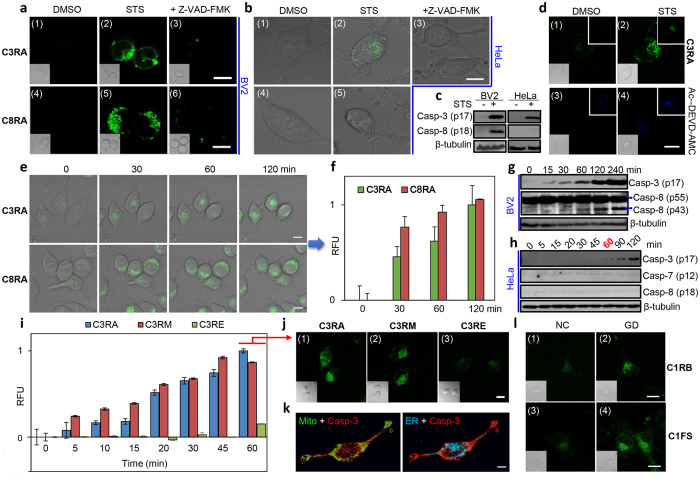
Bioimaging of active caspases in live cells. TPFM (*λ*_ex_ = 760 nm; *λ*_em_ = 480-550 nm for all **AAN**-probes) of STS-induced (**a**) BV2 cells (2 μM, 4 h) and (**b**) HeLa cells (1 μM, 4 h) using **C3RA**/**C8RA** (24 μM, 1 h). Panels 3/6: cells pretreated with Z-VAD(OMe)-FMK (50 μM, 1 h) before STS addition. Panels 1/4: normal cells (DMSO). (**c**) WB analysis of cleaved caspase-3/-8 from (**a**,**b**). (**d**) TPFM of normal/apoptotic Hela cells imaged by **C3RA** (24 μM, up) or Ac-DEVD-AMC (40 μM, down; *λ*_em_ = 420–500 nm). Panels 1/3: normal cells. Panels 2/4: STS-induced cells (1 μM, 4 h). Bottom inset: bright field. Top inset: one-photon images (*λ*_ex_ = 405 nm). (**e**) Real-time imaging of caspase-3/-8-like activities in live BV2 cells using 24 μM of **C3RA** (top)/**C8RA** (bottom) upon STS (2 μM) stimulation over 2 h. (**f**) Graphical quantification of RFU extracted from (**e**). (**g**) WB analysis of time-dependent caspase-3/-8 cleavage from STS-induced (2 μM) BV2 lysates. (**h**) WB analysis of time-dependent caspase-3/-7/-8 activation by detection of cleaved caspase-3/-7/-8 from lysates of HeLa cells stimulated with STS (2 μM). (**i**) RFU from real-time imaging of caspase-3-like activity in live HeLa cells with **C3RA**/**C3RM**/**C3RE** (24 μM, added 1 h before STS) upon STS (2 μM) stimulation. (**j**) Representative images (*t* = 60 min) from (**i**). (**k**) IF of STS-stimulated (2 μM, 2 h) HeLa cells by anti-caspase-3 (p17) antibody (red) with 3D projections of z-stack images at 90° view and merged with MitoTracker (green, left) or ER tracker (cyan, right). (**l**) TPFM of caspase-1 activation in primary cortical neurons upon glucose deprivation (GD, 8 h) followed by 1-h incubation with **C1RB** (24 μM) or **C1FS** (12 μM; *λ*_em_ = 450–550 nm). Inset: bright field images. NC: neuron cells incubated in normal neurobasal medium. Scale bar = 10 μm in all images.

We next used the probes to image endogenous caspases in well-established apoptosis and neural models (Fig. 3) under two-photon microscopic settings. It was suggested that microglia, the resident immune cells of the central nervous system, play prominent roles in the pathogenesis of neurodegenerative diseases^4^,^49^,^50^. Several caspases, including caspase-3/-7 and -8, were proposed to be involved in the regulation of microglia activation^50^. BV2 (an immortalized murine microglial cell line) was thus chosen as our neural model for real-time imaging of caspase activation in response to external stimuli. A previous report indicates lipopolysaccharide (LPS) (a ligand for Toll-like receptor 4, or TLR4) causes microglia activation but not cell death *via* activation of caspase-8-dependent caspase-3/-7 pathways^50^. We were therefore curious to know how BV2 might respond to other apoptosis-inducing stimulations. Staurosporine (STS), a potent pan-kinase inhibitor, is known to induce caspase-3 activation and apoptosis in a variety of mammalian cells including HeLa cells^15^,^51^. In our experiments, both BV2 and HeLa cells treated with STS showed obvious shrinkage in cell morphology and formation of cleaved casapse-3 fragment (p17) (Fig. 3a–c). **C3RA** was subsequently added to the cell medium followed by direct live-cell imaging; strong intracellular fluorescence was detected in cells after 4-h STS treatment but not in mock cells, and the fluorescence was inhibited by a pan-caspase inhibitor, Z-VAD(OMe)-FMK (Fig. 3a,b, top). When compared to **C3RA**, the commercial Ac-DEVD-AMC showed significantly poorer fluorescence properties for endogenous imaginging of caspase-3-like activities from live HeLa cells under two-photon settings (Fig. 3d). To detect whether any impromptu caspase-8 activation might have occurred under the same induction conditions, the entire experiment was repeated with **C8RA** together with the corresponding Western blotting (WB) analysis (Fig. 3a–c and Supplementary Fig. 5e,h); interestingly, while a death stimulus such as STS caused BV2 cells to undergo caspase-8 activation as shown in both the cellular imaging and WB results, no obvious caspase-8 activation was detected in HeLa cells under similar conditions. We further quantitatively analysed caspase activities in the lysates from the same STS-treated cells with the probes (Supplementary Fig. 5d,g); similar fluorescence increase profiles were obtained. To better illustrate the dynamics in caspase activation, real-time imaging of BV2 cells was next carried out (Fig. 3e). Live BV2 cells were pre-incubated with **C3RA** for 1 h, stimulated with STS then imaged. Caspase-8-like activity was imaged concurrently by using **C8RA**; fluorescence signals in both **C3RA** and **C8RA** channels started to develop in the cytosol of the treated cells within 30 min after STS was added, and slowly increased over the next 1.5 h (Fig. 3f). Consistent with the occurrence of apoptosis, we observed both cell shrinkage and formation of cleaved caspase-3 fragment (p17) (Fig. 3g). However, the same WB analysis carried out by using the corresponding antibody capable of detecting both the inactive uncleaved (p55) and the active fragmented (p43) caspase-8 revealed that no appreciable formation of the cleaved caspase-8 (p43) could be detected in the initial 30 min; this band started to emerge in the WB analysis ONLY at around 60-min time point, before becoming much more prominent at the 120-min time point. This clear discrepancy between our imaging-based and WB results was likely due to the limited sensitivity of the antibody used in the WB analysis, but also underlines the improved sensitivity of our newly developed probes and its potential application in live-cell imaging experiments. Our results herein are thus consistent with previously reported findings^50^, but offer a complementary method for more sensitive and dynamic imaging of caspase activities in live neural cells. Taken together, we concluded that **C3RA** and **C8RA** were indeed suitable for real-time two-photon imaging of endogenous caspase-3/-7 and -8 activities, respectively, in live mammalian cells.

As earlier mentioned, the two organelle-directed caspase-3/-7 probes, **C3RM** and **C3RE**, were designed to study whether compartmentalized caspase-3/-7 activation could be imaged as both probes were expected to accumulate selectively in their designated organelle, awaiting fluorescence “Turn-ON” by the corresponding caspase activated therein upon internal/external stimuli. Together with the non-organelle-directed probe, **C3RA**, we were hopeful that these three probes, when used in combination, would help in painting a more dynamic and spatially resolved view of when and where endogenous caspase-3/-7 might be activated, especially when compared to traditional immunoassays as earlier mentioned. It is worth noting that, direct live-cell measurement of the “OFF/ON” state from a pre-localized, caspase-detecting probe should in practice provide a more easily quantifiable means to measure locally activated caspase activities when compared to similar imaging experiments carried out with non-localized probes and organelle-specific fluorescent trackers. Again, STS-treated HeLa cells were used as a model in which mainly caspase-3 but not caspase-7 was activated (Fig. 3h and Supplementary Fig. 5f). Real-time imaging was carried out (Fig. 3i,j); gradual increases in intracellular fluorescence signals were recorded in both **C3RA**- and **C3RM**-treated cells within 60 min, while only background fluorescence was detected in **C3RE**-treated cells. Both the immunofluorescence (IF) with anti-active caspase-3 antibody (Fig. 3k and Supplementary Fig. 6) and WB analysis (Fig. 3h) showed unequivocal activation of endogenous caspase-3. No caspase-7 activation was detected over the same time course. Further co-localization analysis of the IF results indicated the presence of activated caspase-3 in both cytosol and mitochondria, but not in ER (See Supplementary Videos). We thus concluded that upon STS induction in HeLa cells, endogenous active caspase-3 appeared early in the mitochondria but not in the ER. Our results are thus similar to previously reported imaging data obtained from a mitochondria- targeted FP probe for caspase-3^18^. Compared to standard immunoassays, our organelle-directed imaging approach appeared more sensitive (compare Fig. 3h with Fig. 3i at *t* = 60 min), and offered additional spatial-temporal resolutions in real time.

Different from typical regulators of apoptosis, caspase-1 has long been known for its involvement in inflammation, and is primarily responsible for the activation of the key proinflammatory cytokine IL-1β^2^,^23^,^52^. Ischemic stroke was the first neurologic disease in which the activation of caspase-1 was documented and inflammation has also been often implicated in chronic neurodegenerative diseases^2^,^3^,^4^,^5^. However, the cellular mechanisms of how caspase-1 is involved in these processes remain poorly defined^28^. Since both **C1RB** and **C1FS** showed excellent two-photon “Turn-ON” properties in response to caspase-1 both *in vitro* and in an artificial cell model, we used them to image caspase-1 activation from a classic neural model by using glucose deprivation (GD) of cultured mouse primary neurons. GD simulates ischemia-like conditions and causes cell death *via* caspase-1-dependent pathways^53^,^54^. As shown in Fig. 3l, 8 h after GD treatment, followed by incubation with either **C1RB** or **C1FS**, we observed clear fluorescence signals from primary neurons, which were inhibited by Pralnacasan (Supplementary Fig. 7a). **C1FS** again delivered stronger signals with better S/N ratio than **C1RB**. The corresponding WB analysis further confirmed the presence of cleaved caspase-1, although the level of activation was not as obvious as our imaging results (Supplementary Fig. 7b). We also set up PMA-differentiated THP-1 cells stimulated with LPS/ATP (a classic inflammation model^55^), but despite many attempts, we were unable to detect reliable caspase-1 activation even with WB analysis/*in vitro* enzymatic assays. Imaging efforts with this model was thus not pursued further.

### Imaging endogenous caspase activities in deep tissues

**Figure 4:**
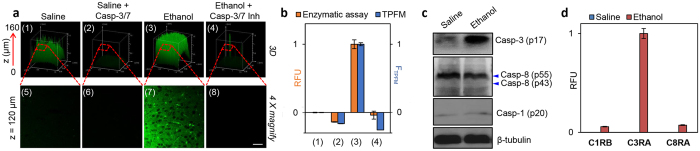
Imaging endogenous caspase activities in deep tissues. (**a**) TPFM of caspase-3 activation in brain slices of ethanol-intoxicated 7-day-old C57BL/6 mouse using **C3RA** (120 μM, 3 h). Top: 3D images of the mouse brain slice taken along the Z-axis from 0 to 160 μm at 10x magnification. Bottom: images taken at a depth of 120 μm at 40x magnification. Panels 1/5/2/6: control slices (saline-treated); panels 2/6/4/8: treated with caspase-3/7 inhibitor I (200 μM, 3 h). Scale bar = 50 μm. (**b**) RFU readings extracted from (**a**) (F_TPFM_, blue bars) *versus* those from *in vitro* enzymatic assays using lysates from the same brain slices (RFU, orange bars). The signal from saline controls was set as “0” and that from ethanol-treated samples was set as “1” in RFU quantification. Due to the base-level active caspase-3 in the saline controls, negative readings were shown with inhibitor-treated samples. (**c**) WB analysis of caspase-3/-1/-8 activation in lysates of the same mouse brain slices shown in (**a**). (**d**) Normalized RFU readings from *in vitro* enzymatic assays using brain lysates (300 μg) of saline/ethanol treated mice after incubation with **C1RB/C3RA/C8RA** (6 μM) for 1 h at 37 °C.

The biggest advantage of our newly developed imaging probes over existing substrate-based probes is their two-photon capability (Fig. 3d), which should render them suitable for real-time imaging of endogenous caspase activities directly from deep tissues. Increasing evidence reveals that caspases, especially caspase-3, play crucial roles in neural functions, although most conclusions were derived from static assays^56^. Ethanol-intoxicated infant mouse were previously shown, in immunohistological experiments, as an excellent animal model for *in vivo* studies of caspase-3-related neurodegeneration^57^,^58^. Seven-day-old C57BL/6 mice were subcutaneously injected with either ethanol or saline (mock control), and sacrificed 8 h later. The 200-μm-thick sections of the brain were dissected and incubated with **C3RA,** then imaged by TPFM. As shown in Fig. 4a, the Z-axis scanning allowed sensitive, real-time detection of caspase-3/-7-like activities to as deep as 160 μm of the tissues (Panels 1–4). Pre-treatments of the tissues with caspase-3/7 inhibitor I completely abolished the fluorescence. Further processing of TPFM at a single Z-plane (at 120 μm; Panels 5–8) provided quantitative fluorescence readings that showed consistent caspase-3 activation as those obtained from WB analysis and *in vitro* enzymatic assays with the corresponding tissue lysates (Fig. 4b,c; the signals from the mock controls, due to basal-level expression of caspase-3 activity, was set as “0” in RFU quantification). No obvious caspase-1 and -8 activations were detected in the same tissue samples probed with **C1RB** and **C8RA,** respectively, as well as by WB analysis (Fig. 4c,d). The TPFM images of deep tissues, coupled with the relatively low background fluorescence observed in these images, clearly demonstrated the feasibility of these newly developed probes in future imaging-based, real-time experiments in other tissues and small animals.

## Conclusion

In conclusion, we have successfully developed a suite of cell-permeable probes suitable for sensitive and real-time imaging of target caspase activities in classic neurodegenerative models with two-photon fluorescence microscopy. The two-photon capability of these probes were enabled by the use of suitable two-photon dyes (**AAN** and **DAN**), and with observed significant fluorescence enhancement upon probe cleavage in response to different caspases. “No-wash” imaging of caspase activation was carried out with the classic STS-induced apoptosis model in live BV2 and HeLa cells. An organelle-directed approach was further developed that enabled successful detection of mitochondria-localized active caspase-3 activities but not in ER within HeLa cells during 1 h. We believe this novel location-based strategy with **C3RM**/**C3RE** could be further explored to study the spatial dynamics of endogenous caspase-3/-7 upon different stimuli. With the feature of low background and high sensitivity endowed by the two-photon caspase-1-detecting probes, we have successfully imaged this enzyme in glucose-deprived mouse primary neurons. Finally, with the ability of directly imaging caspase activities from deep tissues, we observed apparent caspase-3 activation in ethanol-intoxicated mouse brains. Our results indicate these probes are useful tools for studying caspase activation in neurologic disease models. Future work will be directed at the development of more selective caspase probes (e.g., caspase-6/-9), and to extend the same design strategy to other classes of biologically important proteases.

## Methods

### Chemical synthesis

Detailed information is provided in Supplementary Information.

#### Solid-Phase Peptide Synthesis (SPPS)

The peptide was synthesized on 2-chlorotrityl chloride resin using Irori™ Microkan reactors following previously published proceduress^59^. Resulting probes were characterized using LC-MS (IT-TOF) and ESI-HRMS.

**C1RA** (Ac-YVAD-AAN, C_35_H_41_N_5_O_9_): *m/z*[M+H]^+^calcd, 676.2977; ESI-HRMS found, 676.3005.

**C1RB** (Ac-WEHD-AAN, C_40_H_42_N_8_O_10_): *m/z*[M+H]^+^calcd, 795.3097; ESI-HRMS found, 795.3114.

**C3RA** (Ac-DEVD-AAN, C_32_H_39_N_5_O_12_): m/z[M+Na]^+^calcd, 708.2487; ESI-HRMS found, 708.2503.

**C6RA** (Ac-VEID-AAN, C_34_H_45_N_5_O_10_): m/z[M+Na]^+^calcd, 706.3059; ESI-HRMS found, 706.3058.

**C8RA** (Ac-IETD-AAN, C_33_H_43_N_5_O_11_): m/z[M+Na]^+^calcd, 708.28519; ESI-HRMS found, 708.2863.

**C9RA** (Ac-LEHD-AAN, C_35_H_43_N_7_O_10_): m/z[M+Na]^+^calcd, 744.2964; ESI-HRMS found, 744.2961.

**C3RM** (TPP-DEVD-AAN, C_52_H_57_N_5_O_12_P^+^): m/z[M]^+^ calcd, 974.3736; ESI-HRMS found, 974.3733.

**C3RE** (GLIB-DEVD-AAN, Chemical Formula: C_57_H_68_ClN_9_O_19_S): m/z[M-3H]^3−^ calcd, 415.4607; found, 415.4610.

Preparation of Pralnacasan-derived small molecule probes was briefly introduced as follows.

**C1RS**: Compound **7** (10.2 mg, 0.014 mmol) dissolved in CH_2_Cl_2_ was added TFA (20% v/v) at 0 °C. The reaction was warmed up and further stirred at room temperature for 4 h. The reaction mixture was concentrated, and the resulting residue was purified by flash chromatography (EtOAc:Hexane = 50:1 with 1% acetic acid) to afford **C1RS** as pale brown solid (3.9 mg; 52% yield). ^1^H NMR (300 MHz, DMSO-*d*_*6*_): δ 9.05 (d, *J* = 9.0 Hz, 2H), 8.61–8.52 (m, 3H), 8.32 (s, 1H), 8.12–8.01 (m, 3H), 7.92–7.62 (m, 5H), 5.23 (d, *J* = 11.5 Hz, 1H), 4.92 (d, *J* = 23.1 Hz, 1H), 4.66 (d, *J* = 19.3 Hz, 1H), 4.37 (d, *J* = 9.5 Hz, 1H), 4.12 (t, *J* = 7.2 Hz, 1H), 3.92 (d, *J* = 5.6 Hz, 2H), 2.67 (s, 3H), 2.27 (t, *J* = 7.2 Hz, 2H), 2.19–2.05 (m, 4H), 1.98 (s, 2H).^13^C NMR (75 MHz, DMSO-*d*_*6*_): δ 198.0, 175.2, 172.0, 171.6, 170.1, 170.0, 165.9, 150.1, 141.2, 139.3, 137.0, 136.1, 133.3, 131.1, 130.8, 130.4, 129.1, 128.9, 128.0, 127.5, 127.0, 126.0, 124.4, 124.2, 121.1, 115.2, 52.6, 51.1, 48.8, 44.1, 36.2, 30.2, 29.7, 27.0, 26.1, 19.4. ESI-HRMS: *m/z*[M+Na]^+^calcd., 701.2330; found, 701.2358.

**C1FS**: To the solution of **F4** (97 mg, 0.12 mmol) in ethanol (10 mL) was added hydrazine hydrate (60 mg, 1.2 mmol). The resulting mixture was stirred at 60 °C for 2 h. Upon solvent evaporation, the residue containing the desired free amine was re-dissolved in DMF (2 mL). To a separate reaction vessel was added DMF (3 mL), 6-(dimethylamino)-2-naphthoic acid (40 mg, 0.18 mmol) and PyBOP (125 mg, 0.24 mmol). The resulting mixture was stirred at room temperature for 10 min. Then the above amine solution was added. The resulting mixture was stirred at room temperature overnight. Upon solvent evaporation, the resulting residue was added water (20 mL) and EtOAc (50 mL). The organic layer was washed with water (3 × 20 mL), brine (20 mL), dried over Na_2_SO_4_, filtered and concentrated, before being purified by flash chromatography (EtOAc:Hexane = 10:1 to 1:1) to give the coupling product which was further dissolved in a mixture of CH_2_Cl_2_ (5 mL) and TFA (5 mL). The mixture was stirred at room temperature for 8 h. Upon solvent evaporation, the resulting residue was purified by preparative TLC (CH_2_Cl_2_:MeOH = 20:1) to give the final product **C1FS** (11.4 mg, 11.2% yield). ^1^H NMR (300 MHz, DMSO-*d*_6_): δ 8.50 (d, *J* = 7.5 Hz, 1H), 8.44 (d, *J* = 7.5 Hz, 1H), 8.36 (m, 2H), 8.31 (brs, 1H), 8.15 (m, 1H), 7.93 (m, 2H), 7.79–7.85 (m, 4H), 7.69 (d, *J* = 8.5 Hz, 1H), 7.28 (dd, *J* = 2.5 Hz, *J* = 9.0 Hz, 1H), 6.95 (d, *J* = 2.0 Hz, 1H), 6.93 (d, *J* = 9.5 Hz, 2H), 5.13 (t, *J* = 5.0 Hz, 1H), 4.87–4.92 (m, 1H), 4.53 (q, *J* = 7.0 Hz, 1H), 4.41 (m, 1H), 3.12–3.37 (m, 3H) 3.40–3.50 (m, 4H), 3.04 (m, 6H), 2.94 (m, 1H), 2.62 (dd, *J* = 6.0 Hz, *J* = 16.0 Hz, 1H), 2.48 (m, 1H), 2.34 (m, 1H), 2.17–2.24 (m, 2H), 2.04 (m, 1H), 1.90 (m, 1H), 1.68 (m, 1H), 1.60 (m, 1H), 1.14 (t, *J* = 7.0 Hz, 3H).^13^C NMR (125 MHz, DMSO-*d*_6_):δ 172.3, 169.8, 166.9, 156.7, 152.0, 150.0, 147.2, 143.2, 136.7, 130.2, 128.1, 126.8, 126.6, 126.0, 125.4, 125.1, 124.9, 122.9, 117.1, 111.9, 105.4, 52.8, 50.3, 49.2, 49.1, 45.4, 41.4, 37.1, 30.4, 29.2, 25.9, 19.5, 12.5. ESI-HRMS: *m/z* [M+H]^+^calcd., 849.3678; found, 849.3716.

### *In vitro* Enzymatic Assay

All enzymatic assays were carried out in HEPES buffer (50 mM Hepes, 50 mM NaCl, 0.1% CHAPS, 10 mM EDTA, 5% glycerol, 10 mM DTT, *p*H = 7.2) supplemented with 0.02% Triton X-100 at 25 °C for recombinant caspases, and at 37 °C for lysates. Appropriate enzyme/lysate dilutions were added as indicated, to reaction solutions containing our probes at a final concentration of 6 μM in 384-well microplate (Greiner Bio-One #781900). Control experiments with commercial tetrapeptide-AMC substrates were done where indicated. For inhibitor treatments, Z-VAD(OMe)-FMK (50 μM, ab120487, Abcam) or caspase-3/7 inhibitor I (200 μM for tissue lysate, Calbiochem #218826) was pre-incubated with biological samples for 3 h at 4 °C, prior to incubation with the probe. Liberation of fluorescence (*λ*_ex_ = 360 ± 40 nm; *λ*_em_ = 528 ± 20 nm for **AAN**, and *λ*_em_ = 460 ± 40 nm for AMC and **DAN**) was recorded by using a BioTek Synergy 4 plate reader. Kinetic constants were computed by fitting the data to Michaelis-Menton equation using a non-linear regression *via* software Origin™. The error bar in graphs or “±” in the table is a standard deviation (s.d.) of the data.

### General procedures for one- and two-photon fluorescence imaging of live cells

NIH/3T3, HeLa and BV2 cell lines used in our experiments were cultured in DMEM medium supplemented with 10% (for NIH/3T3 and HeLa) or 5% (for BV2) fetal bovine serum (FBS), with 100.0 mg/L streptomycin and 100 IU/mL penicillin. Cells were seeded in glass-bottom dishes (Greiner Bio-One, #627870) and maintained in a humidified atmosphere of 5% CO_2_ at 37 °C overnight before being imaged. For STS induction, HeLa/BV2 cells were pretreated with/without inhibitor (50 μM Z-VAD(OMe)-FMK) for 1 h before stimulation with STS (LC Laboratories) or an equivalent volume of DMSO for 4 h. **C3RA** or **C8RA** (24 μM) was subsequently added and incubation was continued for another 1 h before image acquisition. For real-time imaging, HeLa/BV2 cells in glass-bottom dishes were pre-incubated with the probe for 1 h (**C3RM**/**CERE** was washed after 1-h incubation to remove any free probe outside of the targeted organelle), followed by addition of STS (2 μM) and immediate image acquisition (*t* = 0) over 2–2.5 h. A representative video clip showing real-time imaging was provided in the Supplementary Videos. For one- and two-photon microscopy, data were acquired on a Leica TCS SP5X Confocal Microscope System or on a Carl Zeiss LSM 510 Meta Confocal Microscope as indicated. All 3D images for immunofluorescence were acquired from PerkinElmer Ultraview Vox Spinning Disc confocal microscope and processed with Volocity 6.3.1 3D image analysis software.

### TPFM imaging of caspase activities in primary cortical neurons and fresh tissues

All two-photon images were taken on a Leica TCS SP5X Confocal Microscope System. Procedures involving animals were approved by and conformed to the guidelines of Institutional Animal Care and Use Committee at the National Neuroscience Institute (Singapore). Dissociated neuron-enriched cell cultures of cerebral cortex were established from day 16 C57BL/6 mouse embryos, as described^53^. Primary neurons were cultured in the neurobasal medium (Gibco) with 2% B27 and 0.5 mM GlutaMAX. Experiments were performed in 7–9 day-old cultures. For glucose-deprivation studies, glucose-free neurobasal medium was used (with other components fixed). The cultured neurons were incubated in glucose-free medium for 8 h, while control cells were incubated in normal neurobasal medium. After GD treatment for 8 h, **C1RB** (24 μM)/**C1FS** (12 μM) was introduced with further incubation for 1 h. To check whether the signal observed was related to caspase-1 activity, the caspase-1 specific inhibitor, Pralnacasan (50 μM) was added to the cultures 1 h prior to the probe (*t* = 7 h during GD). Tissues used in the imaging experiments were fresh brains of 7-day-old C57BL/6 mice subcutaneously injected with either ethanol (20% solution in normal saline with 2.5 g/kg at 0 h and again at 2 h) or the same amount of normal saline^57^,^58^. At 8 h following the first ethanol dose, the brains were surgically removed from the mouse head and immediately transferred into an ice-artificial cerebrospinal fluid (ACSF; 138.6 mM NaCl, 3.5 mM KCl, 21 mM NaHCO_3_, 0.6 mM NaH_2_PO_4_, 10 mM D-glucose, 1 mM CaCl_2_ and 3 mM MgCl_2_). The brain was cut into 200 μm-thick sections using a vibrating blade microtome in ACSF. Slices were incubated with **C3RA** (120 μM) in ACSF at 37 °C for 3 h before image acquisition. For inhibition experiments, the slices were treated with caspase-3/7 inhibitor I (200 μM) 3 h prior to addition of **C3RA**. Treated brains were then transferred to poly-L-lysine-coated cover slips and images were acquired at different depths by changing the Z-axis thickness on the microscope.

## Additional Information

**How to cite this article**: Qian, L. *et al*. Two-Photon Enzymatic Probes Visualizing Sub-cellular/Deep-brain Caspase Activities in Neurodegenerative Models. *Sci. Rep.* **6**, 26385; doi: 10.1038/srep26385 (2016).

## Supplementary Material

Supplementary Information

Supplementary Information

Supplementary Information

Supplementary Information

Supplementary Information

Supplementary Information
