# Supplementary material for: Two-Photon Enzymatic Probes Visualizing Sub-cellular/Deep-brain Caspase Activities in Neurodegenerative Models
Source: Sci Rep. 2016 May 23;6:26385. doi: 10.1038/srep26385 (PMC4876444; doi:10.1038/srep26385)
Supplement: Supplementary Information [file srep26385-s6.pdf]

Supplementary Information for

Two-Photon Enzymatic Probes Visualizing Sub-cellular/Deep-brain Caspase Activities in Neurodegenerative Models

*Linghui Qian, Cheng-Wu Zhang, Yanli Mao, Lin Li, Nengyue Gao, Kah-Leong Lim, Qing-Hua Xu, and Shao Q. Yao**

(*[*chmyaosq@nus.edu.sg](mailto:chmyaosq@nus.edu.sg)*)

List of Supplementary Videos

1. 3D videos

Introduction: 3D movies of immunofluorescence (IF) imaging for cleaved caspase-3 in STS (2 μM, 2 h) induced HeLa cells.

Description: (Red) cleaved caspase-3; (Green) MitoTracker; (Cyan) ER tracker.

Contents:

HeLa no STS: Merged 3D images of Mito channel (Green), ER channel (Cyan) and Casp-3 channel (Red) in HeLa cells without STS induction.

HeLa STS 2 h Mito-Casp3: Merged 3D images of Mito channel (Green) and Casp-3 channel (Red) in STS (2 μM, 2 h) induced HeLa cells (Fig. 3k).

HeLa STS 2 h Mito-ER: Merged 3D images of ER channel (Cyan) and Mito channel (Green) in STS (2 μM, 2 h) induced HeLa cells.

2. Real-time imaging

Introduction: real-time imaging (λ_{ex} = 405 nm; λ_{em} = 480-550 nm) of BV2 cells pretreated with probe for 1 h before addition of STS (2 μM) and images were taken thereafter for a period of 2.5 h.

Description: (Green) signal from released free dye (**AAN**).

Contents:

BV2 C3RA (2.5 h): BV2 cells were pretreated with **C3RA** (24 μM) for 1 h and STS was added afterwards. Then real-time imaging was carried out for a period of 2.5 h (Fig. 3e, up).

BV2 C8RA (2.5 h): BV2 cells were pretreated with **C8RA** (24 μM) for 1 h and STS was added afterwards. Then real-time imaging was carried out for a period of 2.5 h (Fig. 3e, down).

Supplementary Table 1. Photophysical and kinetic properties of probes and dyes.

Comp.	*N*-terminus	WH	*C*-terminus	λ[a]	ε/10[3][b]	Φ[c]	εΦ[d]	δΦ[e]	caspase	K_M (μM)		K_{cat} (s^{-1})		K_{cat}/K_M ($mM^{-1}·s^{-1}$)	
										AAN/DAN probe	Tetrapeptide-AMC	AAN/DAN probe	Tetrapeptide-AMC	AAN/DAN probe	Tetrapeptide-AMC
**AAN**	-	-	-	344/496	9.9	0.17	1683	37.0	-	-	-	-	-	-	-
**DAN**	-	-	-	330/462	24.1	0.33	7953	142.4	-	-	-	-	-	-	-
**C1FS**	DAN	Pral	DR	330/462	11.6	0.01	116	0.3	caspase-1	3.7 ± 0.3	-	0.25 ± 0.02	-	68.40 ± 0.62	-
**C1RS**	-	Pral	AAN	316/448	8.4	0.02	168	1.3		3.2 ± 0.4	-	0.27 ± 0.00	-	84.33 ± 11.09	-
**C1RA**	Acetyl	YVAD	AAN	316/448	8.6	0.04	344	6.9		82.9 ± 21.8	43.2 ± 5.3	0.22 ± 0.03	0.04 ± 0.01	2.67 ± 0.30	0.98± 0.01
**C1RB**	Acetyl	WEHD	AAN	316/448	18.5	0.01	185	2.3		1.2 ± 0.0	7.5 ± 1.9	0.63 ± 0.00	0.43 ± 0.01	516.04 ± 17.06	59.30 ± 16.05
**C3RA**	Acetyl	DEVD	AAN	316/448	11.1	0.03	333	2.9	caspase-3	6.8 ± 1.4	9.9 ± 2.3	1.82 ± 0.00	5.46 ± 0.00	277.92 ± 10	566.81 ± 15
**C3RM**	TPP	DEVD	AAN	317/449	4.2	0.08	488	1.5		7.4 ± 1.0	-	1.31 ± 0.07	-	184.73 ± 77	-
**C3RE**	GLIB	DEVD	AAN	317/449	4.6	0.06	401	2.3		6.6 ± 0.8	-	1.06 ± 0.04	-	166.55 ± 28	-
**C6RA**	Acetyl	VEID	AAN	316/448	18.5	0.05	925	1.8	caspase-6	94.2 ± 10.3	266.1 ± 41.9	0.93 ± 0.01	0.19 ± 0.01	9.96 ± 1.21	0.72 ± 0.14
**C8RA**	Acetyl	IETD	AAN	316/448	21.4	0.06	1284	2.4	caspase-8	4.2 ± 0.6	18.9 ± 7.5	0.88 ± 0.01	0.09 ± 0.02	212.88 ± 32.06	4.94 ± 0.61
**C9RA**	Acetyl	LEHD	AAN	316/448	20.4	0.04	816	3.2	caspase-9	38.4 ± 18.4	747.6 ± 6.7	0.01 ± 0.00	0.01 ± 0.00	0.24 ± 0.11	0.02 ± 0.00

[a] Peak position of the longest absorption/emission band. [b] Molar absorption coefficient (ε) at the absorption maximum of probe/fluorophore in $M^{-1}cm^{-1}$. [c] Quantum yields determined by using fluorescein aqueous NaOH (*p*H 13) as standard. [d] Brightness upon the absorption maximum of the compound. [e] The maxima two-photon action cross section values upon excitation wavelength of fluorophore from 760 to 850 nm in GM (1 GM = 10^{-50}•cm^4•s•$photon^{-1}$). -: not applicable.

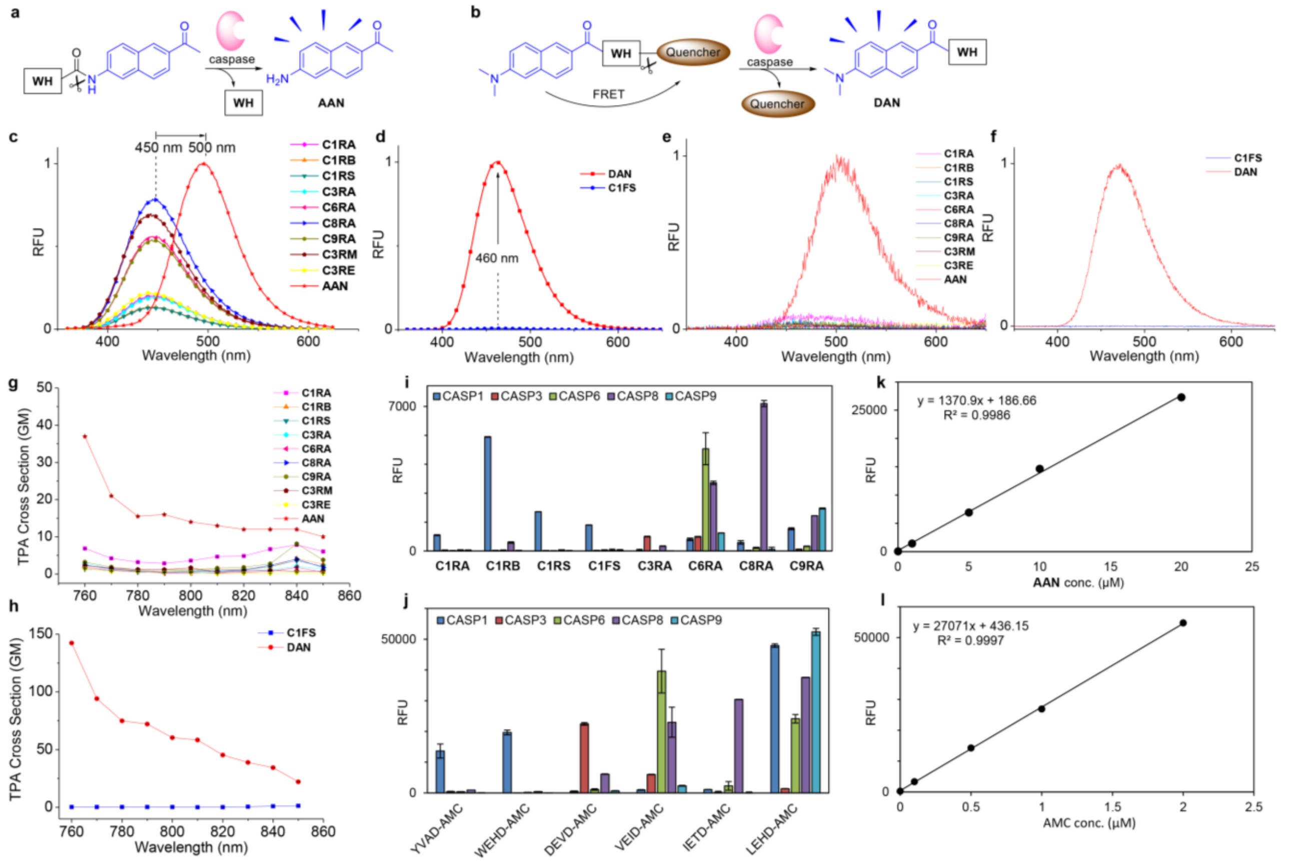

Supplementary Figure 1. Photophysical and enzymatic properties of probes/dyes. (**a**) The internal charge transfer (ICT) and (**b**) Förster Resonance Energy Transfer (FRET) strategies used in current study. Upon cleavage of the warhead (WH), strong fluorescence in the two-photon fluorophore was released, resulting in detection of caspase activity. (**c**-**d**) One-photon excited fluorescence spectra of 1 μM probes/dyes in HEPES buffer (*p*H = 7.2) excited at absorption maximum. (**e**-**f**) Representative two-photon excited fluorescence spectra of 1 μM probes/dyes at 760 nm. (**g**-**h**) Two-photon action (TPA) cross-section spectra of probes/dyes upon excitation wavelength from 760 to 850 nm determined by using **AAN** as a reference. (**i**-**l**) Probe selectivity profiles of our two-photon probes (**i**) with the corresponding commercial tetrapeptide-AMC(**j**) as references, together with free dye **AAN** (**k**) and AMC (**l**). For each probe (6 μM), uniform conditions with different caspases were applied. Fluorescence values were obtained after 2-h incubation at room temperature with $\lambda_{ex} = 360 \pm 40$ nm, $\lambda_{em} = 528 \pm 20$ nm, except for tetrapeptide-AMC and **C1FS** where $\lambda_{em} = 460 \pm 40$ nm.

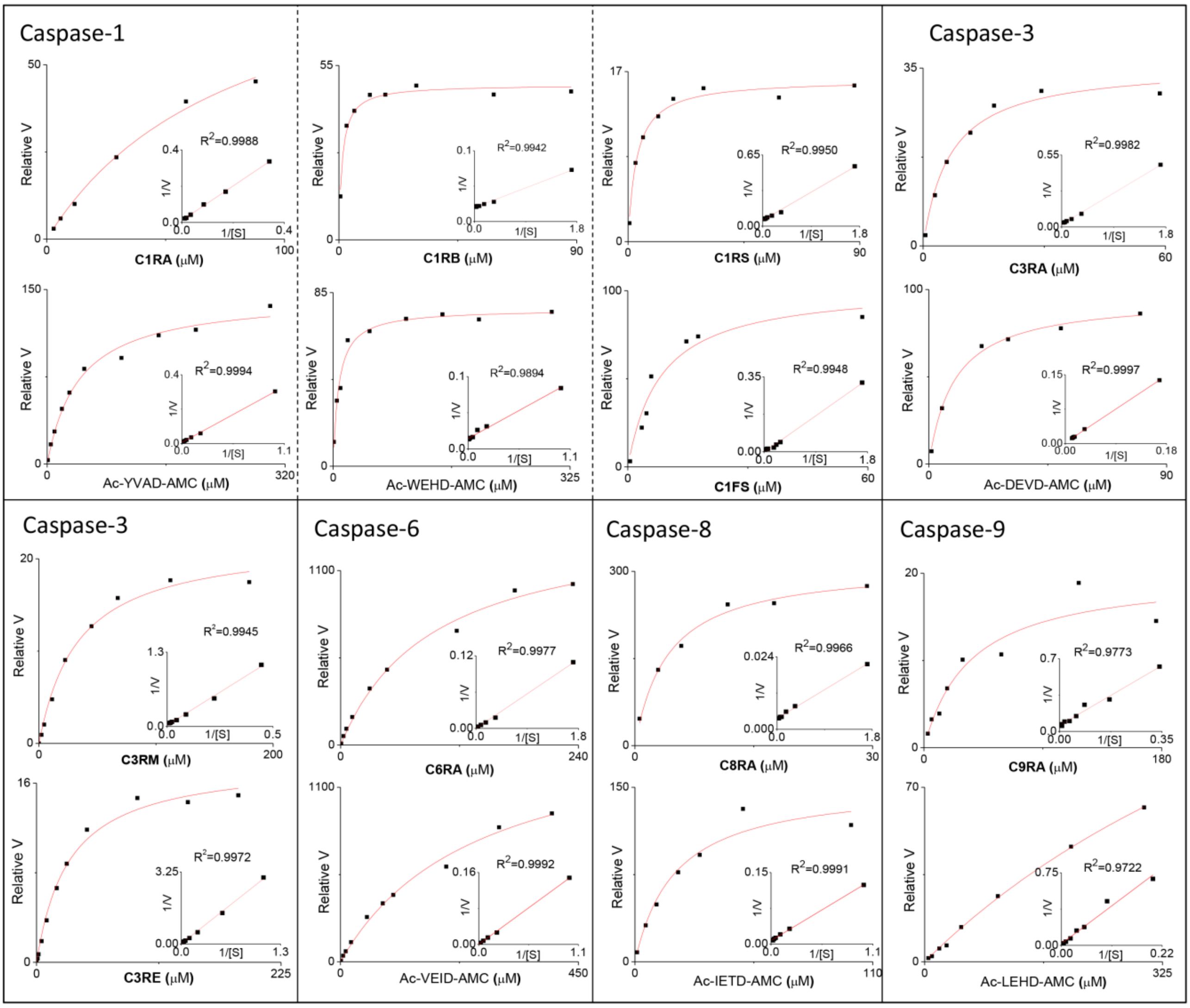

Supplementary Figure 2. Kinetic studies of all probes together with tetrapeptide-AMC as references in fitted Michaelis-Menton plot. Inset: fitted Lineweaver-Burk plot.

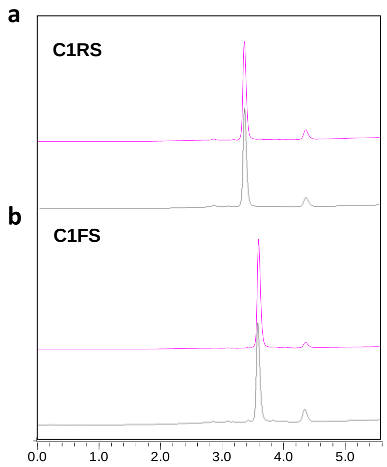

Supplementary Figure 3. Hydrolytic stability of 100 μM caspase-1 probes **C1RS** (**a**) and **C1FS** (**b**) after incubation in 2 μg/μL BV2 lysates for 24 h at room temperature. Pink: t = 0 h; Grey: t = 24 h.

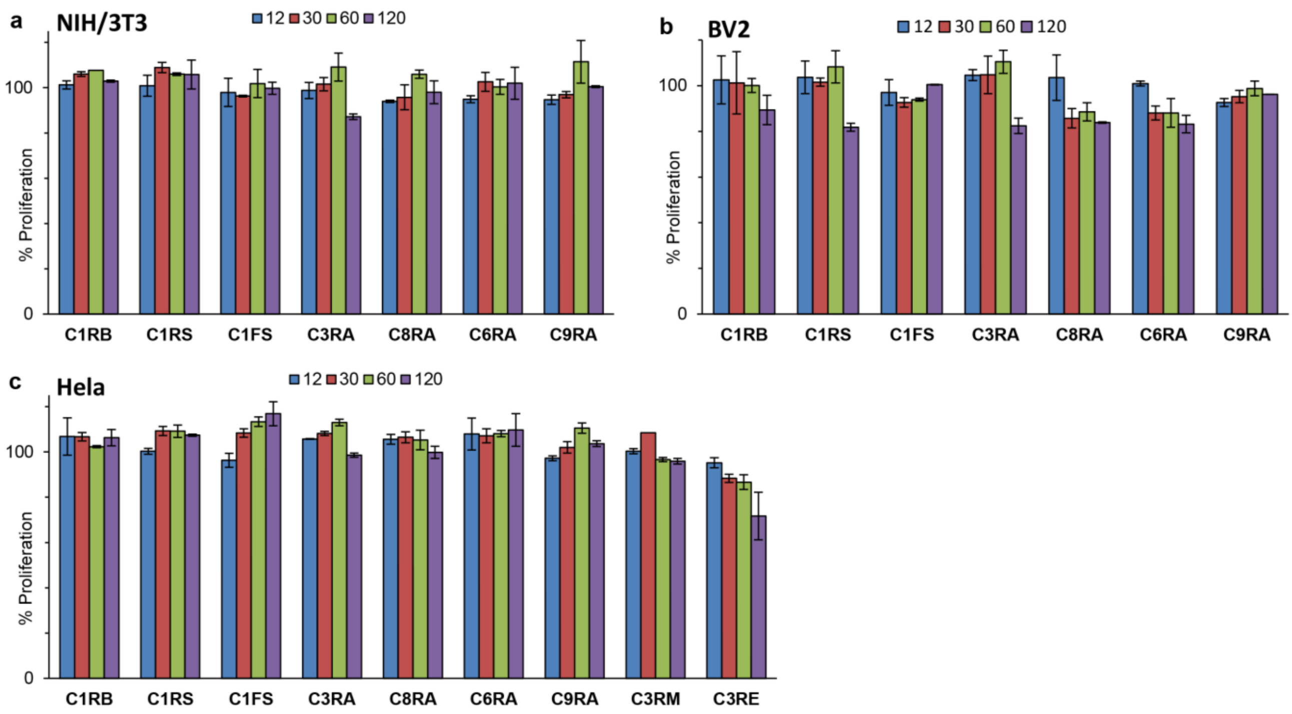

Supplementary Figure 4. Cytotoxicity of two-photon enzymatic probes (12, 30, 60, 120 μM) in mammalian cells (NIH/3T3, BV2 and HeLa) after incubation for 24 h, as determined by XTT assay.

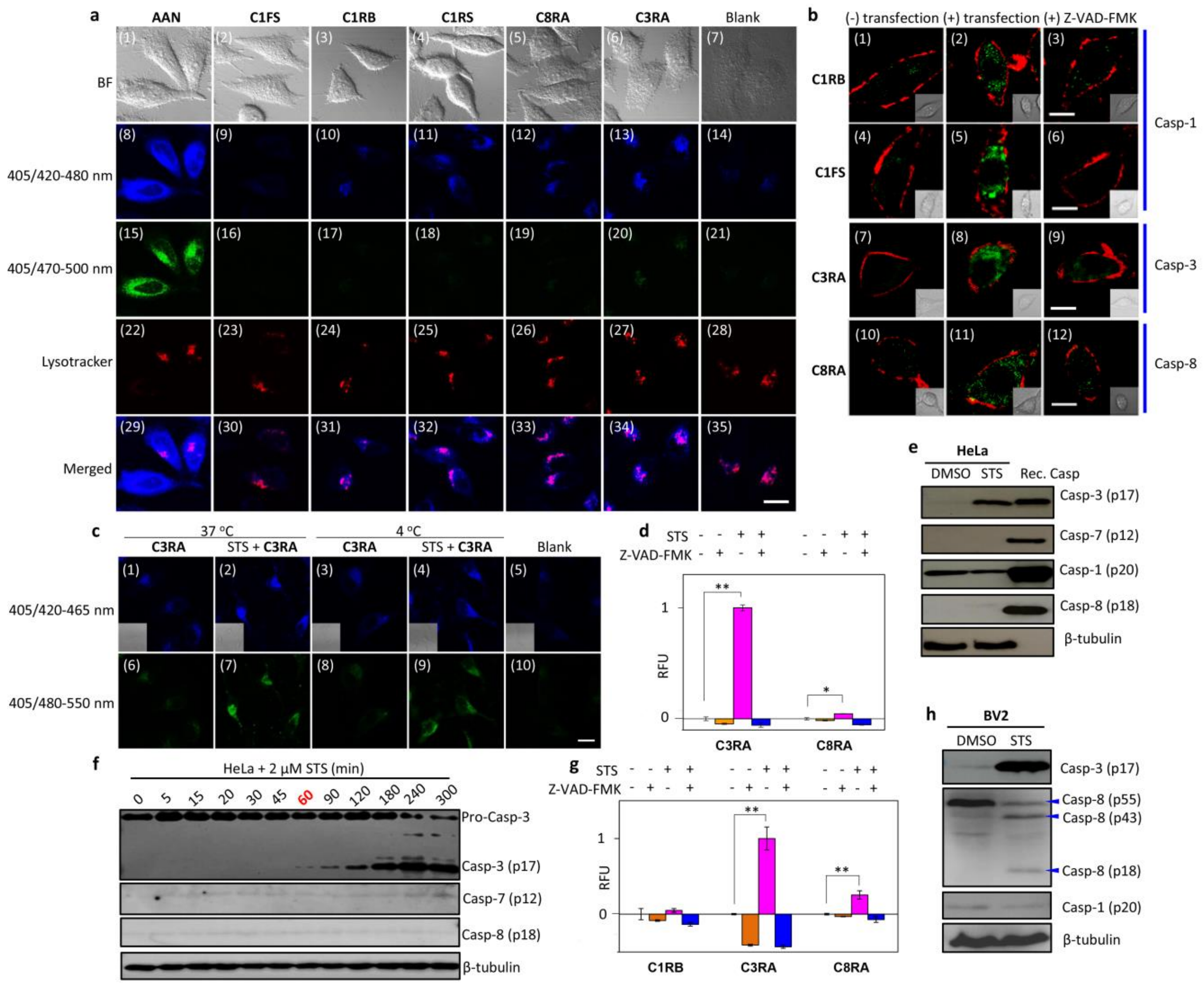

Supplementary Figure 5. Detection of caspase activities in live mammalian cells. (**a**) One-photon images of **AAN** (5 µM)/probes (24 µM)/DMSO (blank) in live HeLa cells after 1-h incubation together with LysoTracker. (Panels 1-7) bright field; (Panels 8-14) λ_{ex} = 405 nm, λ_{em} = 420-480 nm (indicative of probes, blue); (Panels 15-21) λ_{ex} = 405 nm, λ_{em} = 470-500 nm (indicative of **AAN**, green); (Panels 22-28) LysoTracker (λ_{ex} = 543 nm, λ_{em} = 560-615 nm, red); (Panels 29-35) merged images of blue/red channels. Scale bar: 20 µm. (**b**) Bioimaging of NIH/3T3 cells transfected with active caspase-1 (Panels 2/3/5/6; 50 ng)/caspase-3 (Panels 8/9; 15 ng)/caspase-8 (Panels 11/12; 5 ng) with/without inhibitor treatment (Panels 3/6/9/12, 50 µM of Z-VAD-FMK incubated for 1 h before probes were added) as well as control cells (Panels 1/4/7/10, no caspase transfection) by using **C1RB** (24 µM)/**C1FS** (12 µM) for caspase-1, **C3RA** (24 µM) for caspase-3 and **C8RA** (24 µM) for caspase-8. Cells were co-stained with membrane tracker. (Green) signals from caspase-cleaved probes (λ_{ex} = 760 nm, λ_{em} = 480-550 nm for all probes, except for **C1FS** with λ_{em} = 450-550 nm). (Red) membrane tracker (λ_{ex} = 650 nm, λ_{em} = 655-740 nm). (Inset) bright field. Scale bar = 10 µm. (**c**) One-photon (λ_{ex} = 405 nm) images of normal/apoptotic (1 µM STS pretreated for 4 h) HeLa cells imaged by **C3RA** (24 µM) at 37 ºC (Panels 1/2/6/7) or 4 ºC (Panels 3/4/8/9). (Inset) bright field. (Panels 1-5) λ_{em} = 420-465 nm (indicative of **C3RA)**. (Panels 6-10) λ_{em} = 480-500 nm (indicative of **AAN**). Scale bar = 20 µm. (**d**) Significantly increased caspase-3-like but not caspase-8-like activities detected with 6 µM of **C3RA**/**C8RA** in 30 µg apoptotic HeLa lysates (1 µM STS, 4 h) compared to normal Hela lysates (DMSO) (37 ºC, t = 2 h). For inhibition, lysates were incubated with Z-VAD-FMK (50 µM) for 3 h at 4 ºC before probe addition. (**e**) WB of normal/apoptotic HeLa cell lysates with anti-caspase-3/-7/-1/-8 antibodies. Recombinant human active caspase-

3/-7/-1/-8 (Rec. Casp) was used as positive control. (**f**) WB analysis of time-dependent caspase-3/-7/-8 activation by detection of pro-/cleaved caspase-3/-7/-8 from lysates of HeLa cells stimulated with STS (2 μM) over 5 h (reproduced in the maintext Fig. 3h). (**g**) Caspase activities examined with **C1RB/C3RA/C8RA** (6 μM, 37 ºC, *t* = 1 h) in normal and apoptotic BV2 lysates (25 μg) with/without pretreatment of inhibitor (50 μM Z-VAD-FMK for 3-h incubation at 4 ºC). (**h**) WB of lysates from normal (DMSO) and apoptotic (2 μM STS, 4 h) BV2 cells. For enzymatic assays in (**d**) & (**g**), λ_{ex} = 360 ± 40 nm, λ_{em} = 528 ± 20 nm; * $P < 0.05$, ** $P < 0.01$, n = 3, Student's t-test, two-tailed. In (**d**) and (**g**), each set of data were independently normalized to the STS (-)/Z-VAD-FMK (-) channel (set to 0) and STS (+)/Z-VAD-FMK (-) channel (set to 1) of the **C3RA** readouts. Negative readings from the inhibitor-treated samples indicated the presence of base-level caspase-like activities as the signal from mock controls was set as "0" in RFU quantification.

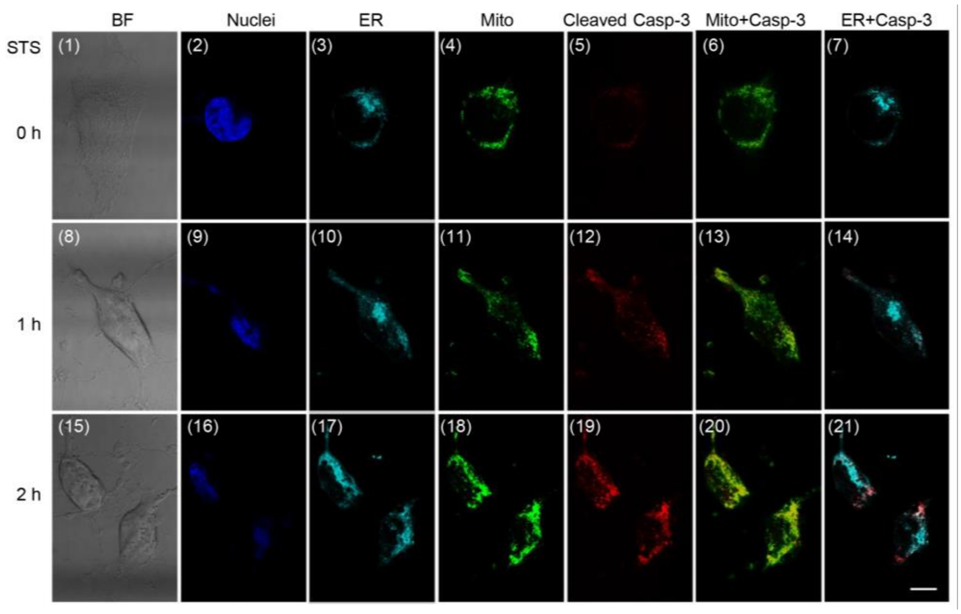

Supplementary Figure 6. 2D immunofluorescence imaging of active caspase-3 in HeLa cells upon STS (2.0 μM) induction for 0 h (Panels 1-7), 1 h (Panels 8-14) or 2 h (Panels 15-21). Cells were stained with anti-cleaved caspase-3 primary antibody and Cy5-conjugated anti-rabbit secondary antibody under Leica TCS SP5X Confocal Microscope. Cells were co-stained with Hoechst for nuclei (Panels 2/9/16; λ_{ex} = 405 nm, λ_{em} = 440-480 nm, Blue), ER tracker (Panels 3/10/17; λ_{ex} = 488 nm, λ_{em} = 500-550 nm, Cyan) and MitoTracker (Panels 4/11/18; λ_{ex} = 554 nm, λ_{em} = 570-650 nm, Green) to check the localization of active caspase-3. Panels 5/12/19: Cy5 for cleaved caspase-3, λ_{ex} = 630 nm, λ_{em} = 660-760 nm, Red; Panels 6/13/20: merged images of MitoTracker and Cy5; Panels 7/14/21: merged images of ER tracker and Cy5. Scale bar: 10 μm.

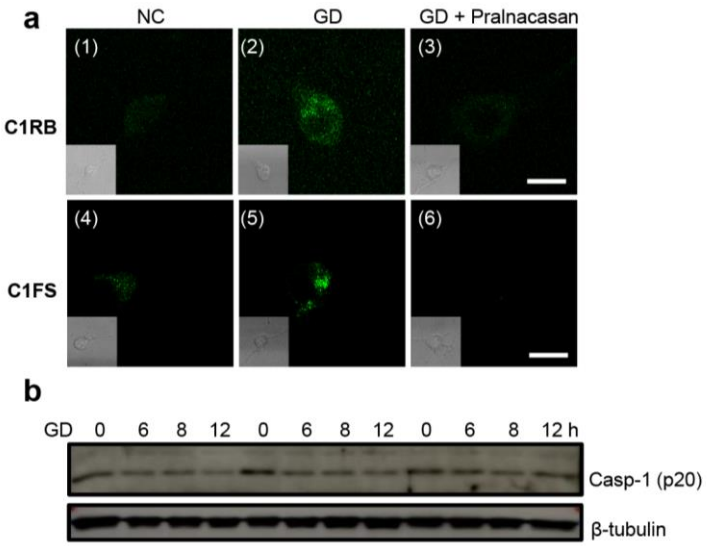

Supplementary Figure 7. Caspase-1-like activities detected in primary cortical neurons of C57BL/6 mouse underwent glucose-deprivation (GD) treatment. (**a**) Two-photon images indicated Pralnacasan-sensitive fluorescence signals in GD treated (8 h) primary neurons by using **C1RB** (24 μM, Panels 1-3) and **C1FS** (12 μM, Panels 4-6). Panels 1/4: primary neurons grown in normal culture medium and incubated with **C1RB**/**C1FS** for 1 h (NC). Panels 2/5: primary neuron underwent GD induction for 8 h and incubated with **C1RB**/**C1FS** for 1 h. Panels 3/6: during GD treatment, Pralnacasan (50 μM) was added 1 h earlier (t = 7 h) than **C1RB**/**C1FS**. Scale bar = 10 μm. (**b**) WB analysis of cleaved caspase-1 (p20) in primary neuron lysates after GD treatment for indicated time (0, 6, 8, 12 h).

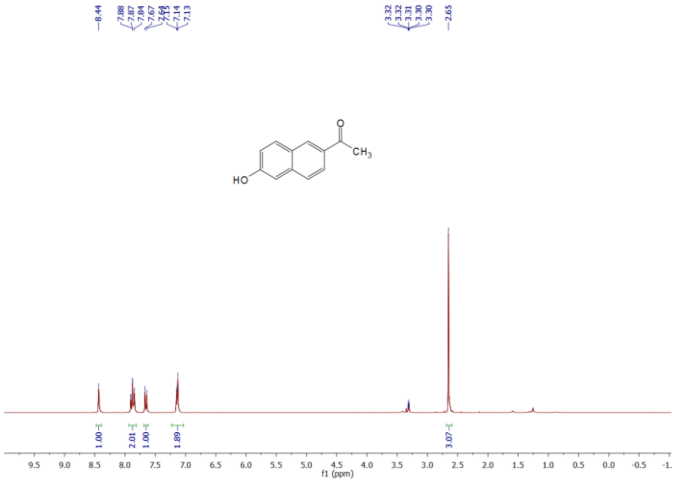

Supplementary Figure 8. ^{1}H-NMR spectra of **1.**

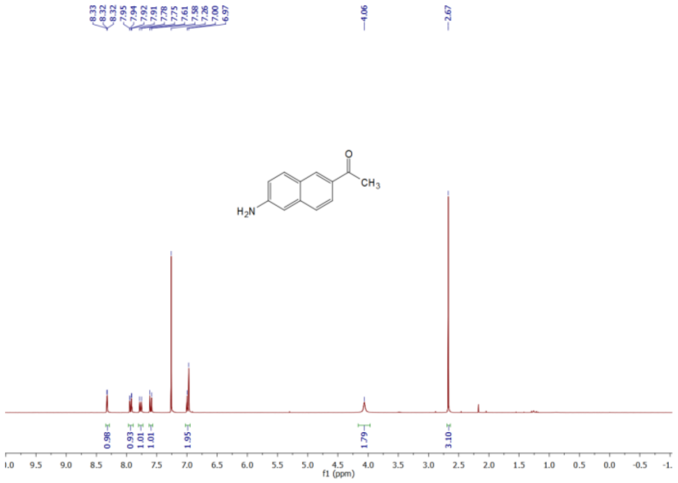

Supplementary Figure 9. ^{1}H-NMR spectra of **2.**

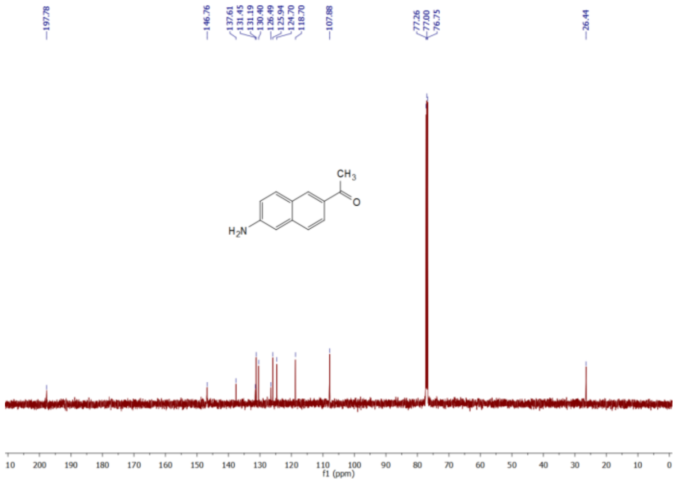

Supplementary Figure 10. ^{13}C-NMR spectra of **2.**

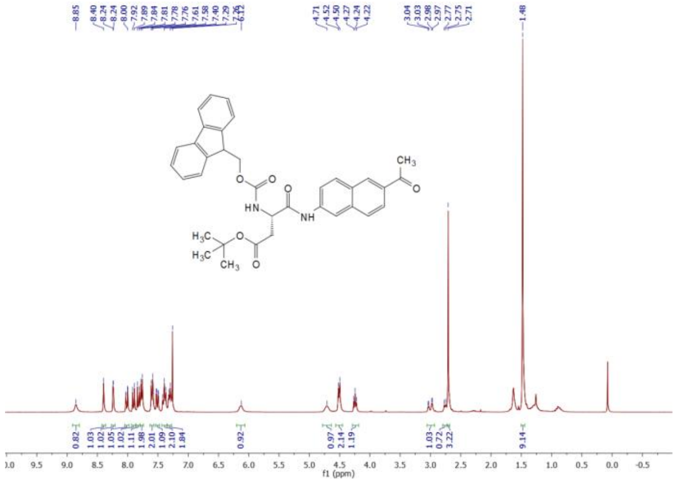

Supplementary Figure 11. ^{1}H-NMR spectra of **3a.**

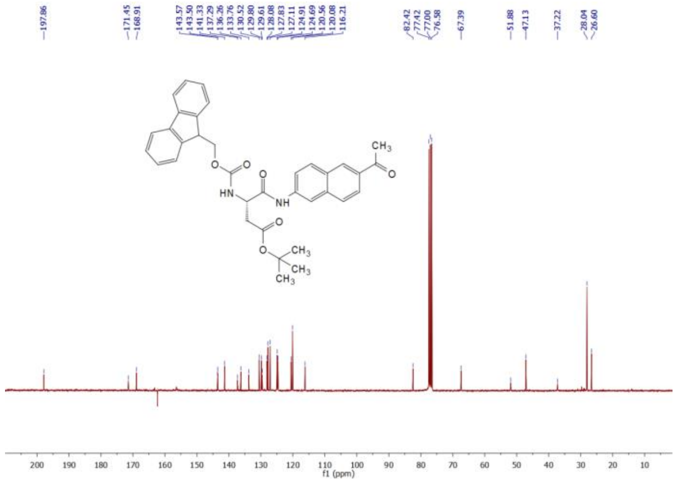

Supplementary Figure 12. ^{13}C-NMR spectra of **3a.**

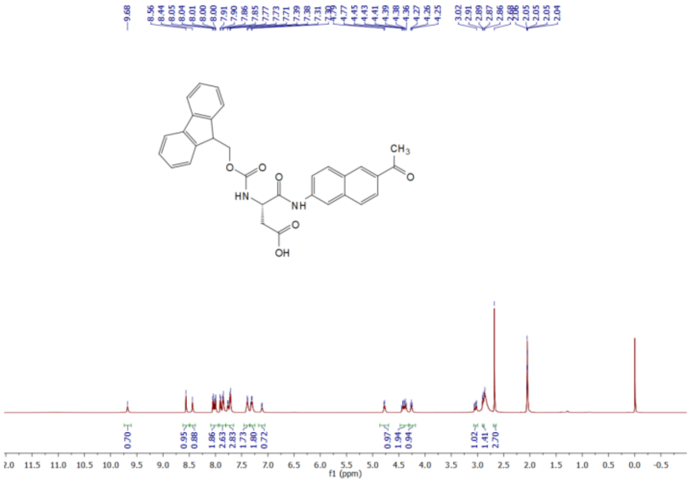

Supplementary Figure 13. ^{1}H-NMR spectra of **3b.**

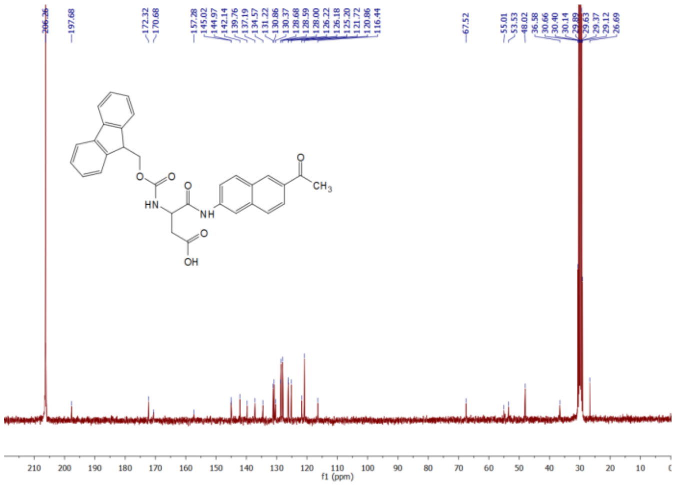

Supplementary Figure 14. ^{13}C-NMR spectra of **3b.**

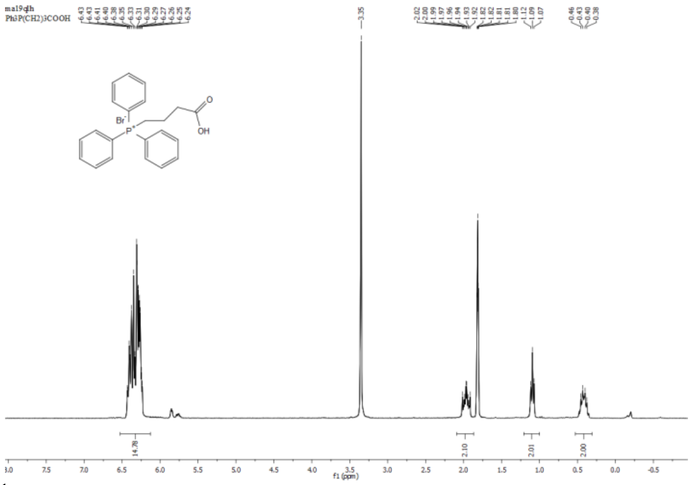

Supplementary Figure 15. ^{1}H-NMR spectra of **M1.**

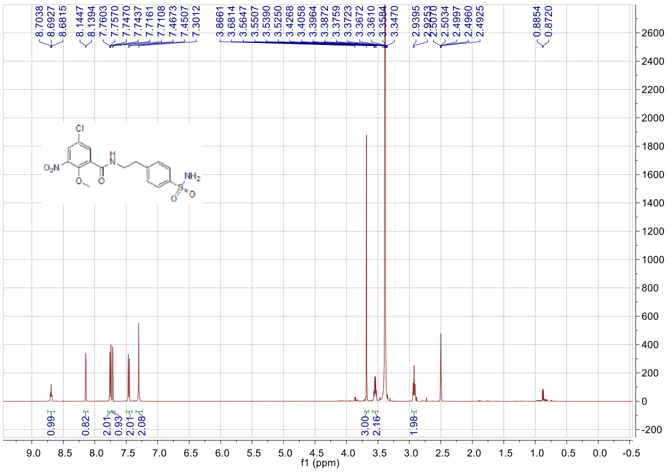

Supplementary Figure 16. ^{1}H-NMR spectra of **E4.**

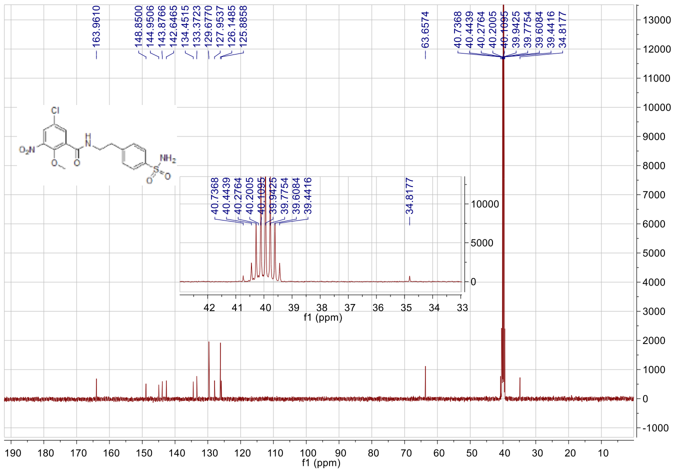

Supplementary Figure 17. ^{13}C-NMR spectra of **E4.**

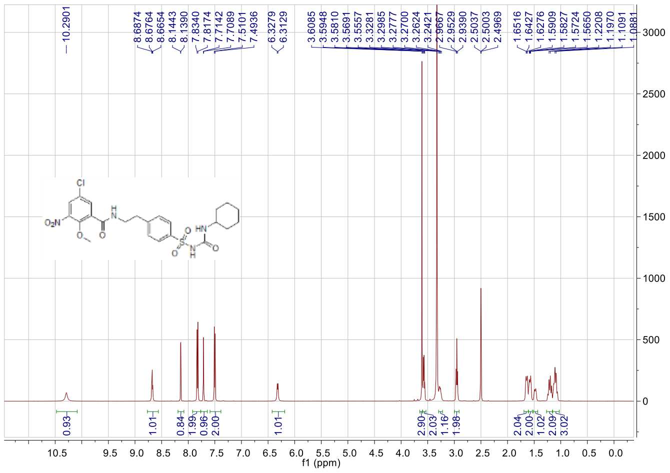

Supplementary Figure 18. ^{1}H-NMR spectra of **E5.**

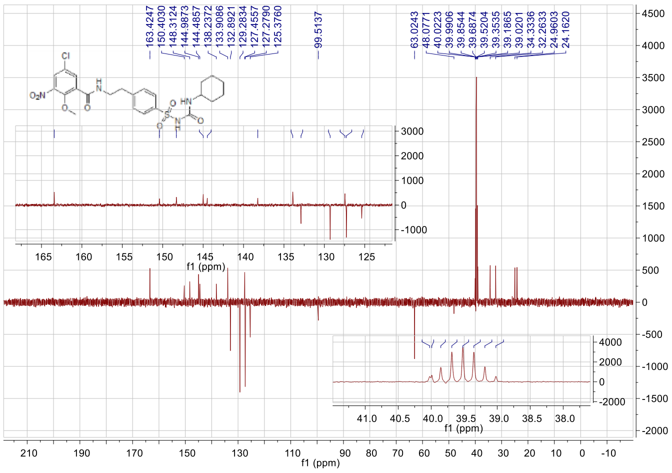

Supplementary Figure 19. ^{13}C-NMR spectra of **E5.**

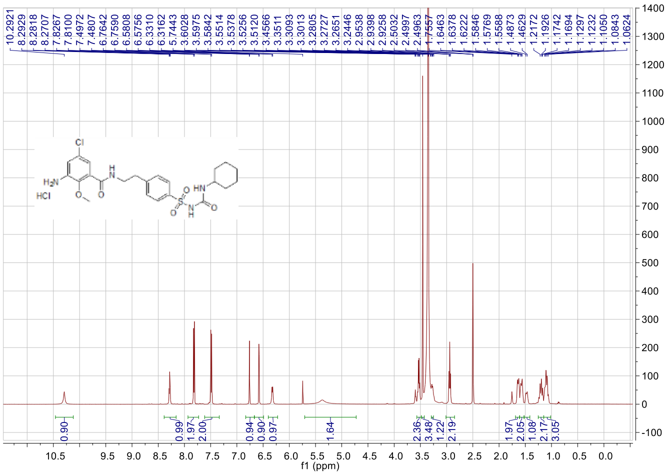

Supplementary Figure 20. ^{1}H-NMR spectra of **E6.**

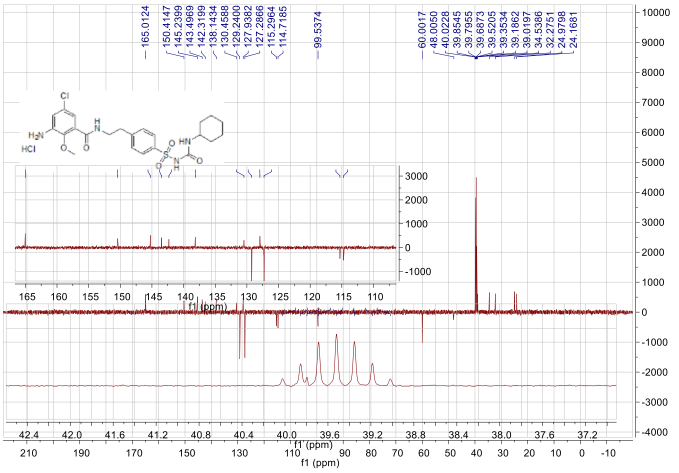

Supplementary Figure 21. ^{13}C-NMR spectra of **E6.**

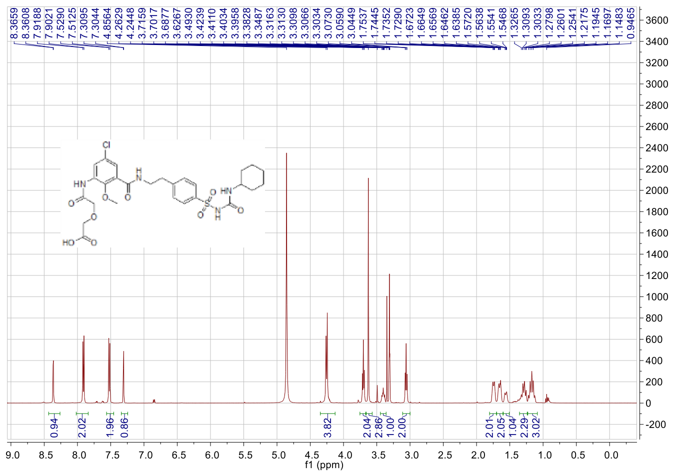

Supplementary Figure 22. ^{1}H-NMR spectra of **E7.**

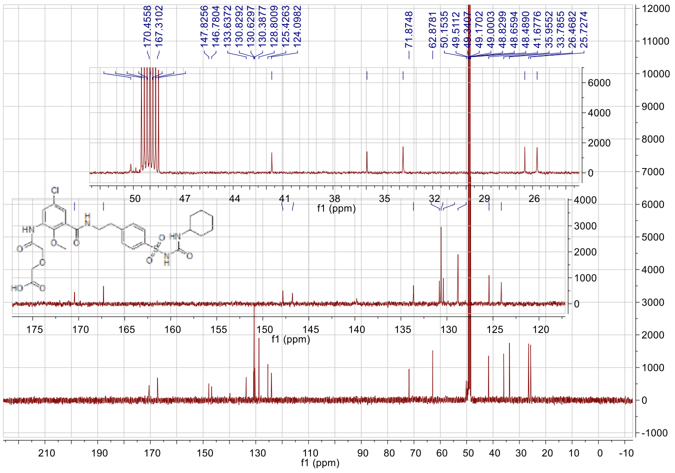

Supplementary Figure 23. ^{13}C-NMR spectra of **E7.**

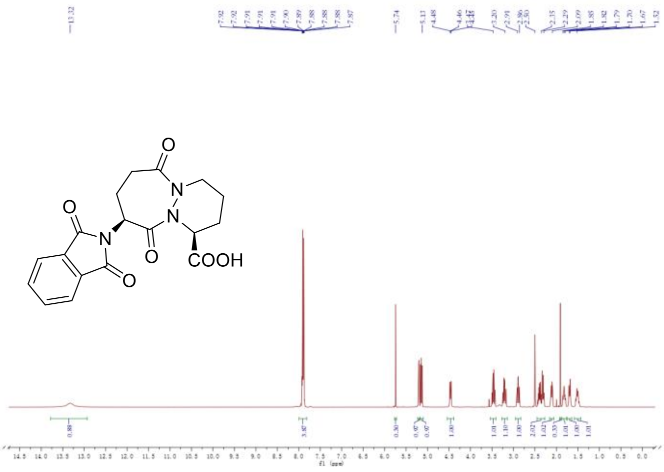

Supplementary Figure 24. ^{1}H-NMR spectra of **4a.**

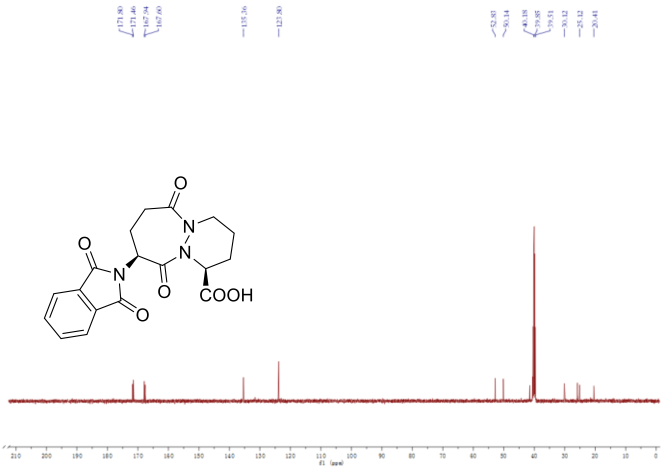

Supplementary Figure 25. ^{13}C-NMR spectra of **4a.**

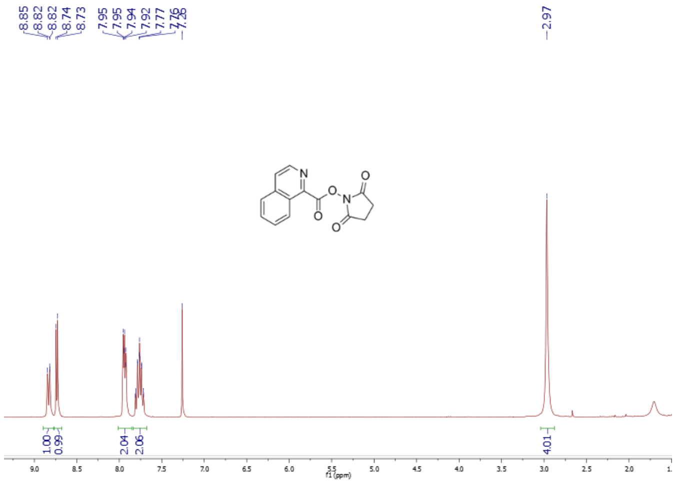

Supplementary Figure 26. ^{1}H-NMR spectra of **5.**

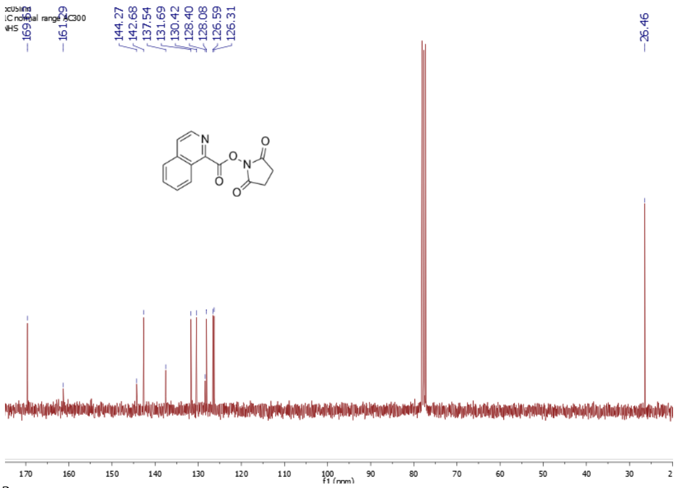

Supplementary Figure 27. ^{13}C-NMR spectra of **5.**

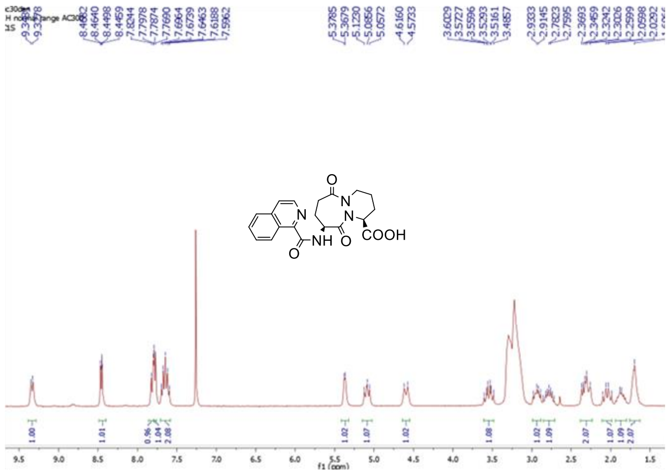

Supplementary Figure 28. ^{1}H-NMR spectra of **6.**

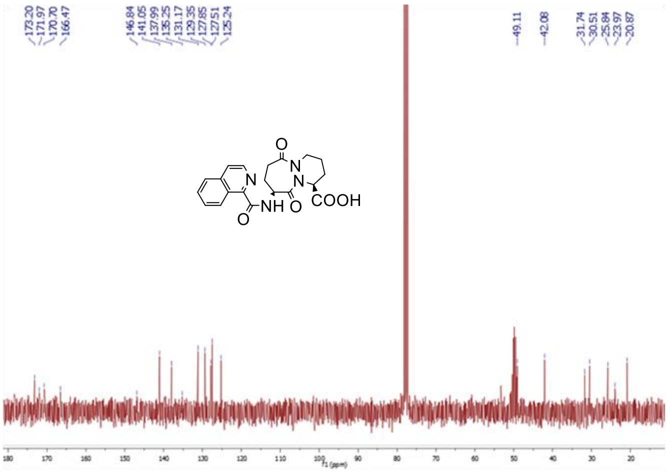

Supplementary Figure 29. ^{13}C-NMR spectra of **6.**

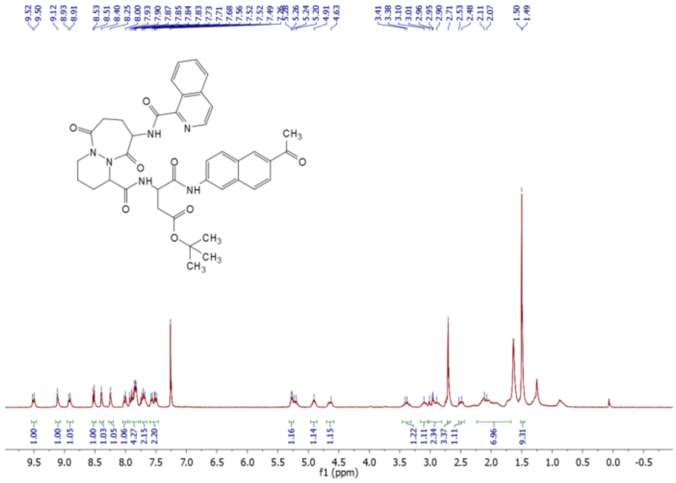

Supplementary Figure 30. ^{1}H-NMR spectra of **7.**

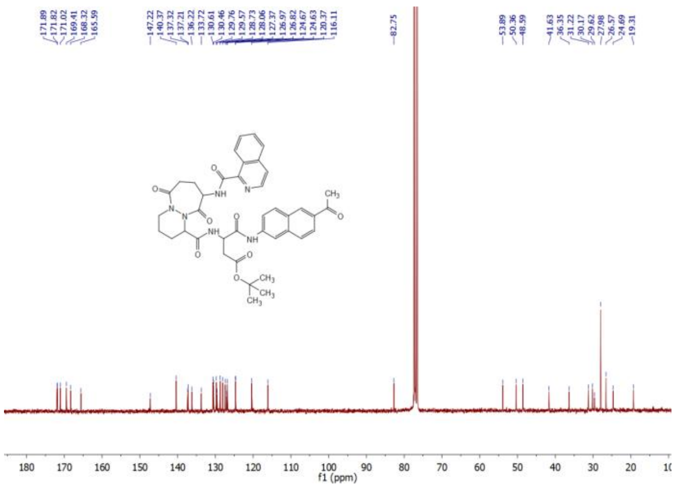

Supplementary Figure 31. ^{13}C-NMR spectra of **7.**

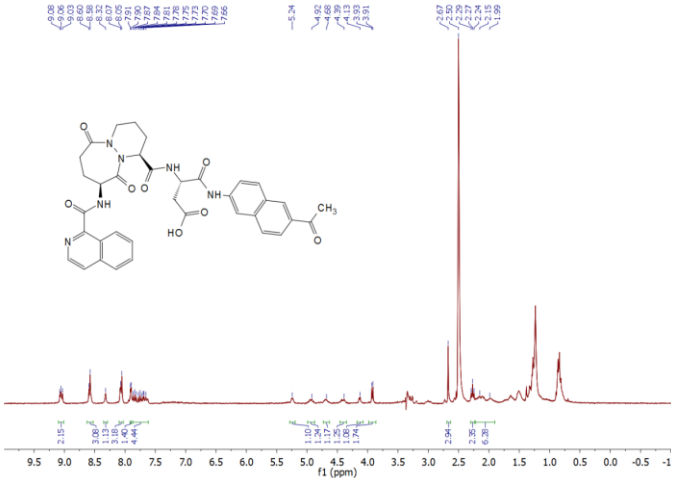

Supplementary Figure 32. ^{1}H-NMR spectra of **C1RS.**

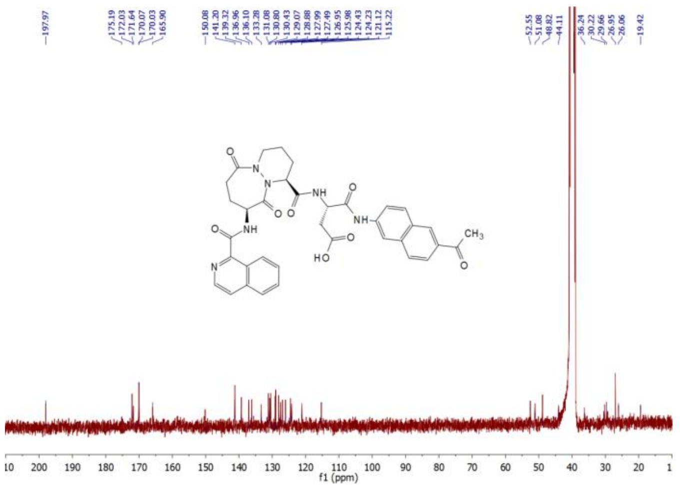

Supplementary Figure 33. ^{13}C-NMR spectra of **C1RS.**

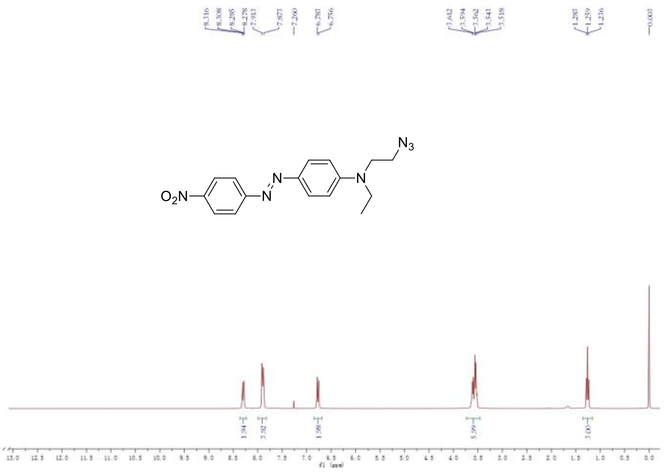

Supplementary Figure 34. ^{1}H-NMR spectra of **F1.**

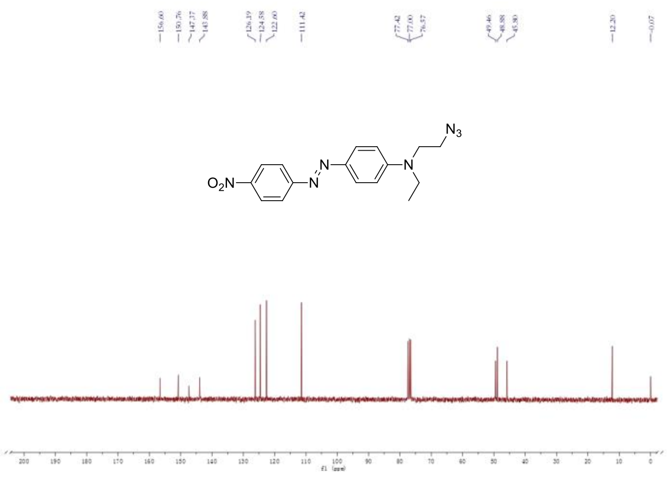

Supplementary Figure 35. ^{13}C-NMR spectra of **F1.**

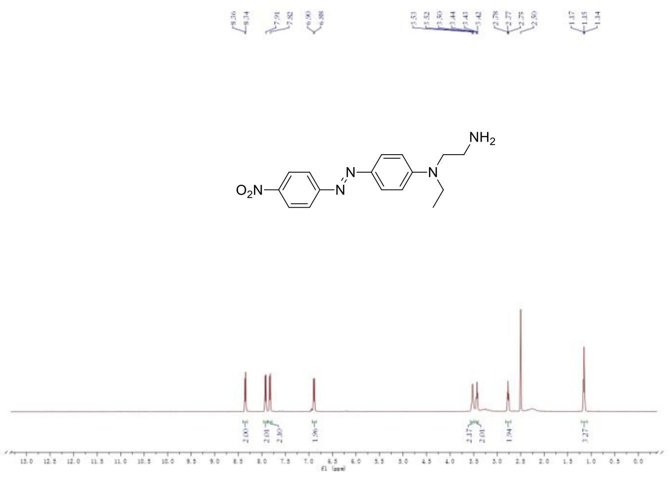

Supplementary Figure 36. ^{1}H-NMR spectra of **F2.**

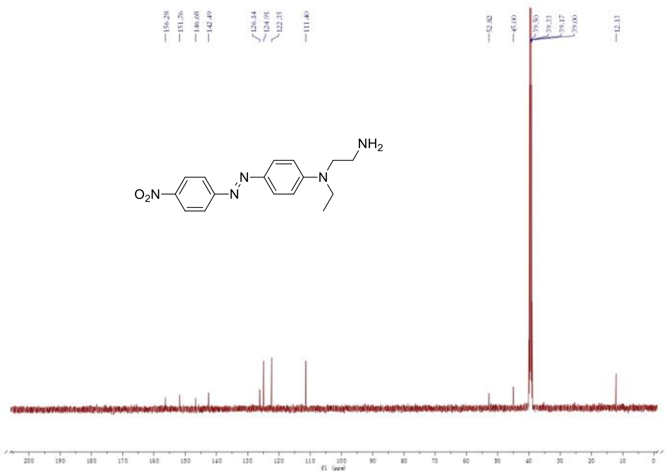

Supplementary Figure 37. ^{13}C-NMR spectra of **F2.**

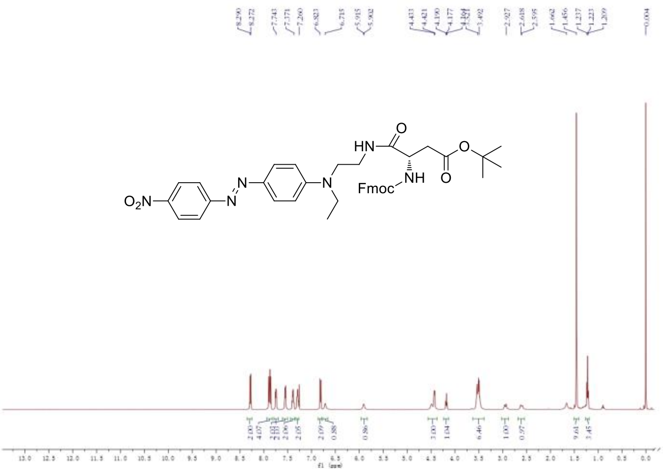

Supplementary Figure 38. ^{1}H-NMR spectra of **F3.**

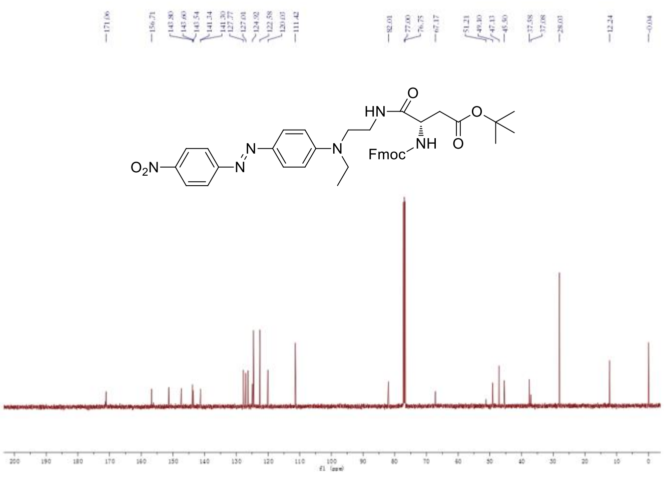

Supplementary Figure 39. ^{13}C-NMR spectra of **F3.**

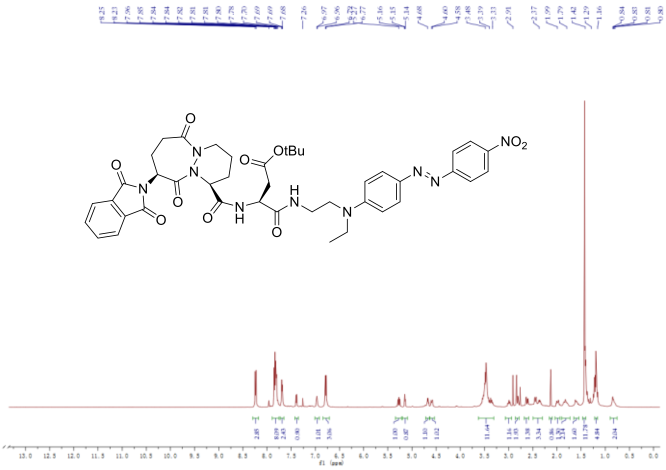

Supplementary Figure 40. ^{1}H-NMR spectra of **F4.**

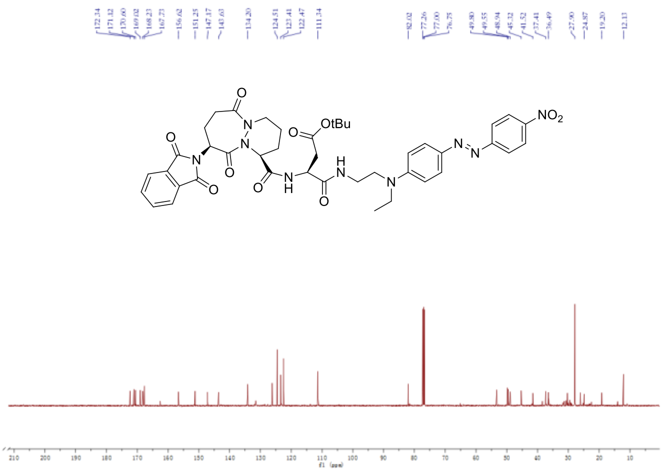

Supplementary Figure 41. ^{13}C-NMR spectra of **F4.**

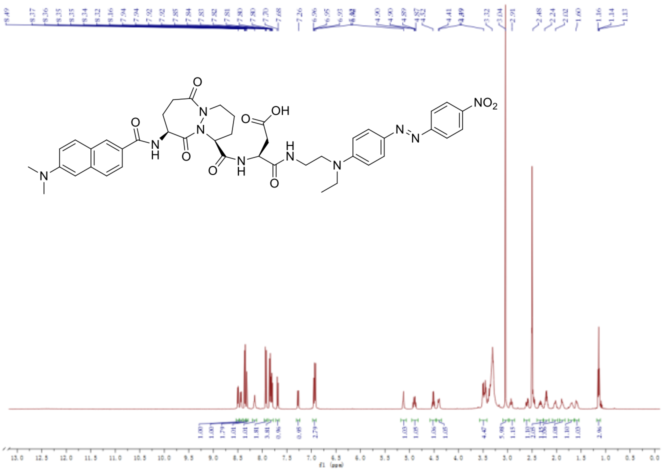

Supplementary Figure 42. ^{1}H-NMR spectra of **C1FS.**

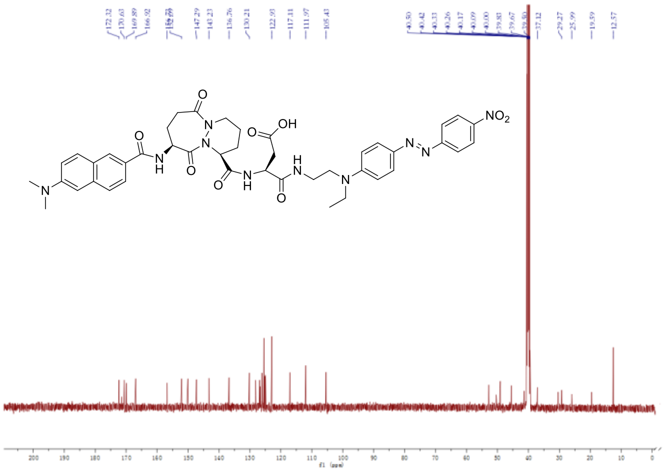

Supplementary Figure 43. ^{13}C-NMR spectra of **C1FS.**

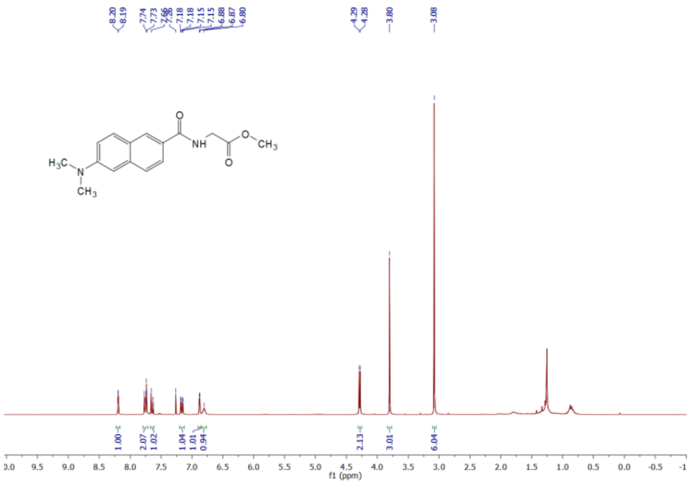

Supplementary Figure 44. ^{1}H-NMR spectra of **DAN.**

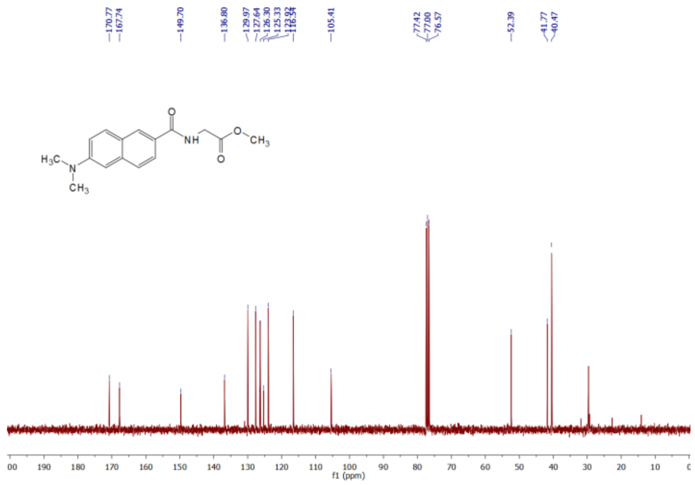

Supplementary Figure 45. ^{13}C-NMR spectra of **DAN.**

(C1RA)

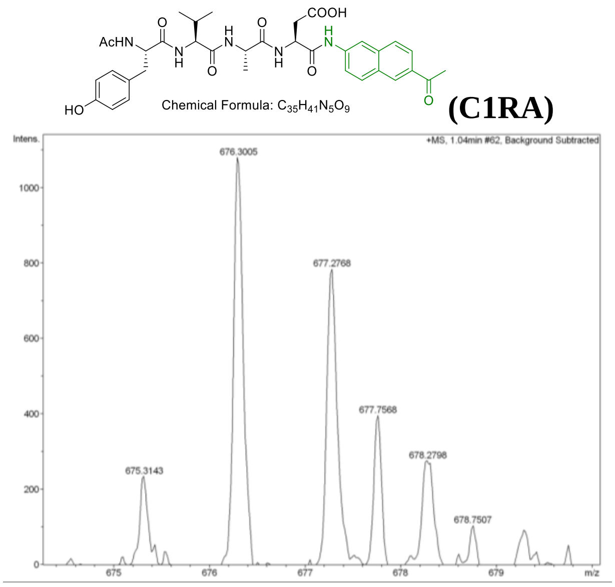

Supplementary Figure 46. ESI-HRMS profile of **C1RA**. ESI-HRMS: *m/z* $[M+H]^+$ calcd, 676.2977; found, 676.3005.

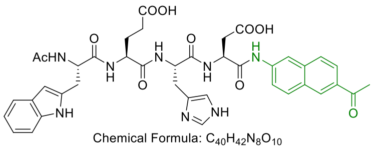

(C1RB)

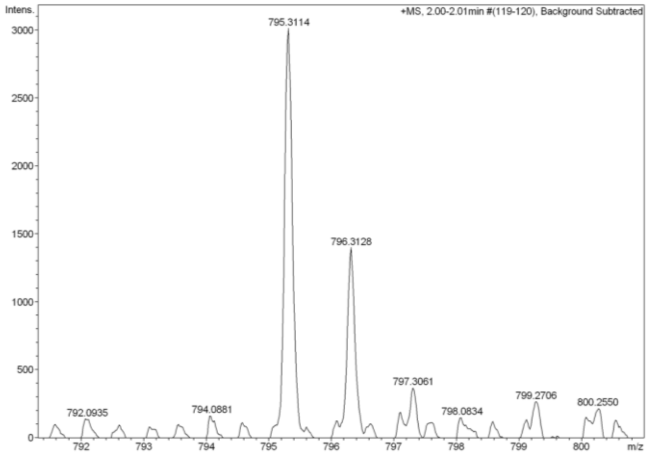

Supplementary Figure 47. ESI-HRMS profile of **C1RB**. ESI-HRMS: *m/z* $[M+H]^+$ calcd, 795.3097; found, 795.3114.

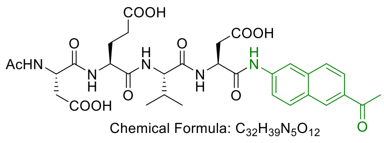

(C3RA)

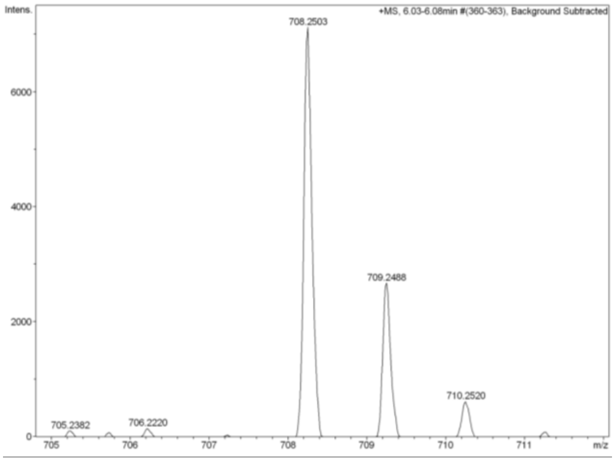

Supplementary Figure 48. ESI-HRMS profile of **C3RA**. ESI-HRMS: m/z $[M+Na]^+$ calcd, 708.2487; found, 708.2503.

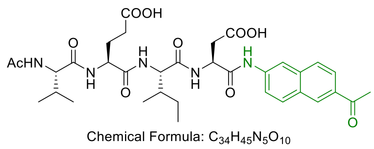

(C6RA)

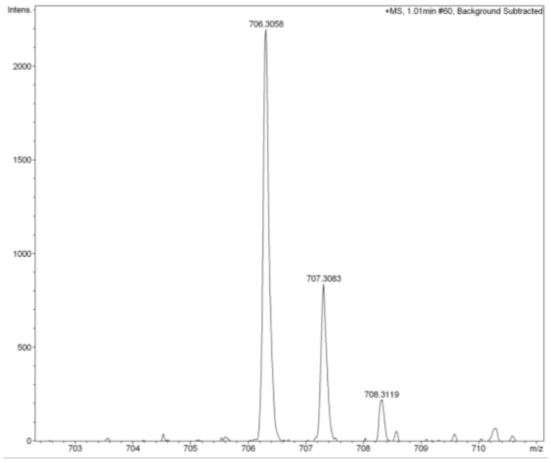

Supplementary Figure 49. ESI-HRMS profile of **C6RA**. ESI-HRMS: m/z $[M+Na]^+$ calcd, 706.3059; found, 706.3058.

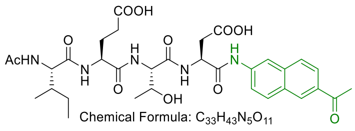

(C8RA)

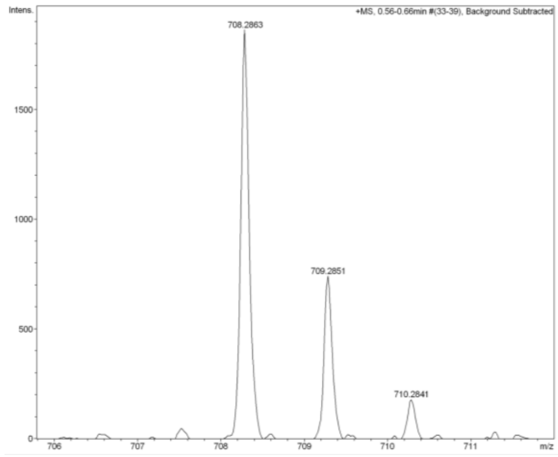

Supplementary Figure 50. ESI-HRMS profile of **C8RA**. ESI-HRMS: m/z $[M+Na]^+$ calcd, 708.28519; found, 708.2863.

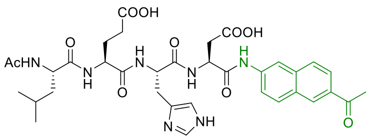

Chemical Formula: $C_{35}H_{43}N_7O_{10}$

(C9RA)

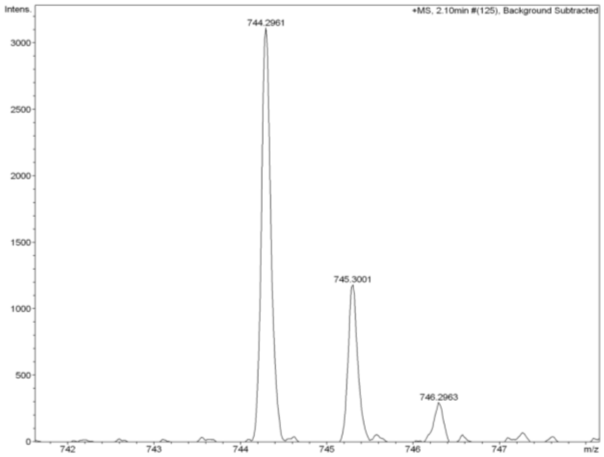

Supplementary Figure 51. ESI-HRMS profile of **C9RA**. ESI-HRMS: m/z $[M+Na]^+$ calcd, 744.2964; found, 744.2961.

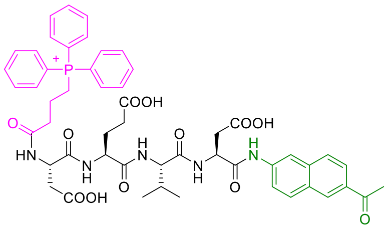

(C3RM)

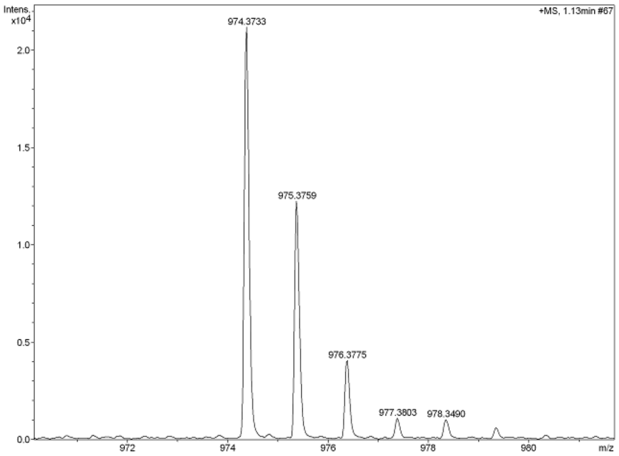

Supplementary Figure 52. ESI-HRMS profile of **C3RM**. ESI-HRMS: m/z $[M]^+$ calcd, 974.3736; found, 974.3733.

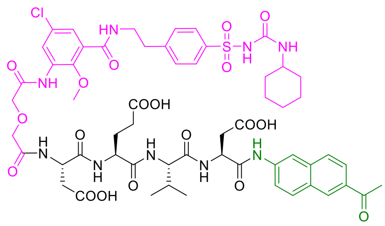

(C3RE)

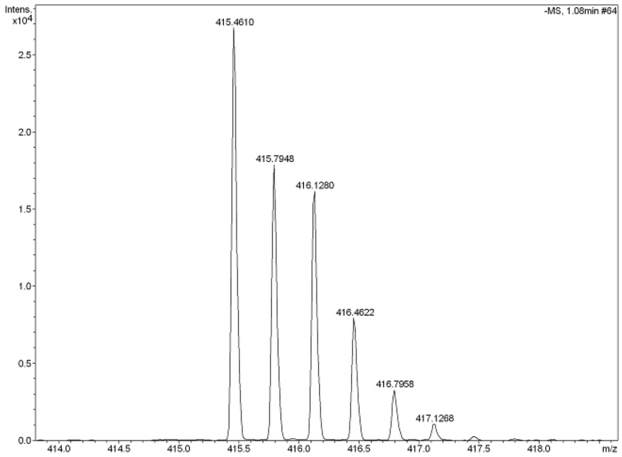

Supplementary Figure 53. ESI-HRMS profile of **C3RE**. ESI-HRMS: m/z $[M-3H]^{3-}$ calcd, 415.4607; found, 415.4610.

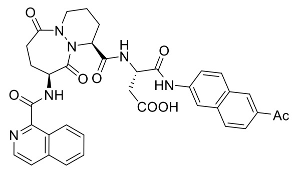

(C1RS)

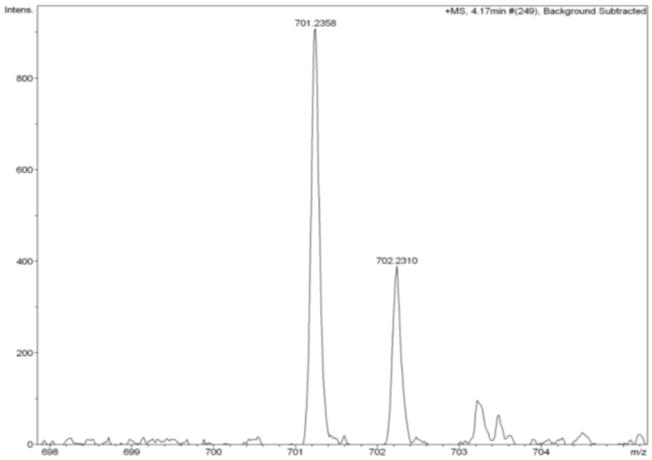

Supplementary Figure 54. ESI-HRMS profile of **C1RS**. ESI-HRMS: *m/z* $[M+Na]^+$ calcd, 701.2330; found, 701.2358.

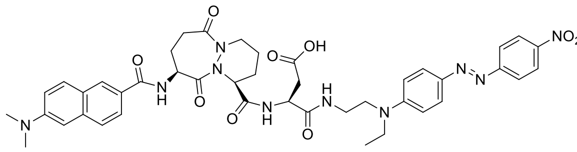

(C1FS)

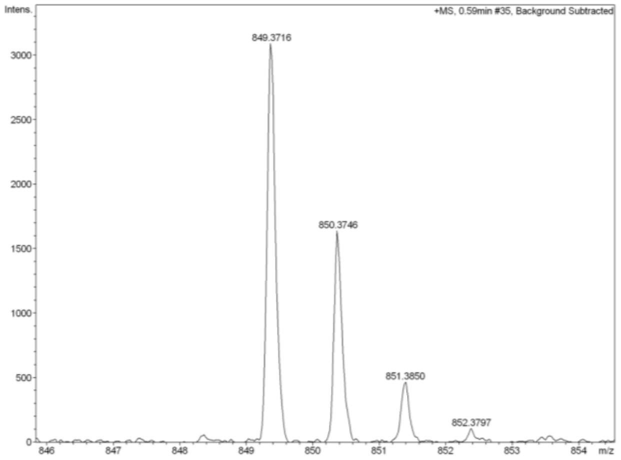

Supplementary Figure 55. ESI-HRMS profile of **C1FS**. ESI-HRMS: *m/z* $[M+H]^+$ calcd, 849.3678; found, 849.3716.

Supplementary Methods

All chemicals were purchased from commercial vendors and used without further purification, unless otherwise noted. All non-aqueous reactions were carried out under a nitrogen/argon atmosphere in oven-dried glassware. Reaction progress was monitored by TLC on pre-coated silica plates (Merck 60 $F_{254\,nm}$, 250 µm thickness) and spots were visualized by UV light or appropriate staining (e.g., ceric ammonium molybdate (CAM), basic $KMnO_4$). Flash column chromatography was carried out using silica gel (Merck 60 $F_{254\,nm}$ 0.040-0.063µm). All ^{1}H NMR and ^{13}C NMR spectra were taken on a Bruker AFC 300 (300 MHz) or AMX500 (500 MHz). Chemical shifts were reported in parts per million (ppm) relative to residual solvent peaks ($CDCl_3$ = 7.26 ppm, DMSO-d_6 = 2.50 ppm, CD_3OD = 3.31 ppm, Acetone-d_6 = 2.05 ppm). ^{1}H NMR coupling constants (*J*) are reported in Hertz (Hz) and multiplicity is indicated as follows: s (singlet), d (doublet), t (triplet), q (quartet), m (multiplet), br s (broad singlet), br d (broad doublet), dd (doublet of doublet), dt (doublet of triplet), dq (doublet of quartet), tq (triplet of quartet). Analytical HPLC and Mass spectra were recorded on a Shimadzu LC-IT-TOF spectrometer or LC-ESI spectrometer equipped with an autosampler, using reverse-phase Phenomenex Luna 5 µm C_{18} 100 Å 50 × 3.0 mm columns. Preparative HPLC was carried out on Gilson preparative HPLC system using Trilution software and reverse-phase Phenomenex Luna 5 µm $C_{18(2)}$ 100 Å 50 × 30.00 mm column. 0.1% TFA/H_2O and 0.1% TFA/acetonitrile were used as eluent for all HPLC experiments.

The flow rate was 0.6 mL/min for analytical HPLC and 10 mL/min for preparative HPLC. UV-vis absorption and fluorescence spectra were measured by using a Shimadzu UV 2550 spectrometer and a Perkin Elmer LS50 spectrofluorometer, respectively. The two-photon excited fluorescence measurements were performed using a Spectra Physics femtosecond Ti:sapphire oscillator (Tsunami) as the excitation source. The output laser pulses have a tunable center wavelength from 760 nm to 850 nm with pulse duration of 40 fs and a repetition rate of 76 MHz. The laser beam was focused onto the sample that was contained in a cuvette with path length of 1 cm. The emission from the sample was collected at 90 ° angle by a pair of lenses and an optical fiber that was connected to a monochromator (Acton, Spectra Pro 2300i) coupled with CCD system (Princeton Instruments, Pixis 100B). A short pass filter with cut-off wavelength at 700 nm was placed before the spectrometer to minimize the scattering from the pump beam. All optical measurements were performed at room temperature. All 2D images were acquired either on Carl Zeiss LSM 510 Meta Confocal Microscope equipped with Argon multi-line gas laser (458, 477, 488, 514 nm, 30.0 mW), Helium Neon (HeNe) gas laser (543 nm, 1.2mW), HeNe gas laser (633 nm, 5.0 mW) and Diode laser (405 nm, 30.0 mW) for one-photon excitation or Leica TCS SP5X Confocal Microscope System equipped with Leica HCX PL APO 63x/1.20 W CORR CS, 405 nm Diode laser, Argon ion laser, white laser (470 nm to 670 nm, with 1 nm increments, with 8 channels AOTF for simultaneous control of 8 laser lines, each excitation wavelength provides 1.5 mV), and Ti-Sapphire laser (~4 W at 800 nm) which corresponded to approximately 1% (~40 mW at 800 nm) average power in the focal plane for both one- and two-photon excitation. A PMT detector ranging from 420 nm to 700 nm for steady-state fluorescence was used with Leica and images were processed with Leica Application Suite Advanced Fluorescence (LAS AF). 3D images were acquired from PerkinElmer Ultraview Vox Spinning Disc Confocal with Olympus IX81 Microscope equipped with a 60 × water immersion objective and processed with Volocity 6.3.1 software.

Human recombinant caspases and caspase-3/7 inhibitor I (Calbiochem #218826) were obtained from Merck. Tetrapeptide-AMC caspase substrates were purchased from Enzo Life Sciences. The apoptosis inducer, staurosporine (STS) was bought from LC Laboratories. Anti-β-tubulin (ab6046), anti-caspase-1 (ab189796, human and mouse) antibodies and the broad-spectrum caspase inhibitor Z-VAD(OMe)-FMK (ab120487) were purchased from Abcam. Anti-cleaved caspase-3 (CST #9664, human and mouse) and anti-caspase 8 (CST #9746, human) antibodies were purchased from Cell

Signaling. Anti-caspase-7 antibody (sc-6138) was bought from Santa Cruz. Anti-caspase-3 (both full length and p17 of caspase-3, CST #9662, human and mouse) and anti-caspase-8 (ALX-804-447, mouse) antibodies were gifts from Prof. Han-Ming Shen (NUS).

Chemical Synthesis and Characterizations

The synthesis of all ten probes is summarized in Fig. 1, Supplementary Schemes 1 & 2. Briefly, the eight peptide-based **AAN** probes were conveniently prepared by solid-phase peptide synthesis (SPPS) from the AAN-coupled Fmoc-Asp-OH, **3b** (prepared from deprotection of *t*Bu-protected **3a** in Supplementary Scheme 1), with 2-chlorotrityl chloride (2-Cl-Tr-Cl) resin, by following standard Fmoc chemistry. For **C3RM** and **C3RE**, instead of capping the resin-bound tetrapeptide with acetic anhydride, (3-carboxypropyl)triphenylphosphonium bromide and an NHS ester of glibenclamide were used, respectively (Supplementary Scheme 1). For the synthesis of **C1RS** and **C1FS**, the key intermediate **4a** was first obtained by following published protocols.[1] Subsequently, **C1RS** was synthesized by first deprotection of **4a** (giving **4b**), followed by coupling with **5** to afford the core moiety of Pralnacasan, **6**. Further coupling with **3c** (obtained from Fmoc deprotection of **3a**) gave **7**, which upon removal of the *t*Bu group, gave **C1RS**. To synthesize **C1FS**, disperse red-1 (DR) was chemically modified with an amino group to give **F3**, which was subsequently coupled to Fmoc-Asp(O*t*Bu)-OH, providing **F4**. Upon Fmoc-deprotection and coupling of **4a** (giving **F5**), followed by hydrazinolysis, the resulting compound was coupled with **DAN**, followed by TFA deprotection to give **C1FS**.

a solution-phase synthesis

i. Two-photon fluorophore

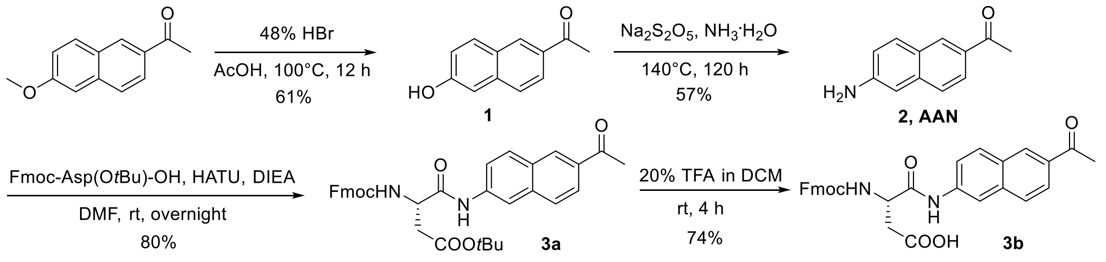

ii. Mitochondria-targeting moiety (TPP)

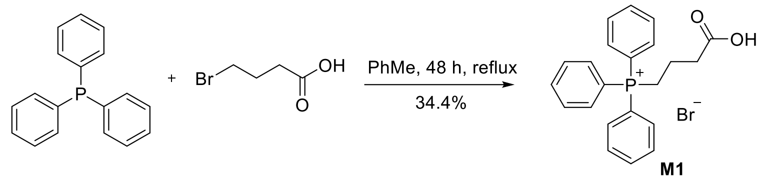

iii. Endoplasmic reticulum-targeting moiety (GLIB)

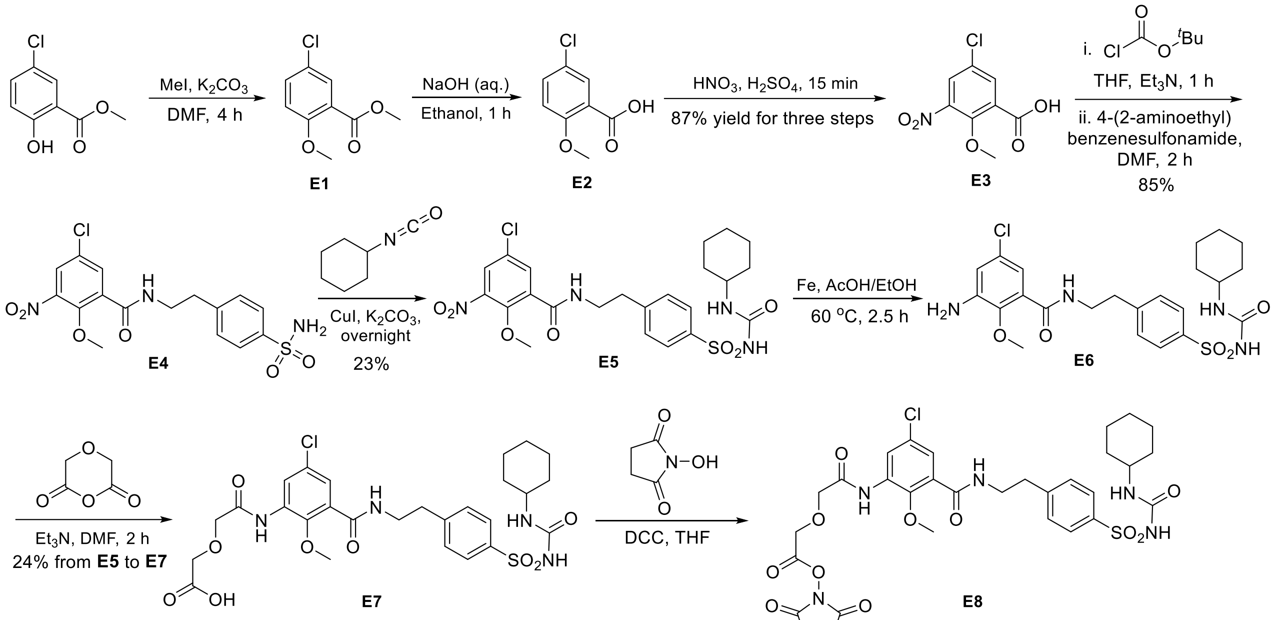

b Solid-phase peptide synthesis (SPPS)

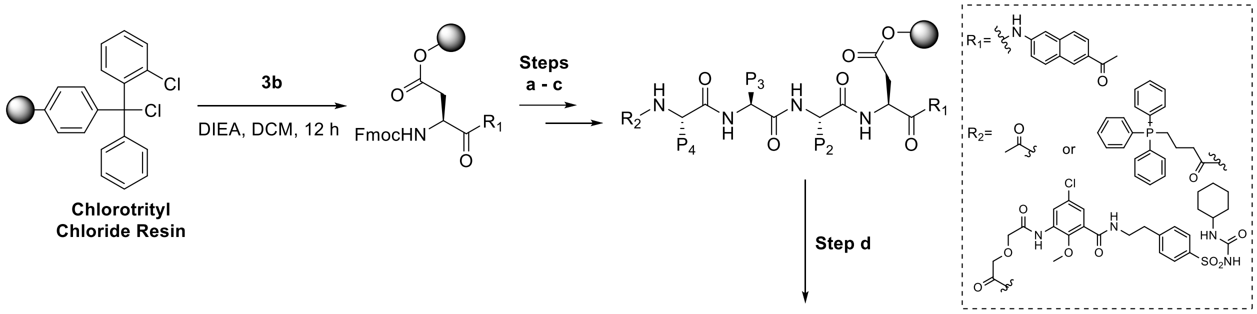

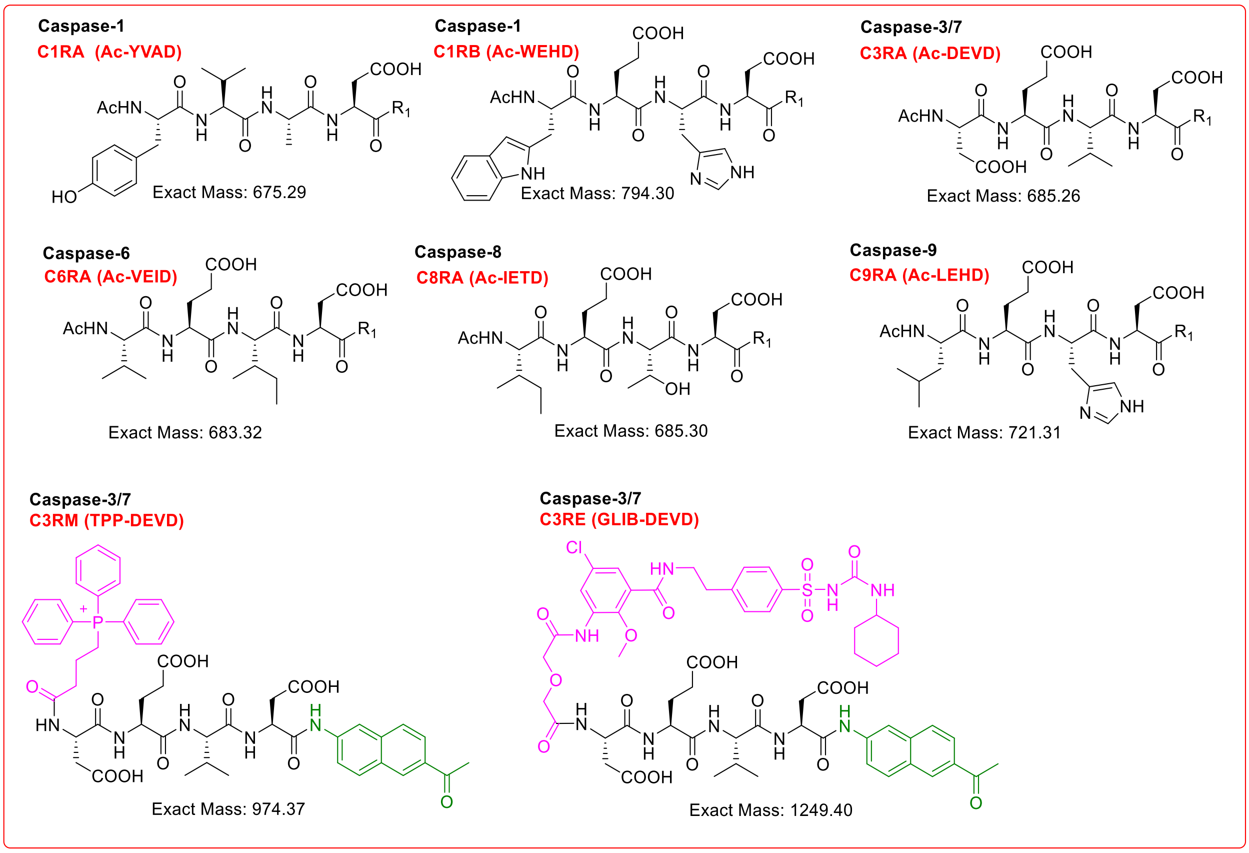

Supplementary Scheme 1. Synthesis of peptide-based caspase probes containing 6-acyl-2-naphthylamine (**AAN**). Reagents and conditions for solid-phase peptide synthesis (SPPS): (a) i: 20% piperidine in DMF, rt, 1 h; ii: P_2-amino acid, HBTU, HOBt, DIEA, DMF, rt, 4 h; (b) i: 20% piperidine in DMF, rt, 1 h; ii: P_3-amino acid, HBTU, HOBt, DIEA, DMF, rt, 4 h; (c) i: 20% piperidine in DMF, rt, 1 h; ii: P_4-amino acid, HBTU, HOBt, DIEA, DMF, rt, 4 h; iii: 20% piperidine in DMF, rt, 1 h; iv: acetic anhydride/**M1**/**E8**, DIEA, DMF (THF for **E8**), rt, 4 h; (d) 20% TFA, 2.5% TIS, 2.5% H_2O in DCM, rt, 1-2 h.

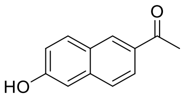

(1)

Compound **1** was synthesized using a previously published procedure.[2] To a solution containing 6-acyl-2-methoxynaphthalene (1.0 g, 5 mmol) in glacial acetic acid (9 mL), 48% HBr (4.2 g, 51.5 mmol) was added. The mixture was stirred at 100 ℃ for 12 h. Excessive acetic acid was removed *in vacuo*, and the residue was taken up in ethyl acetate (EA) and washed with saturated $NaHCO_3$ and brine. The organic layer was dried over anhydrous Na_2SO_4 and the solvent was removed *in vacuo*. The crude product was purified by flash chromatography (EA:Hexane = 1:4) to obtain **1** (0.57 g; 61% yield). **^{1}H NMR** (300 MHz, CD_3OD): δ 8.44 (d, *J* = 1.0 Hz, 1H), 7.88 (td, *J* = 8.9, 2.5 Hz, 2H), 7.66

(d, J = 8.7 Hz, 1H), 7.14 (dd, J = 6.6, 2.6 Hz, 2H), 2.65 (s, 3H). **IT-TOF-MS**: m/z $[M+H]^+$ calcd, 187.07; found, 187.06.

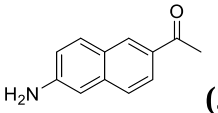

(2, AAN)

AAN was synthesized using a previously published procedure.[3] A mixture of **1** (0.260 g, 1.4 mmol), $Na_2S_2O_5$ (0.665 g, 3.5 mmol) and $NH_3 \cdot H_2O$ (7.5 mL) in a steel-bomb reactor was stirred at 140 ℃ for 120 h. The reaction mixture was extracted by EA and washed with saturated $NaHCO_3$ twice. The organic layer was dried over anhydrous Na_2SO_4 and the solvent was removed *in vacuo*. The crude product was purified by flash chromatography using dichloromethane (DCM) as the eluent to obtain **AAN** as a yellow solid (0.148 g; 57% yield). **^{1}H NMR** (300 MHz, $CDCl_3$): δ 8.34-8.28 (m, 1H), 7.93 (dd, J = 8.7, 1.8 Hz, 1H), 7.77 (d, J = 9.4 Hz, 1H), 7.60 (d, J = 8.7 Hz, 1H), 6.98 (d, J = 7.8 Hz, 2H), 4.06 (s, 2H), 2.67 (s, 3H). **^{13}C NMR** (75 MHz, $CDCl_3$): δ 197.8, 146.8, 137.6, 131.5, 131.2, 130.4, 126.5, 125.9, 124.7, 118.7, 107.9, 26.4. **ESI-HRMS**: m/z $[M+Na]^+$ calcd, 208.0733; found, 208.0728. Caution: The amount of $NH_3 \cdot H_2O$ should be limited to a small amount; otherwise, it will be easy to explode at such a high temperature.

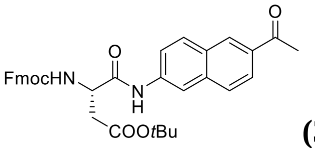

(3a)

To a solution of Fmoc-Asp(O*t*Bu)-OH (0.2468 g, 0.6 mmol) and HATU (0.2736 g, 0.72 mmol) in DMF (5mL) was added DIEA (317 μL, 1.8 mmol) and stirred for 10 min at room temperature (rt) under nitrogen atmosphere. **AAN** (0.1111 g, 0.6 mmol) was then added and the reaction was stirred overnight. The reaction mixture was diluted by EA and washed with 1M HCl, saturated $NaHCO_3$ and brine. The organic layer was dried over anhydrous Na_2SO_4 and the solvent was removed *in vacuo*. The crude product was purified by flash chromatography (EA:Hexane = 1:4) to obtain **3a** as an orange solid (0.27 g; 80% yield). **^{1}H NMR** (300 MHz, $CDCl_3$): δ 8.85 (s, 1H), 8.40 (s, 1H), 8.24 (d, J = 1.7 Hz, 1H), 8.01 (dd, J = 8.6, 1.7 Hz, 1H), 7.90 (d, J = 8.8 Hz, 1H), 7.82 (d, J = 8.7 Hz, 1H), 7.77 (d, J = 7.4 Hz, 2H), 7.60 (d, J = 7.4 Hz, 2H), 7.51 (dd, J = 8.8, 2.1 Hz, 1H), 7.40 (t, J = 7.4 Hz, 2H), 7.31 (d, J = 7.4 Hz, 2H), 6.12 (s, 1H), 4.71 (s, 1H), 4.51 (d, J = 6.7 Hz, 2H), 4.24 (t, J = 6.7 Hz, 1H), 3.00 (dd, J = 17.2, 3.9 Hz, 1H), 2.76 (d, J = 7.7 Hz, 1H), 2.71 (s, 3H), 1.48 (s, 9H). **^{13}C NMR** (75 MHz, $CDCl_3$): δ 197.9, 171.5, 168.9, 143.6, 143.5, 141.3, 137.3, 136.3, 133.8, 130.5, 129.8, 129.6, 128.1, 127.8,

127.1, 124.9, 124.7, 120.6, 120.1, 116.2, 82.4, 67.4, 51.9, 47.1, 37.2, 28.0, 26.6. **IT-TOF-MS**: *m/z* $[M+H]^+$ calcd, 579.24; found, 579.22.

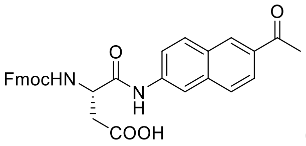

(3b)

3a (0.2360 g, 0.41 mmol) was dissolved in DCM and TFA (20% v/v) diluted in DCM was added dropwise to the solution in an ice bath. The reaction was stirred for 4 h at rt, after which, DCM and TFA were removed *in vacuo*. The crude product was purified by flash chromatography (EA:Hexane = 1:1 with 1% acetic acid) affording **3b** as a white solid (0.16 g, 74% yield). ^{1}**H NMR** (300 MHz, Acetone-d_6): δ 9.68 (s, 1H), 8.56 (s, 1H), 8.44 (s, 1H), 8.09-7.95 (m, 2H), 7.88 (dd, *J* = 24.1, 7.9 Hz, 3H), 7.74 (dd, *J* = 21.1, 7.8 Hz, 3H), 7.39 (d, *J* = 4.8 Hz, 2H), 7.35-7.25 (m, 2H), 7.11 (d, *J* = 7.9 Hz, 1H), 4.78 (d, *J* = 7.7 Hz, 1H), 4.40 (dt, *J* = 17.5, 10.5 Hz, 2H), 4.26 (t, *J* = 6.8 Hz, 1H), 3.04 (dd, *J* = 16.7, 6.3 Hz, 1H), 2.90 (dd, *J* = 6.9 Hz, 1H), 2.68 (s, 3H). ^{13}**C NMR** (75 MHz, Acetone-d_6): δ 197.7, 172.3, 170.6, 157.3, 145.0, 142.1, 139.8, 137.2, 134.6, 131.2, 130.9, 130.4, 128.6, 128.0, 126.2, 125.2, 121.7, 120.9, 116.4, 67.5, 55.0, 53.5, 48.0, 36.6, 26.7. **ESI-HRMS**: *m/z* $[M+Na]^+$ calcd, 545.1683; found, 545.1694.

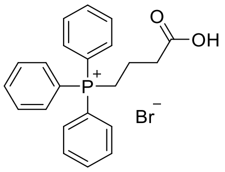

(M1)

A solution of triphenylphosphine (1.67 g, 10 mmol) and 4-bromobutanoic acid (2.62 g, 10 mmol) in toluene was heated and subjected to reflux for 48 h to produce **M1** as white solid (1.48 g, 34% yield) which was filtrated and washed with toluene.[4] ^{1}**H NMR** (300 MHz, CD_3OD): δ 6.53-6.13 (m, 15H), 1.97 (ddd, *J* = 16.6, 8.4, 4.8 Hz, 2H), 1.09 (t, *J* = 6.6 Hz, 2H), 0.42 (dd, *J* = 16.2, 7.2 Hz, 2H).

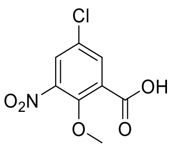

(E3)

To a stirred solution of methyl 5-chlorosalicylate (5 g, 26.8 mmol) in DMF (40 mL) was added K_2CO_3 (11.1 g, 80.0 mmol), followed by iodomethane (4.2 g, 29.5 mmol). The mixture was stirred for 4 h at rt, TLC showed (EA:Hexane = 1:4) that reaction was complete. 200 mL of water was added to the mixture and extracted with EA (2 ×100 mL). The combined organic layers were washed with brine,

dried over Na_2SO_4, and evaporated to afford the desired product **E1** as yellow oil, which was contaminated with DMF and used for next step without further purification.

The above residue was dissolved in ethanol (30 mL) and to which an aqueous solution of NaOH (3.2 g, 80.4 mmol) in water (30 mL) was added. After being stirred for 1-3 min, white solid precipitates, and the mixture was continued to stir for 1 h. TLC showed (EA:Hexane = 1:1) starting material had disappeared. The mixture was concentrated to remove most part of ethanol and acidified with 1 M HCl to *p*H = 2-3 and extracted with EA (2 ×100 mL). The combined organic layers were washed with brine, dried over Na_2SO_4, and evaporated to afford the desired product **E2** as yellowish solid, which was used for next step without further purification.

To the crude **E2** obtained above was added concentrated sulfuric acid 50 mL to dissolve the solid and the solution was cooled to 0 ℃. 1.9 mL of concentrated nitric acid was added dropwise in 2 min. After the addition, the solution was stirred for 15 min, and TLC showed (EA:Hexane = 1:1) the reaction finished. The solution was poured into ice in a 250 mL of conical flask, and the product was collected by filtration, and the filter cake was washed with a large amount of water to completely remove the acid. The solid was dried to afford the desired product **E3** (5.4 g, 87% yield for three steps) as off-white solid.

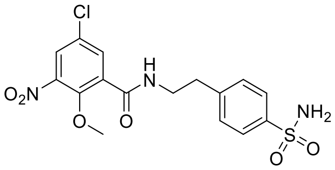

(E4)

To a stirred solution of **E3** (5 g, 21.6 mmol) in THF cooled to 0 ℃ was added Et_3N (4.4 g, 43.2 mmol), followed by tert-butyl chloroformate (4.3 g, 32.4 mmol) at 0 ℃. The solution was stirred for 1 h, and TLC showed (EA:Hexane = 1:1) the reaction finished. Volatiles were removed under vacuum and DMF (50 mL) was added, followed by a solution of 4-(2-aminoethyl)benzenesulfonamide (4.7 g, 23.7 mmol) in DMF (20 mL) at rt. The mixture was stirred for 2 h, TLC showed (EA:Hexane = 1:1) the reaction completely finished. 200 mL of water was poured into the reaction mixture and the product was collected by filtration, and the filter cake was washed with water to completely remove DMF. The solid was dried to afford the desired product **E4** (7.6 g, 85% yield) as off-white solid. **^{1}H NMR** (500 MHz, $CDCl_3$) δ: 8.69 (t, *J* = 5.6, 1H), 8.14 (d, 1H), 7.75 (d, 2H), 7.71 (d, *J* = 2.6, 1H), 7.46 (d, *J* = 3.3, 2H), 7.30 (s, 2H), 3.68 (s, 3H), 3.55 (pseudo-t, 2H), 2.92 (t, *J* = 6.6, 2H). **^{13}C NMR** (125 MHz, $CDCl_3$)

δ: 163.98, 148.85, 144.95, 143.88, 142.65, 134.45, 133.37, 129.68, 127.95, 126.15, 125.88, 63.66, 40.74, 34.82.

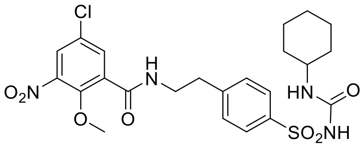
(E5)

To a stirred solution of triphosgene (2.2 g, 7.4 mmol) in toluene at rt was added cyclohexylamine (1.4 g, 14.5 mmol) dropwisely. The mixture was then refluxed for 4 h (oil bath 125 ℃) and later cooled to rt to remove small amount of precipitate. The filtrate was concentrated and used for next step without further purification.

To the above residue was added a solution of **E4** (2 g, 4.83 mmol) in DMF (20 mL), followed by CuI (0.46 g, 2.4 mmol). However, after overnight stirring, no reaction occurs. Maybe some HCl from isocyanate quenched the reaction, so 3 equivalent of K_2CO_3 was added to the reaction and stirred overnight again. TLC showed (EA:Hexane = 1:1) around 20% starting material remained, but some side products were produced, so the reaction was quenched with 200 mL of water and filtered. The filtrate was acidified with 1 M HCl to *p*H = 2-3 and extracted with EA (2 × 50 mL). The combined organic layers were washed with brine, dried over Na_2SO_4, and evaporated. The resulting residue was washed with a mixture of EA and Hexane (1:1) and then crystallized in acetone to afford the desired product **E5** (0.6 g, 23% yield) as yellowish solid. **^{1}H NMR** (500 MHz, DMSO-d_6) δ: 10.29 (s, 1H), 8.68 (t, *J* = 5.5, 1H), 8.14 (d, 1H), 7.82 (d, 2H), 7.71 (d, *J* = 2.6, 1H), 7.50 (d, *J* = 8.2, 2H), 6.32 (d, *J* = 6.0, 1H), 3.61 (s, 3H), 3.58 (pseudo-q, 2H), 3.28-3.24 (m, 1H), 2.95 (t, *J* = 7.1, 2H), 1.65-1.63 (m, 2H), 1.59-1.56 (m, 2H), 1.49-1.47 (m, 1H), 1.24-1.17 (m, 2H), 1.13-1.06 (m, 3H). **^{13}C NMR** (125 MHz, DMSO-d_6) δ: 163.42, 150.40, 148.31, 144.99, 144.49, 138.24, 133.91, 132.89, 129.28, 127.45, 127.28, 125.38, 60.02, 48.08, 39.99, 34.33, 32.26, 24.96, 24.16.

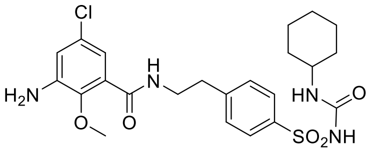
(E6)

To a stirred solution of **E5** (2 g, 3.72 mmol) in acetic acid/ethanol (70 mL/70 mL) at rt was added Fe powder (1.4 g, 14.5 mmol). The mixture was then heated at 60 ℃ for 2.5 h and cooled to rt afterwards (Note: about 5% of starting material still remained, this reaction should be monitored by TLC and LCMS to avoid over-reduction, over reduced product is completely overlapped with the

product). 1000 mL of water was added to collect precipitate by filtration. The crude product was dissolved in DCM (50 mL) and 1 M HCl (50 mL) was added. The mixture was stirred for 1 h, during which, white solid precipitates were collected by filtration to afford the pure product **E6** (1.4 g, 74% yield) as off-white solid. **^{1}H NMR** (500 MHz, DMSO-d_6) δ: 10.29 (s, 1H), 8.28 (t, *J* = 5.5, 1H), 7.82 (d, 2H), 7.49 (d, *J* = 8.2, 2H), 6.76 (d, 1H), 6.58 (d, *J* = 2.5, 1H), 6.32 (d, *J* = 7.4, 1H), 5.37 (b s, 2H), 3.55 (pseudo-q, 2H), 3.46 (s, 3H), 3.31-3.24 (m, 1H), 2.94 (t, *J* = 7.0, 2H), 1.65-1.62 (m, 2H), 1.58-1.56 (m, 2H), 1.49-1.46 (m, 1H), 1.28-1.17 (m, 2H), 1.13-1.06 (m, 3H). **^{13}C NMR** (125 MHz, DMSO-d_6) δ: 165.01, 150.41, 145.24, 143.50, 142.32, 138.14, 130.46, 129.24, 127.94, 127.29, 115.30, 114.72, 60.00, 48.01, 39.85, 34.54, 32.28, 24.98, 24.17.

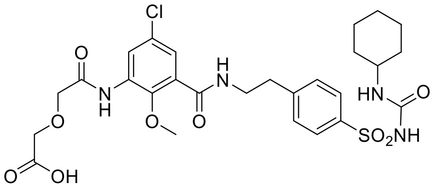

(E7)

To the stirred solution of **E6** (0.9 g, 1.77 mmol) in DMF (5 mL), Et_3N (1.0 mL, 7.3 mmol) was diglycolic anhydride (620 mg, 5.31 mmol) at rt. The mixture was stirred for 8 h, TLC showed (DCM/methanol =10:1) that about 20% starting material still remained. But prolonged time did not make the reaction finished. The reaction was washed with saturated $NaHCO_3$ solution and extracted with EA (2 × 30 mL). The combined organic layers were evaporated to recycle starting material **E6** (200 mg). The aqueous phase was acidified to *p*H = 2-3 with 2 M HCl and extracted with EA (2 × 50 mL). The combined organic layers were washed with brine and dried over Na_2SO_4 and evaporated to afford the crude **E7** (0.75 g), which was treated with DCM (25 mL) and Hexane (25 mL) and collected by filtration to give the desired product **E7** (450 mg, 56%) as off-white solid. **^{1}H NMR** (500 MHz,CD_3OD) δ: 8.36 (d, 1H), 7.91 (d, 2H), 7.52 (d, *J* = 7.8, 2H), 7.30 (d, *J* = 2.5, 1H), 4.26 (s, 2H, CH_2), 4.24 (s, 2H), 3.70 (t, 2H), 3.63 (s, 3H), 3.43-3.38 (m, 1H), 3.05 (t, *J* = 7.0, 2H), 1.75-1.73 (m, 2H), 1.66-1.64 (m, 2H), 1.57-1.55 (m, 1H), 1.35-1.28 (m, 2H), 1.22-1.12 (m, 3H). **^{13}C NMR** (125 MHz, CD_3OD) δ: 170.46, 167.31, 147.83, 146.78, 133.64, 130.83, 130.63, 130.39, 128.80, 125.43, 124.10, 71.87, 62.88, 50.15, 41.68, 35.96, 33.79, 26.47, 25.72.

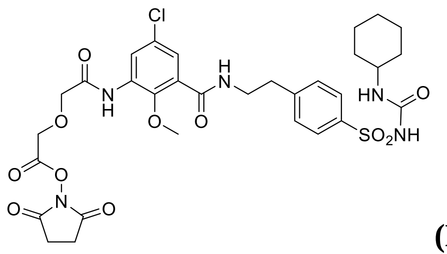

(E8)

In an ice bath, *N*-hydroxysuccinimide and *N,N'*-dicyclohexylcarbodiimide (DCC) were added to a solution of **E7** in anhydrous THF. The suspension was filtered off and the filtrate was used for the subsequent coupling reaction directly.

General Procedures for Solid-Phase Peptide Synthesis (SPPS). The peptide was synthesized on 2-chlorotrityl chloride resin (loading ~0.5 mmol/g) using Irori microkan reactors.[5] Briefly, 2-chlorotrityl chloride resins were first activated by refluxing with $SOCl_2$ in DCM for 4 h before placing them into reactors. In the loading step, to a solution of compound **3b** (0.30 mmol, 4 eq) dissolved in dry DCM (5 ml) and DIEA (0.40 mmol, 8 eq) was added the resins (150 mg, 0.075 mmol, 1eq) and shaken overnight at rt. The resulting resins were filtered and washed thoroughly with DMF (3×), DCM (3×) and DMF (3×) until the filtrate became colorless. Upon washing with MeOH for 1 h at rt and filtration, the resins were washed with DMF (3×), DCM (3×) and DMF (3×). The Fmoc-protected amino-functionalized resin was treated with 20% v/v piperidine in DMF for 1 h at rt. Successful loading of compound **3b** was confirmed by the Ninhydrin test where the presence of a primary amine was indicated by the blue color of the resin. The amino-functionalized resins were then added to pre-activated solution of Fmoc-protected amino acid (0.30 mmol, 4 eq), HOBt (0.30 mmol, 4 eq), HBTU (0.30 mmol, 4 eq) and DIEA (0.60 mmol, 8 eq) in DMF (5 mL). The coupling reaction was carried out for 4 h at rt with constant shaking. Complete coupling of the P_2 amino acid was confirmed by the Ninhydrin test where there was no change in color. Repeated cycles of Fmoc-deprotection and coupling of Fmoc-protected amino acids resulted in peptide elongation. Washing cycles were carried out after every reaction. Finally, the *N*-terminus of the P_4 amino acid was capped with Ac_2O (0.20 mmol, 4 eq)/**M1** (0.20 mmol, 4 eq)/**E8** (0.20 mmol, 4 eq) and DIEA (0.40 mmol, 8 eq) in DMF (5 mL)/THF (5 mL for **E8**). The peptide was cleaved from the solid support by using a cleavage cocktail of TFA:TIS:H_2O:DCM (20:2.5:2.5:75) to the resins and shaken for 1-2 h at rt. The filtrate was concentrated to ~0.3 mL *in vacuo* and then precipitated by addition of cold diethyl ether (5 mL). The precipitated peptide was collected by centrifugation and further purified by preparative HPLC to afford

the pure product as white solid. All peptides were characterized by LC-MS (IT-TOF) and ESI-HRMS.

C1RA (Ac-YVAD-AAN, $C_{35}H_{41}N_5O_9$): *m/z*[M+H]$^+$calcd, 676.2977; ESI-HRMS found, 676.3005.

C1RB (Ac-WEHD-AAN, $C_{40}H_{42}N_8O_{10}$): *m/z*[M+H]$^+$calcd, 795.3097; ESI-HRMS found, 795.3114.

C3RA (Ac-DEVD-AAN, $C_{32}H_{39}N_5O_{12}$): m/z[M+Na]$^+$calcd, 708.2487; ESI-HRMS found, 708.2503.

C6RA (Ac-VEID-AAN, $C_{34}H_{45}N_5O_{10}$): m/z[M+Na]$^+$calcd, 706.3059; ESI-HRMS found, 706.3058.

C8RA (Ac-IETD-AAN, $C_{33}H_{43}N_5O_{11}$): m/z[M+Na]$^+$calcd, 708.28519; ESI-HRMS found, 708.2863.

C9RA (Ac-LEHD-AAN, $C_{35}H_{43}N_7O_{10}$): m/z[M+Na]$^+$calcd, 744.2964; ESI-HRMS found, 744.2961.

C3RM (TPP-DEVD-AAN, $C_{52}H_{57}N_5O_{12}P^+$): m/z[M]$^+$ calcd, 974.3736; ESI-HRMS found, 974.3733.

C3RE (GLIB-DEVD-AAN, Chemical Formula: $C_{57}H_{68}ClN_9O_{19}S$): m/z[M-3H]$^{3-}$ calcd, 415.4607; found, 415.4610.

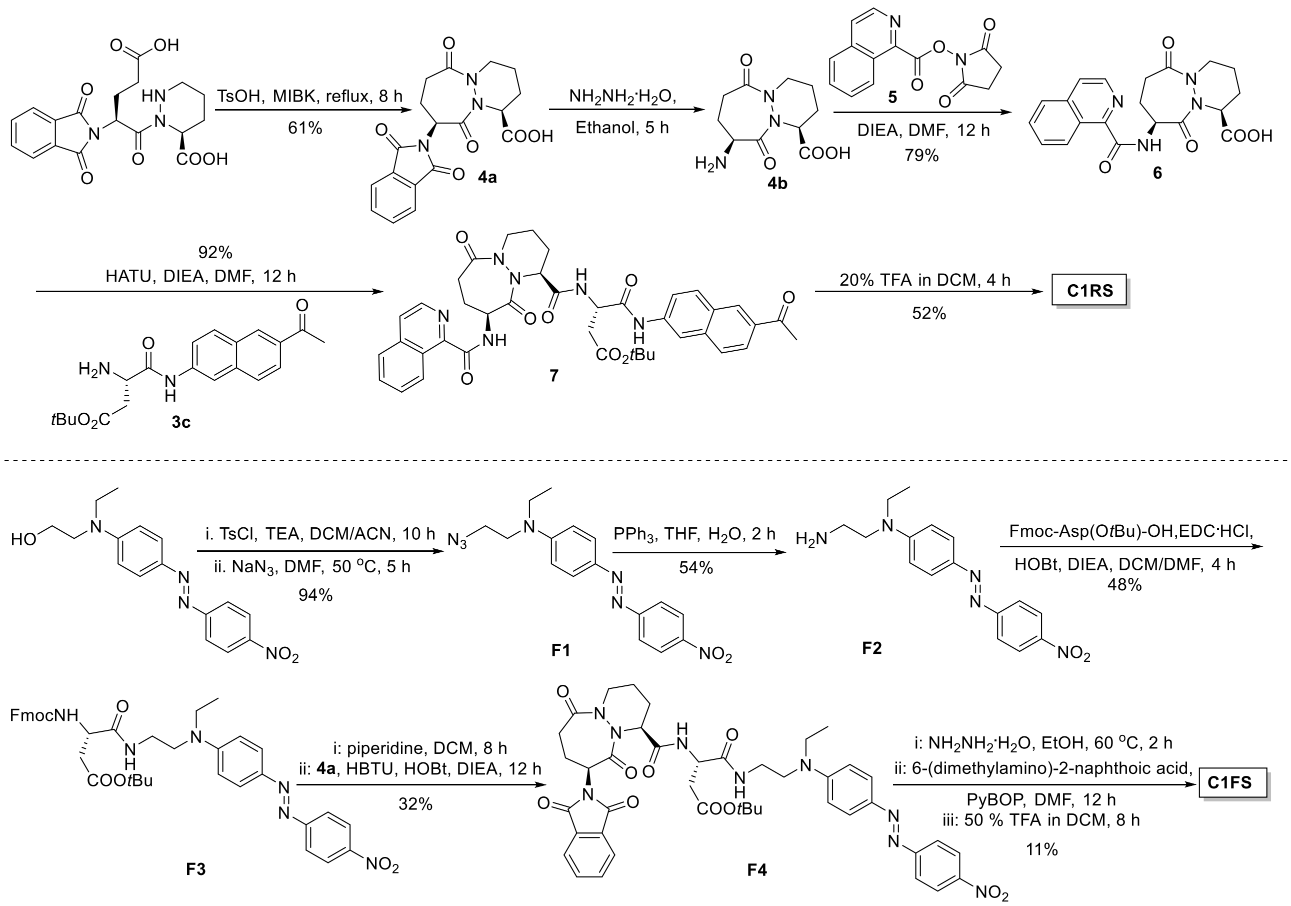

Supplementary Scheme 2. Synthesis of **C1RS** and **C1FS** derived from Pralnacasan.

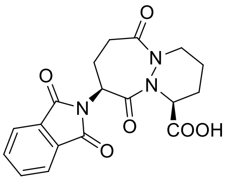

(4a)

To the suspension of (*S*)-2-((*S*)-4-carboxy-2-(1,3-dioxoisoindolin-2-yl)butanoyl)hexahydro-pyridazine-3-carboxylic acid[1] (6.0 g, 15.4 mmol) in methyl isobutyl ketone (65 mL) was added *p*-toluenesulfonic acid (100 mg, 0.58 mmol). The resulting mixture was refluxed for 8 h. Then the mixture was cooled down to rt. The solid was collected by filtration and then this solid was suspended in ethanol (50 mL) and filtered again to give the desired product (3.16 g, 61.4%). **^{1}H NMR** (500 MHz, DMSO-d_6): δ 13.3 (brs, 1H), 7.92-7.87 (m, 4H), 5.21 (dd, *J* = 3.0 Hz, *J* = 6.0 Hz, 1H), 5.16 (dd, *J* = 9.0 Hz, *J* = 11.5 Hz, 1H), 4.47 (m, 1H), 3.50-3.43 (m, 1H), 3.24-3.17 (m, 1H), 2.91 (dt, *J* = 3.5 Hz, *J* = 12.5 Hz, 1H), 2.39-2.29 (m, 2H), 2.12-2.08 (m, 1H), 1.84-1.78 (m, 1H), 1.71-1.67 (m, 1H), 1.54-1.49 (m, 1H). **^{13}C NMR** (125 MHz, DMSO-d_6): δ 171.8, 171.4, 167.9, 167.6, 135.3, 123.8, 52.8, 50.1, 41.3, 30.1, 25.8, 25.1, 20.4.

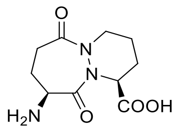

(4b)

To a suspension of **4a** (75 mg, 0.20 mmol) in ethanol was added hydrazine monohydrate (22 μL, 0.44 mmol) and the reaction was allowed to stir at rt for 5 h. The reaction mixture was concentrated *in vacuo* to afford white solid. The crude product was directly used without further purification. **ESI-MS**: *m/z* $[M+H]^+$ calcd, 242.11; found, 242.12.

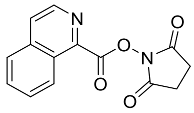

(5)

N-hydroxysuccinimide (NHS, 0.43 g, 3.80 mmol) and EDC (0.90 g, 5.80 mmol) were added to a solution of isoquinolin-1-carboxylic acid (0.60 g, 3.45 mmol) in DMF. The reaction was stirred at rt overnight. The reaction mixture was taken up by DCM and washed with saturated $NaHCO_3$ and brine. The crude product was purified by flash chromatography by using EA:Hexane = 1:1 to obtain **5** as white powder (0.75 g; 81% yield). **^{1}H NMR** (300 MHz, $CDCl_3$): δ 8.83 (m, 1H), 8.74 (d, *J* = 5.5 Hz, 1H), 7.94 (m, 2H), 7.76 (m, 2H), 2.97 (s, 4H). **^{13}CNMR** (75 MHz, $CDCl_3$): δ 169.6, 161.3, 144.3, 142.7, 137.5, 131.7, 130.4, 128.4, 128.1, 126.6, 126.3, 26.5.

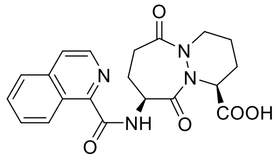

(6)

Compound **5** (66 mg, 0.24 mmol) and DIEA (176 μL, 1.01 mmol) were added to a solution of **4b** (48 mg, 0.20 mmol) in DMF (1 mL). The reaction was stirred at rt overnight. The reaction mixture was acidified to *p*H 3 with 2M HCl to convert the carboxylate to the acid and extracted with EA. The organic layer was dried over anhydrous Na_2SO_4 and solvent was removed *in vacuo*. The crude product was purified by flash chromatography using 1% acetic acid in EA to afford **6** as white solid (0.063 g; 79% yield). **^{1}H NMR** (300 MHz, $CDCl_3$, CD_3OD): δ 9.34 (d, *J* = 6.1 Hz, 1H), 8.46 (dd, *J* = 5.5, 1.2 Hz, 1H), 7.81 (d, *J* = 8.0 Hz, 1H), 7.78 (d, *J* = 5.5 Hz, 1H), 7.71-7.58 (m, 2H), 5.37 (d, *J* = 3.2 Hz, 1H), 5.17-5.00 (m, 1H), 4.59 (d, *J* = 12.8 Hz, 1H), 3.64-3.44 (m, 1H), 3.01-2.87 (m, 1H), 2.86-2.70 (m, 1H), 2.40-2.21 (m, 2H), 2.12-1.97 (m, 1H), 1.95-1.80 (m, 1H), 1.78-1.60 (m, 2H). **^{13}C NMR** (75 MHz, $CDCl_3$, CD_3OD): δ 173.2, 172.0, 170.7, 166.5, 146.8, 141.1, 138.0, 135.3, 131.2, 129.4, 127.9, 127.5, 125.2, 49.1, 42.1, 31.7, 30.5, 25.8, 24.0, 20.9.

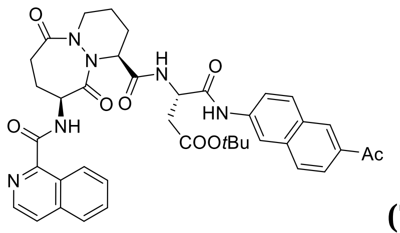

(7)

To a solution of **6** (6 mg, 0.015 mmol) and HATU (8.6 mg, 0.023 mmol) in DMF was added DIEA (8 μL, 0.046 mmol) and stirred for 10 min at rt under nitrogen atmosphere. **3c** (7.4 mg, 0.021 mmol; obtained from reaction of **3a** with 20% piperidine in $CHCl_3$ for 2 h and purified by flash chromatography) was then added slowly and the resulting mixture was allowed to stir overnight. The reaction mixture was diluted by EA and washed with saturated NH_4Cl, $NaHCO_3$ and brine. The organic layer was dried over anhydrous Na_2SO_4 and solvent was removed *in vacuo*. The crude product was purified by flash chromatography (EA:Hexane = 1:1) to obtain **7** as white solid (10.2 mg; 92% yield). **^{1}H NMR** (300 MHz, $CDCl_3$): δ 9.51 (d, *J* = 7.9 Hz, 1H), 9.12 (s, 1H), 8.92 (d, *J* = 7.7 Hz, 1H), 8.52 (d, *J* = 5.5 Hz, 1H), 8.40 (s, 1H), 8.25 (s, 1H), 8.01 (d, *J* = 6.9 Hz, 1H), 7.94-7.78 (m, 4H), 7.72 (dd, *J* = 13.7, 6.5 Hz, 2H), 7.57 (d, *J* = 7.3 Hz, 1H), 7.53-7.46 (m, 1H), 5.27 (d, *J* = 4.7 Hz, 1H), 4.91 (s, 1H), 4.63 (s, 1H), 3.40 (d, *J* = 8.9 Hz, 1H), 3.10 (s, 1H), 2.96 (dd, *J* = 18.9, 15.5 Hz, 2H), 2.70 (d, *J* = 3.3 Hz, 3H), 2.51 (d, *J* = 12.9 Hz, 1H), 2.09 (d, *J* = 12.0 Hz, 7H), 1.49 (d, *J* = 3.2 Hz, 9H). **^{13}C NMR** (75 MHz, $CDCl_3$): δ 197.8, 171.9, 171.8, 171.0, 169.4, 168.3, 165.6, 147.2, 140.4, 137.3, 137.2, 136.2, 133.7, 130.6, 130.5, 129.8, 129.6, 128.7, 128.1, 127.4, 127.0, 126.8, 124.7, 124.6, 120.4, 116.1, 82.8, 53.9, 50.4, 48.6, 41.6, 36.4, 31.2, 30.2, 29.6, 28.0, 26.6, 24.7, 19.3. **IT-TOF-MS**: *m/z* $[M+H]^+$ calcd, 735.31; found, 735.29.

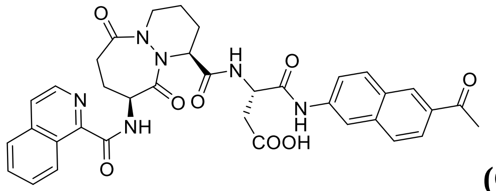

(C1RS)

Compound **7** (10.2 mg, 0.014 mmol) dissolved in CH_2Cl_2 was added TFA (20% v/v, diluted in CH_2Cl_2) at 0 ^{o}C. The reaction was warmed up and further stirred at room temperature for 4 h. The reaction mixture was concentrated, and the resulting residue was purified by flash chromatography (EtOAc:Hexane = 50:1 with 1% acetic acid) to afford **C1RS** as a pale brown solid (3.9 mg; 52% yield). ^{1}H NMR (300 MHz, DMSO-d_6): δ 9.05 (d, *J* = 9.0 Hz, 2H), 8.61-8.52 (m, 3H), 8.32 (s, 1H), 8.12-8.01 (m, 3H), 7.92-7.62 (m, 5H), 5.23 (d, *J* = 11.5 Hz, 1H), 4.92 (d, *J* = 23.1 Hz, 1H), 4.66 (d, *J* = 19.3 Hz,

1H), 4.37 (d, *J* = 9.5 Hz, 1H), 4.12 (t, *J* = 7.2 Hz, 1H), 3.92 (d, *J* = 5.6 Hz, 2H), 2.67 (s, 3H), 2.27 (t, *J* = 7.2 Hz, 2H), 2.19-2.05 (m, 4H), 1.98 (s, 2H).^{13}C NMR (75 MHz, DMSO-d_6): δ 198.0, 175.2, 172.0, 171.6, 170.1, 170.0, 165.9, 150.1, 141.2, 139.3, 137.0, 136.1, 133.3, 131.1, 130.8, 130.4, 129.1, 128.9, 128.0, 127.5, 127.0, 126.0, 124.4, 124.2, 121.1, 115.2, 52.6, 51.1, 48.8, 44.1, 36.2, 30.2, 29.7, 27.0, 26.1, 19.4. ESI-HRMS: *m/z*$[M+Na]^+$calcd., 701.2330; found, 701.2358.

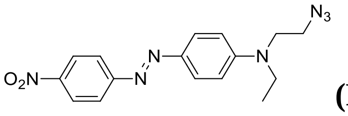

(F1)

To the solution of (*E*)-3-(4-((4-nitrophenyl)diazenyl)phenyl)pentyl 4-methylbenzenesulfonate[6] (2.0 g, 4.26 mmol) in DMF (30 mL) was added sodium azide (415 mg, 6.39 mmol). The reaction mixture was stirred at 50 °C for 5 h. Then reaction mixture was cooled down to rt. EA (100 mL) and water (50 mL) were added. The organic layer was taken. The aqueous layer was further extracted with EA (50 mL ×2). The combined organic layer was washed with water (50 mL ×2), brine (50 mL), dried over Na_2SO_4 and purified with flash chromatography to give the product (1.35 g, 93.8% yield). **^{1}H NMR** (300 MHz, $CDCl_3$): δ 8.31 (dd, *J* = 9.0Hz, *J* = 2.4 Hz, 2H), 7.91-7.87 (m, 4H), 6.78 (d, *J* = 8.1 Hz, 2H), 3.61-3.51 (m, 6H), 1.25 (t, *J* = 6.9 Hz, 3H). **^{13}C NMR** (75 MHz, $CDCl_3$): δ 156.6, 150.7, 147.3, 143.8, 126.1, 124.5, 122.6, 111.4, 49.4, 48.8, 45.8, 12.2. **IT-TOF-MS**: *m/z* $[M+H]^+$ calcd, 340.15; found, 340.13.

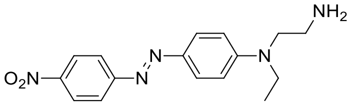

(F2)

To the solution of **F1** (900 mg, 2.65 mmol) in THF (30 mL) and water (58 mg) was added triphenylphosphine (1.04 g, 3.97 mmol). The reaction mixture was stirred at rt for 2 h. Then more water (58 mg) was added. The resulting mixture was stirred at rt for 24 h. Then all the solvent was removed *in vacuo*. The desired residue was purified with flash chromatography to give **F2** (463 mg, 53.8% yield). **^{1}H NMR** (500 MHz, DMSO-d_6): δ 8.36 (d, *J* = 8.5 Hz, 2H), 7.93 (d, *J* = 9.0 Hz, 2H), 7.83 (d, *J* = 9.0 Hz, 2H), 6.89 (d, *J* = 9.0 Hz, 2H), 3.51 (m, 2H), 3.43 (t, *J* = 7.5 Hz, 2H), 2.76 (t, *J* = 7.5 Hz, 2H), 1.15 (t, *J* = 7.5 Hz, 3H). **^{13}C NMR** (125 MHz, DMSO-d_6): δ 156.2, 151.7, 146.6, 142.4, 126.1, 124.9, 122.3, 111.4, 52.8, 45.0, 12.1. **IT-TOF-MS**: *m/z* $[M+H]^+$ calcd, 314.16; found, 314.15.

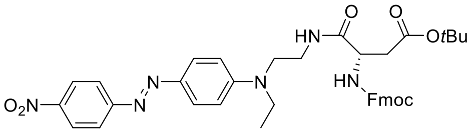

(F3)

To the solution of Fmoc-Asp(O*t*Bu)-OH (1.6 g, 3.9 mmol) in DCM (20 mL) and DMF (10 mL) was added EDC·HCl (1.01 g, 5.26 mmol), HOBt (240 mg, 1.78 mmol) and DIEA (512 mg, 3.96 mmol). Then **F2** (1.1 g, 3.5 mmol) was added to the above solution. The mixture was stirred at rt for 4 h. To the mixture was added DCM (80 mL) and water (30 mL). The organic layer was taken, washed with water (30 mL ×3), brine (30 mL), dried over Na_2SO_4 and purified with flash chromatography to give the product (1.2 g, 48.4% yield). **^{1}H NMR** (500 MHz, $CDCl_3$): δ 8.29 (d, *J* = 9.0Hz, 2H), 7.89 (d, *J* = 9.0Hz, 2H), 7.87 (d, *J* = 9.0Hz, 2H), 7.74 (m, 2H), 7.54 (d, *J* = 7.5Hz, 2H), 7.40 (m, 2H), 7.26 (m, 2H), 6.82 (d, *J* = 9.0Hz, 2H), 6.71 (brs, 1H), 5.91 (d, *J* = 6.0Hz, 1H), 4.48 (brs, 1H), 4.42 (d, *J* = 6.0Hz, 2H), 4.18 (t, *J* = 6.0Hz, 1H), 3.53-3.49 (m, 6H), 2.95 (m, 1H), 2.61 (m, 1H), 1.45 (s, 9H), 1.22 (t, *J* = 7.0 Hz, 3H). **^{13}C NMR** (125 MHz, $CDCl_3$): δ 171.0, 156.7, 151.2, 147.3, 143.8, 143.6, 143.5, 141.4, 141.3, 127.8, 127.7, 127.0, 126.3, 124.6, 122.5, 120.0, 111.4, 82.0, 67.1, 51.2, 49.1, 47.1, 45.5, 37.5, 37.0, 28.0, 12.2. **ESI-MS**: *m/z* $[M+H]^+$ calcd, 707.32; found, 707.35.

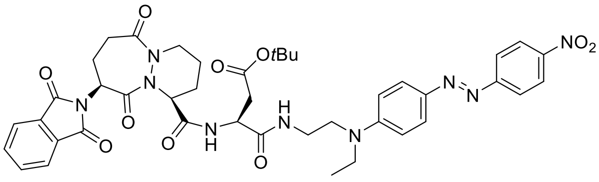

(F4)

To the solution of **F3** (1.0 g, 1.41 mmol) in DCM (15 mL) was added piperidine (3 mL). The resulting mixture was stirred at rt for 8 h. Then all the solvent was evaporated *in vacuo*. The residue containing free amine product was re-dissolved in DMF (10 mL). To a separated RBF was added DMF (20 mL), **4a**, (548 mg, 1.47 mmol), HBTU (834 mg, 2.2 mmol), HOBt (108 mg, 0.8 mmol) and DIEA (170 mg, 1.31 mmol). The resulting mixture was stirred at rt for 10 min. Then the above amine solution was added and the resulting mixture was stirred at rt overnight. Then most of the solvent was removed *in vacuo*. To the residue was added water (100 mL) and EA (150 mL). The organic layer was taken, washed with water (50 mL ×3), brine (50 mL), dried over Na_2SO_4, filtered and concentrated, the residue was purified with flash chromatography (EA: Hexane =10: 1 to 1:1) to give the product (400 mg, 32.3% yield). **^{1}H NMR** (500 MHz, $CDCl_3$): δ 8.24 (d, *J* = 9.0 Hz, 2H), 7.83-7.77 (m, 6H), 7.69 (m, 2H), 7.40 (d, *J* = 7.5 Hz, 1H), 6.97 (m, 1H), 6.79 (m, 2H), 5.29 (dd, *J* = 9.0 Hz, *J* = 11.5 Hz, 1H), 5.14 (m, 1H), 4.69-4.65 (m, 1H), 4.60-4.57 (m, 1H), 3.41-3.33 (m, 7H), 3.01-2.97 (m, 1H), 2.84 (dd, *J* = 4.5 Hz, *J* = 17.0 Hz, 1H), 2.64 (dd, *J* = 7.0 Hz, *J* = 17.0 Hz, 1H), 2.46-2.33 (m, 3H), 2.01-1.97 (m, 1H), 1.85-1.78 (m, 2H), 1.62-1.58 (m, 1H), 1.42 (s, 9H), 1.19 (t, *J* = 7.0 Hz, 3H). **^{13}C NMR** (125 MHz, $CDCl_3$): δ

172.3, 170.6, 169.0, 168.2, 167.7, 156.6, 151.2, 147.1, 143.6, 134.2, 126.2, 124.5, 123.4, 122.4, 111.3, 82.0, 53.3, 49.8, 49.5, 48.9, 45.3, 41.5, 37.4, 36.4, 30.3, 27.9, 26.0, 24.8, 19.2, 12.1. **ESI-MS**: *m/z* $[M+H]^+$ calcd, 838.35; found, 838.35.

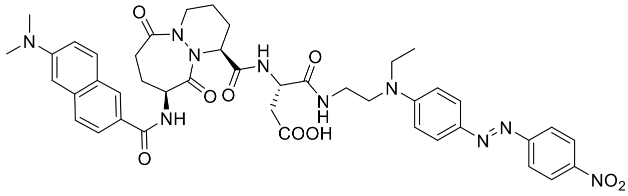

(C1FS)

To the solution of **F4** (97 mg, 0.12 mmol) in ethanol (10 mL) was added hydrazine hydrate (60 mg, 1.2 mmol). The resulting mixture was stirred at 60 °C for 2 h. Upon solvent evaporation, the residue which contained the desired free amine was re-dissolved in DMF (2 mL). To a separate reaction vessel was added DMF (3 mL), 6-(dimethylamino)-2-naphthoic acid (40 mg, 0.18 mmol) and PyBOP (125 mg, 0.24 mmol). The resulting mixture was stirred at room temperature for 10 min. Then the above amine solution was added. The resulting mixture was stirred at rt overnight. Upon solvent evaporation, the resulting residue was added water (20 mL) and EtOAc (50 mL). The organic layer was washed with water (3 × 20 mL), brine (20 mL), dried over Na_2SO_4, filtered and concentrated, before being purified by flash chromatography (EtOAc:Hexane = 10:1 to 1:1) to give the coupling product which was further dissolved in a mixture of CH_2Cl_2 (5 mL) and TFA (5 mL). The mixture was stirred at room temperature for 8 h. Upon solvent evaporation, the resulting residue was purified by preparative TLC (CH_2Cl_2:MeOH = 20:1) to give the final product **C1FS** (11.4 mg, 11.2% yield). ^{1}H NMR (300 MHz, DMSO-d_6): δ 8.50 (d, *J* = 7.5 Hz, 1H), 8.44 (d, *J* = 7.5 Hz, 1H), 8.36 (m, 2H), 8.31 (brs, 1H), 8.15 (m, 1H), 7.93 (m, 2H), 7.79-7.85 (m, 4H), 7.69 (d, *J* = 8.5 Hz, 1H), 7.28 (dd, *J* = 2.5 Hz, *J* = 9.0 Hz, 1H), 6.95 (d, *J* = 2.0 Hz, 1H), 6.93 (d, *J* = 9.5 Hz, 2H), 5.13 (t, *J* = 5.0 Hz, 1H), 4.87-4.92 (m, 1H), 4.53 (q, *J* = 7.0 Hz, 1H), 4.41 (m, 1H), 3.12-3.37 (m, 3H) 3.40-3.50 (m, 4H), 3.04 (m, 6H), 2.94 (m, 1H), 2.62 (dd, *J* = 6.0 Hz, *J* = 16.0 Hz, 1H), 2.48 (m, 1H), 2.34 (m, 1H), 2.17-2.24 (m, 2H), 2.04 (m, 1H), 1.90 (m, 1H), 1.68 (m, 1H), 1.60 (m, 1H), 1.14 (t, *J* = 7.0 Hz, 3H).^{13}C NMR (125 MHz, DMSO-d_6):δ 172.3, 169.8, 166.9, 156.7, 152.0, 150.0, 147.2, 143.2, 136.7, 130.2, 128.1, 126.8, 126.6, 126.0, 125.4, 125.1, 124.9, 122.9, 117.1, 111.9, 105.4, 52.8, 50.3, 49.2, 49.1, 45.4, 41.4, 37.1, 30.4, 29.2, 25.9, 19.5, 12.5. ESI-HRMS: *m/z* $[M+H]^+$calcd., 849.3678; found, 849.3716.

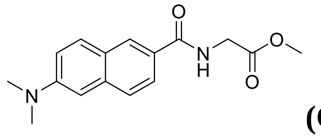

(C1FS dye, DAN)

To the solution of glycine methyl ester hydrochloride (276 mg, 0.22 mmol) and 6-(dimethylamino)-2-naphthoic acid (430 mg, 0.2 mmol) in THF was added EDC ·HCl (460 mg, 0.24 mmol) at 0 °C. After addition of HOBt (61 mg, 0.04 mmol) and DIEA (0.115 mL, 0.66 mmol), the reaction was stirred for 10 min at 0 °C before the temperature was raised to rt. When the reaction was completed, the solvent was evaporated and the residue was diluted with EA and washed with saturated NH_4Cl, Na_2CO_3 and brine before dried over Na_2SO_4 and concentrated. The residue was purified with flash chromatography (EA:Hexane = 1:2) to afford the product as light green solid (40.4 mg, 70.6% yield).**^{1}H NMR** (300 MHz, $CDCl_3$): δ 8.19 (d, *J* = 1.5 Hz, 1H), 7.76 (d, *J* = 2.8 Hz, 1H), 7.73 (d, *J* = 2.0 Hz, 1H), 7.64 (d, *J* = 8.7 Hz, 1H), 7.16 (dd, *J* = 9.1, 2.6 Hz, 1H), 6.88 (d, *J* = 2.4 Hz, 1H), 6.81 (t, *J* = 8.2 Hz, 1H), 4.29 (d, *J* = 5.1 Hz, 2H), 3.80 (s, 3H), 3.08 (s, 6H). **^{13}C NMR** (75 MHz, $CDCl_3$): δ 170.8, 167.7, 149.7, 136.8, 130.0, 127.6, 126.4, 126.3, 125.3, 123.9, 116.5, 105.4, 52.4, 41.8, 40.5. **IT-TOF-MS**: *m/z* $[M+H]^+$ calcd, 287.13; found, 287.14.

Determination of Photophysical and Biochemical Properties of Probes *in vitro*

Photophysical measurements. All compounds were diluted in HEPES buffer (50 mM HEPES, 50 mM NaCl, 0.1% CHAPS, 10 mM EDTA, 5% glycerol, 10 mM DTT, *p*H = 7.2) and the measurements were performed at room temperature. For **C1FS**, 6-(dimethylamino)-2-naphthoic acid coupled with glycine methyl ester (**DAN**) served as its free dye for photophysical measurements in consideration of easy preparation. UV-vis absorption was measured at a concentration of 10 μM in 1-cm standard quartz cells. And fluorescence spectra were measured at 1 μM. The fluorescence quantum yield (Φ) was determined by using fluorescein aqueous NaOH (*p*H 13) as the reference through $\Phi_s = \Phi_r(n_s^2E_sA_r)/(n_r^2E_rA_s)$, where the subscripts *s* and *r* refer to the sample and reference compound, respectively. *E* is the integrated area under the emission spectrum. *A* is the absorbance of the solution at the excitation wavelength and *n* is the refractive index of the solvent. The two-photon excited fluorescence intensity was measured from 760 to 850 nm at 1 μM concentration of the compound. The two-photon action cross section (TPA cross section, $\delta\Phi$) of each compound was calculated with **AAN** as the reference, whose two-photon properties have been reported in the literature.[7] The TPA cross section was calculated by using $\delta_s\Phi_s= \delta_r\Phi_r(S_s\varphi_rc_r)/(S_r\varphi_sc_s)$. The intensity of the detected signal was denoted as *S*. φ is the overall fluorescence collection efficiency of the experimental apparatus. The number density of the molecules in solution was denoted as *c*. δ_r is the two-photon absorption cross

section of the reference molecule.

Enzyme kinetics. All enzymatic assays were carried out in HEPES buffer (as described above) supplemented with 0.02% triton. Detailed kinetic properties of these probes were measured with target recombinant caspases using commercial tetrapeptide-AMC fluorogenic substrates as references at 25 ℃ in 384-well plates (Greiner Bio-One #781900). Same amount of a caspase was added to different concentrations of the probe or the commercial caspase substrate (tetrapeptide-AMC) in a total volume of 25 μL. Liberation of fluorescence (λ_{ex} = 360 ± 40 nm; λ_{em} = 528 ± 20 nm for **AAN**, λ_{em} = 460 ± 40 nm for AMC and **DAN**) was monitored continuously using a BioTek Synergy 4 plate reader. All measurements were performed in duplicate. Kinetic constants (K_M, K_{cat} and K_{cat}/K_M) were computed by direct fits of the data to the Michaelis-Menton Equation using a non-linear regression *via* Origin software (Supplementary Table 1 and Supplementary Fig. 2).

Hydrolytic stability assay with C1RS/C1FS. The hydrolytic stability assay was carried out as described previously.[8] BV2 cell lysates were prepared to a final concentration of 2 μg/μL. The compound (**C1RS** or **C1FS**) was added into the cell lysates to a final concentration of 100 μM. After shaking at 150 rpm for 24 h at 25 °C, a small portion of the reaction was analyzed by LC-MS (IT-TOF). As expected, the proteolytic stability test on both **C1RS** and **C1FS** showed they did not display any noticeable degradation in BV2 lysates over 24 h (Supplementary Fig.3). All issues considered, **C1RB** and **C1FS** were therefore chosen as the designated caspase-1 detecting probes in subsequent bioimaging studies.

In vitro selectivity. For each probe, different caspases (-1, -3, -6, -8 and -9) were applied under identical conditions. Normalized, relative fluorescence values (RFU) were obtained after 2-h incubation at room temperature with λ_{ex} = 360 ± 40 nm; λ_{em} = 528 ± 20 nm for **AAN**/λ_{em} = 460 ± 40 nm for AMC and **DAN**. All probes showed virtually identical selectivity profiles towards their intended caspases as those obtained with the AMC substrates. We did not include recombinant caspase-7 in the screening, as its reactivity toward our probes would be redundantly similar to that of caspase-3, based on published literature.[9, 10] Finally, both **C6RA** and **C9RA**, similar to their AMC counterparts, exhibited comparatively poor selectivity profiles towards caspase-6 and -9, respectively, and were unlikely to be useful in real bioimaging applications. Taken together, in addition to **C1RB** and **C1FS** (for caspase-1 bioimaging), we chose **C3RA**/**C3RM**/**C3RE** and **C8RA** for further two-photon, live-cell bioimaging of caspase-3/-7 and caspase-8 activities, respectively, as these probes possess both

reasonable *in vitro* reactivity and selectivity profiles towards their intended caspase targets.

Detection of caspase activities in mammalian cells and tissues

Cell culture. The mouse embryonic fibroblast cell line (NIH/3T3), mouse microglial cell line (BV2) and human epithelial carcinoma cell line (HeLa) used in our experiments were cultured in Dulbecco's Modified Eagle's Medium (DMEM) supplemented with 10% (for NIH/3T3 and HeLa) or 5% (for BV2) fetal bovine serum (FBS), 100.0 mg/L streptomycin and 100 IU/mL penicillin in a humidified atmosphere of 5% CO_2 at 37 ^oC.

XTT assay. The cytotoxicity of our probes was determined by using the XTT colorimetric cell proliferation kit (Roche) following manufacturer's guidelines. Briefly, NIH/3T3, BV2 and HeLa cells were seeded in 20~30% confluency (since they will reach >50% confluency within 24 h) into 96-well plates and incubated overnight before the addition of probes with concentrations of 12 μM, 30 μM, 60 μM and 120 μM, respectively. Probes were applied from DMSO stocks whereby DMSO never exceeded 1% in the final solution. The same volume of DMSO was used as a negative control; and the same volume of STS (200 nM) was used as a positive control. After a total treatment time of 24 h, proliferation was assayed using the XTT assay (Supplementary Fig. 4).

General procedures for confocal microscopy of mammalian cells. All 2D images were acquired on Leica TCS SP5X Confocal Microscope System (one-/two-photon; λ_{ex} = 405 nm and 760 nm, respectively, with PMT settings of λ_{em} = 480-550 nm for **AAN** probes, λ_{em} = 450-550 nm for **C1FS**, λ_{em} = 420-500 nm for tetrapeptide-AMC) or Carl Zeiss LSM 510 Meta Confocal Microscope (one-photon images in Fig. 3j; λ_{ex} = 405 nm, λ_{em} = 470-500 nm for **AAN** probes). 3D images for immunofluorescence were acquired from PerkinElmer Ultraview Vox Spinning Disc confocal microscope (one-photon) and processed with Volocity 6.3.1 3D image analysis software. Cells were seeded in glass-bottom dishes (Greiner Bio-One, #627870) and maintained in a humidified atmosphere of 5% CO_2 at 37 ℃ overnight before being processed for imaging. For quantification of fluorescence signal intensity, each set of data were independently normalized to the non-induced channel (set to 0) and induced channel (set to 1) of the **C3RA** readouts.

Permeability of free dye and probes in live cells. To check the permeability and distribution of the free dye (i.e., **AAN**) and our probes in live cells, HeLa cells were seeded in the glass-bottom dishes and grown to be 70% confluency. Then each compound at a suitable concentration was added into

HeLa cells and incubated for 1 h at 37 °C before images were taken. LysoTracker (100 nM, LysoTracker® Red DND-99, L-7528) was added together to check the cellular localization of the compound. The free dye **AAN** was microscopically observed to diffuse freely throughout the entire intracellular environment (λ_{em} = 470-500 nm; Supplementary Fig. 5a). By taking advantage of the weakly blue-shifted fluorescence of **AAN**-modified probes (**C1RB**/**C3RA**/**C8RA**; λ_{ex} = 405 nm, λ_{em} = 420-480 nm with Carl Zeiss system), we were able to determine they were cell-permeable (Supplementary Fig. 5a). Similar AMC-containing probes are known to be cell-permeable as well. Furthermore, incubation at low temperature (4 °C) of selected probe (i.e., **C3RA**) was also carried out to check whether the entrance of the probe was endocytosis-dependent. HeLa cells were pretreated with DMSO/STS (1 μM) for 4 h before the addition of **C3RA** (24 μM) for another 1 h either at 4 °C or 37 °C. One-photon excited imaging (λ_{ex} = 405 nm) was then carried out with λ_{em} = 420-465 nm (indicative of the presence of these probes; Supplementary Fig. 5c, up) and λ_{em} = 480-550 nm (indicative of the presence of free dye, **AAN**; Supplementary Fig. 5c, down), and no obvious endocytosis and endo/lysosomal trapping was observed. To be noted, for **C1FS**, we were not able to obtain direct imaging-based evidence of its intracellular localization profiles due to its nearly non-fluorescent state, but given its small uniform molecule-like feature, and that its analog **C1RS** was cell-permeable, we expected this probe to be taken up readily by cells as well.

Transfection of active caspases into NIH/3T3 cells. The transfection was carried out following the manufacturer's guidelines (Thermo Scientific, #89850). Briefly, 5×10^4 cells per compartment were seeded into the 4-well glass-bottom imaging dish and left growing overnight. The total Pro-Ject™ Reagent was dissolved in 250 μL methanol and vortexed for 10-20 seconds at top speed. Then 2.5 μL (for each compartment of the imaging dish) of it was pipetted into the bottom of each microcentrifuge tube. Methanol was evaporated under a laminar flow hood for 4 h. Active caspases (50 ng caspase-1, 15 ng caspase-3 or 5 ng caspase-8) were diluted in 20 μL PBS. The dried Pro-Ject™ was hydrated with the diluted protein solution and pipetted up and down 3-5 times followed by incubation at room temperature for 3-5 min. Then the mixture was vortexed for a few seconds at low to medium speed. The final volume of the Pro-Ject™ Reagent/protein mixture was brought to 200 μL with serum-free DMEM and transferred directly onto NIH/3T3 cells. After incubation in a 5% CO_2 incubator at 37 °C for 4 h, the mixture was removed and cells were washed gently with PBS. Afterwards, 500 μL fresh DMEM (+ 10% FBS) containing probes (24 μM **C1RB**/12 μM **C1FS** for caspase-1; 24 μM **C3RA** for

caspase-3; 24 μM **C8RA** for caspase-8) was applied for 1 h before the membrane tracker (25 ng/mL, CellMask™ Deep Red plasma membrane stain, C10046) was incubated for 10 min at room temperature. Then the medium was discarded and cells were washed for imaging. For inhibition treatment, once the 4-h transfection was complete, 50 μM Z-VAD(OMe)-FMK in fresh DMEM was added 1 h before the probe. λ_{ex} = 760 nm, λ_{em} = 480-550 nm (except for **C1FS** with λ_{em} = 450-550 nm) for probes and λ_{ex} = 650 nm, λ_{em} = 655-740 nm for the membrane tracker (Supplementary Fig. 5b).

Staurosporine (STS)-induced caspase activation in mammalian cells. Induction of apoptosis by STS in mammalian cells was performed as previously described.[5, 9, 11] Cells were grown to be ~70% confluency before treatment with STS (1.0 μM in HeLa, 2.0 μM in BV2) for 4 h to induce apoptosis. Activation of caspases was detected by in-cell imaging or enzymatic assays using our probes as well as Western blotting (WB). For live-cell imaging, HeLa/BV2 cells in glass-bottom imaging dishes were pretreated with/without inhibitor (50 μM Z-VAD(OMe)-FMK) for 1 h before stimulation with STS or equivalent volume of DMSO for 4 h. **C3RA**/**C8RA** (24 μM as final concentration) was subsequently added and incubated for another 1 h before imaging. One-photon (λ_{ex} = 405 nm) and two-photon (λ_{ex} = 760 nm) excitated fluorescence imaging were carried out using confocal microscope with PMT range = 480-550 nm for **AAN** containing probes, 450-550 nm for **DAN** and 420-500 nm for AMC with Leica system. For continuously real-time imaging (λ_{ex} = 405 nm), HeLa/BV2 cells were incubated with the probe for 1 h in advance followed by the addition of STS (2.0 μM), and time-course imaging begun thereafter. For **C3RM** and **C3RE**, in order to remove free probes outside of the targeted organelle after 1-h incubation, cells were washed with FBS-free DMEM before STS was added.

Lysates preparation. Lysates for enzymatic assay and Western blotting were prepared in the same way. After treatment, cells were scraped and the pellets were washed with cold PBS. Then cells were lysed in lysis buffer (HEPES buffer with 0.02% Triton X-100 and 0.1 mM phenylmethylsulfonyl fluoride (PMSF)) by sonication (Sonics, VCX 130). The supernatant was obtained by centrifugation at 13,000 rpm for around 20 min at 4 ^{o}C and protein concentration was determined by using Bradford protein assay (DC Protein Bioassay Kit, Bio-Rad).

Western blotting (WB). Equal amount of lysates diluted in SDS loading buffer was separated by 15% SDS-PAGE before transferred onto a Polyvinylidene Fluoride Transfer Membrane (BioTrace™ PVDF membrane). The PVDF membrane was then blocked with 5% low fat milk or 3% BSA in 1× PBS containing 0.1% Tween-20 (PBST) for 1 h at room temperature. Then the membrane was probed

with certain primary antibody (1:1000) and shaken at room temperature for 1 h or 4 ℃ overnight. HRP conjugated secondary antibody (1:5000) was incubated at room temperature for 1 h. The antibody-reactive bands were visualized by chemiluminescent detection (SuperSignal™ West Dura Chemiluminescent Substrate from Thermo Scientific™).

Enzymatic assay with lysates. In an enzymatic assay, the lysates (around 30 μg in 20 μL per well) were mixed with the probe (6 μM as final concentration; 5 μL of 30 μM probe per well) in a 384-well plate (Greiner Bio-One #781900) and reacted at 37 ^{o}C for 1-2 h. The increase in fluorescence was recorded by BioTek Synergy 4 plate reader (λ_{ex} = 360 ± 40 nm; λ_{em} = 528 ± 20 nm for **AAN** probes). For inhibition experiments, the lysates were pretreated with 50 μM Z-VAD(OMe)-FMK for 3 h at 4 ^{o}C before reaction with probes. Each set of data were independently normalized to the STS (-)/Z-VAD-FMK (-) channel (set to 0) and STS (+)/Z-VAD-FMK (-) channel (set to 1) of the **C3RA** readouts.

Immunofluorescence imaging of active caspase-3 in STS induced HeLa cells. HeLa cells were grown to be ~70% confluency in glass-bottom imaging dishes and stained with Mitotracker (18 nM, MitoTracker® Red CMXRos, M7512) for 1 h at 37 ^{o}C. Upon washing with FBS-free DMEM and re-introduction of FBS-free DMEM, cells were treated with STS (2.0 μM) for 2 h to induce apoptosis. After washing with PBS, cells were fixed with 3.7% formaldehyde in PBS for 1 h at 37 ℃, washed twice with PBS again and permeabilized with 0.02% Triton X-100 in PBS for 10 min at room temperature, washed twice with PBS, blocked with 2% BSA in PBS for 30 min at room temperature, followed by incubation with anti-active caspase-3 (1:200, CST #9664) for 4 ℃ overnight. Upon washing with PBS (containing 0.02% Triton X-100) twice for 10 min each and incubation with Cy5-conjugated Goat anti-Rabbit IgG (H+L) Secondary Antibody (1:500, Life technologies A10523) for 2 h at room temperature, the cells were then washed with PBS (containing 0.02% Triton X-100) three times for 10 min each and washed with PBS for 5 min with gentle agitation and a final wash with deionized water (1~2 min with gentle agitation). Then cells were co-stained with Hoechst 33342 (0.3 μg/mL, Life technologies H1399) for 10 min followed by ER-Tracker (300 nM, ER-Tracker™ Green, Life technologies E34251) for another 1 h at room temperature. Cells were washed again with PBS before image acquisition. 2D images were acquired on a Leica TCS SP5X Confocal Microscope System (Supplementary Fig. 6). To better visualize the cellular signals, 3D images were acquired from PerkinElmer Ultraview Vox Spinning Disc confocal microscope and processed with Volocity 3D image analysis software (Fig. 3k and Supplementary Videos).[12, 13]

TPFM imaging of caspase activities in primary cortical neurons and fresh tissues. All two-photon images were taken on a Leica TCS SP5X Confocal Microscope System. Procedures involving animals were approved by and conformed to the guidelines of Institutional Animal Care and Use Committee at the National Neuroscience Institute (Singapore).

Dissociated neuron-enriched cell cultures of cerebral cortex were established from day 16 C57BL/6 mouse embryos, as described.[14] Primary neurons were cultured in the neurobasal medium (Gibco) with 2% B27 and 0.5 mM GlutaMAX. Experiments were performed in 7-9 day-old cultures. For glucose-deprivation (GD) studies, glucose-free neurobasal medium was used (with other components fixed). The cultured neurons were incubated in glucose-deprived medium for 8 h, while control cells were incubated in normal neurobasal medium. Afterwards, **C1RB** (24 μM)/**C1FS** (12 μM) was introduced with further incubation for 1 h at 37 ^{o}C. To check whether the signal observed was related to caspase-1 activity, the caspase-1 specific inhibitor, Pralnacasan (50 μM), was added to the cultures 1 h prior to probe introduction (t = 7 h during GD) (Supplementary Fig. 7a). To verify the presence of cleaved caspase-1, WB of total lysates from GD treated primary neurons for different time periods were performed as well (Supplementary Fig. 7b).

Tissues used in the imaging experiments were fresh brains of 7-day-old C57BL/6 mice subcutaneously injected with either ethanol (20% solution in normal saline with 2.5 g/kg at 0 h and again at 2 h) or the same amount of normal saline.[15, 16] At 8 h following the first ethanol dose, the brains were surgically removed from the mouse head and immediately transferred into an ice-artificial cerebrospinal fluid (ACSF; 138.6 mM NaCl, 3.5 mM KCl, 21 mM $NaHCO_3$, 0.6 mM NaH_2PO_4, 10 mM D-glucose, 1 mM $CaCl_2$ and 3 mM $MgCl_2$). The brain was cut into 200 μm-thick sections using a vibrating blade microtome in ACSF. Slices were incubated with **C3RA** (120 μM) in ACSF at 37 ^{o}C for 3 h before image acquisition. For inhibition experiments, the slices were treated with caspase-3/7 inhibitor I (200 μM) 3 h prior to addition of **C3RA**. Treated brains were then transferred to poly-L-lysine-coated cover slips and images were acquired at different depths by changing the Z-axis thickness on the microscope. To prepare lysates, the brain was first homogenized in lysis buffer (HEPES containing 1% triton X-100 and 1 mM PMSF) with plastic homogenizer and then sonicated. The supernatant was obtained by centrifugation at 13,000 rpm for 20 min at 4 ^{o}C and protein concentration was determined. 30 μg lysates per lane were loaded for WB (Fig. 4c). At the same time, 300 μg lysates in 60 μL was taken for enzymatic assay (diluted in HEPES containing 0.02% triton X-100) with 6 μM

C1RB/C3RA/C8RA (15 μL of 30 μM probe per well) in 384-well plate at 37 ^{o}C (Fig. 4d).

Supplementary References

1. Linton S. D. Caspase inhibitors: a pharmaceutical industry perspective. *Curr. Top. Med. Chem.* **5**, 1697-1716 (2005).
2. Kim H. M., *et al.* Environment-sensitive two-photon probe for intracellular free magnesium ions in live tissue. *Angew. Chem. Int. Edit.* **46**, 3460-3463 (2007).
3. Kim H. M., Kim B. R., An M. J., Hong J. H., Lee K. J., Cho B. R. Two-photon fluorescent probes for long-term imaging of calcium waves in live tissue. *Chem. Eur. J.* **14**, 2075-2083 (2008).
4. Wube A. A., *et al.* Design, synthesis and antimycobacterial activities of 1-methyl-2-alkenyl-4(1H)-quinolones. *Bioorgan. Med. Chem.* **19**, 567-579 (2011).
5. Li J., Yao S. Q. "Singapore Green": a new fluorescent dye for microarray and bioimaging applications. *Org. Lett.* **11**, 405-408 (2009).
6. Zhang C.-J., Li L., Chen G. Y. J., Xu Q.-H., Yao S. Q. One- and two-photon live cell imaging using a mutant SNAP-tag protein and its FRET substrate pairs. *Org. Lett.* **13**, 4160-4163 (2011).
7. Chung C., Srikun D., Lim C. S., Chang C. J., Cho B. R. A two-photon fluorescent probe for ratiometric imaging of hydrogen peroxide in live tissue. *Chem. Commun.* **47**, 9618-9620 (2011).
8. Ge J., Wu H., Yao S. Q. An unnatural amino acid that mimics phosphotyrosine. *Chem. Commun.* **46**, 2980-2982 (2010).
9. Hu M., *et al.* Multicolor, one- and two-photon imaging of enzymatic activities in live cells with fluorescently quenched activity-based probes (*q*ABPs). *J. Am. Chem. Soc.* **133**, 12009-12020 (2011).
10. McStay G. P., Salvesen G. S., Green D. R. Overlapping cleavage motif selectivity of caspases: implications for analysis of apoptotic pathways. *Cell Death Differ.* **15**, 322-331 (2008).
11. Wu H., Ge J., Yang P.-Y., Wang J., Uttamchandani M., Yao S. Q. A peptide aldehyde microarray for high-throughput profiling of cellular events. *J. Am. Chem. Soc.* **133**, 1946-1954 (2011).
12. Li L, *et al.* A small-molecule probe for selective profiling and imaging of monoamine oxidase B activities in models of Parkinson's disease. **54**, 10821-10825 (2015).
13. Yu C., Qian L., Uttamchandani M., Li L., Yao S. Q. Single-vehicular delivery of antagomir and small molecules to inhibit miR-122 function in hepatocellular carcinoma cells by using "smart" mesoporous silica nanoparticles. *Angew. Chem. Int. Edit.* **54**, 10574-10578 (2015).
14. Fann D. Y., *et al.* Intravenous immunoglobulin suppresses NLRP1 and NLRP3 inflammasome-mediated neuronal death in ischemic stroke. *Cell Death Dis.* **4**, e790 (2013).
15. Olney J. W., *et al.* Ethanol-induced caspase-3 activation in the *in vivo* developing mouse brain. *Neurobiol. Dis.* **9**, 205-219 (2002).
16. Young C., *et al.* Ethanol-induced neuronal apoptosis *in vivo* requires BAX in the developing mouse brain. *Cell Death Differ.* **10**, 1148-1155 (2003).
